# Ionizing Radiation Dose Differentially Affects the Host–Microbe Relationship over Time

**DOI:** 10.3390/microorganisms12101995

**Published:** 2024-09-30

**Authors:** Nabarun Chakraborty, Allison Hoke, Ross Campbell, Gregory Holmes-Hampton, Vidya P. Kumar, Candace Moyler, Aarti Gautam, Rasha Hammamieh, Sanchita P. Ghosh

**Affiliations:** 1 Medical Readiness Systems Biology, Walter Reed Army Institute of Research, Silver Spring, MD 20910, USA; nabarun.chakraborty2.civ@health.mil (N.C.); allison.v.hoke.civ@health.mil (A.H.); ross.a.campbell18.ctr@mail.mil (R.C.); candace.c.moyler.ctr@health.mil (C.M.); aarti.gautam.civ@health.mil (A.G.); 2 The Geneva Foundation, Walter Reed Army Institute of Research, Silver Spring, MD 20910, USA; 3 Armed Forces Radiobiology Research Institute, Uniformed Services University of the Health Sciences (USUHS), Bethesda, MD 20889, USA; gregory.holmes-hampton@usuhs.edu (G.H.-H.); vidya.kumar.ctr@usuhs.edu (V.P.K.)

**Keywords:** total body irradiation, fecal microbiome, fecal metabolite, host–microbiome association, 16S rRNA gene sequencing, functional metagenome, descending colon contents

## Abstract

Microorganisms that colonize in or on a host play significant roles in regulating the host’s immunological fitness and bioenergy production, thus controlling the host’s stress responses. Radiation elicits a pro-inflammatory and bioenergy-expensive state, which could influence the gut microbial compositions and, therefore, the host–microbe bidirectional relationship. To test this hypothesis, young adult mice were exposed to total body irradiation (TBI) at doses of 9.5 Gy and 11 Gy, respectively. The irradiated mice were euthanized on days 1, 3, and 9 post TBI, and their descending colon contents (DCCs) were collected. The 16S ribosomal RNAs from the DCCs were screened to find the differentially enriched bacterial taxa due to TBI. Subsequently, these data were analyzed to identify the metagenome-specific biofunctions. The bacterial community of the DCCs showed increased levels of diversity as time progressed following TBI. The abundance profile was the most divergent at day 9 post 11 Gy TBI. For instance, an anti-inflammatory and energy-harvesting bacterium, namely, *Firmicutes*, became highly abundant and co-expressed in the DCC with pro-inflammatory *Deferribacteres* at day 9 post 11 Gy TBI. A systems evaluation found a diverging trend in the regulation profiles of the functional networks that were linked to the bacteria and metabolites of the DCCs, respectively. Additionally, the network clusters associated with lipid metabolism and bioenergy synthesis were found to be activated in the DCC bacteria but inhibited in the metabolite space at day 9 post 11 Gy. Taking these results together, the present analysis indicated a disrupted mouse–bacteria symbiotic relationship as time progressed after lethal irradiation. This information can help develop precise interventions to ameliorate the symptoms triggered by TBI.

## 1. Introduction

Radiation exposures, even with low doses, can adversely impact human health, causing various clinical symptoms that are referred as acute radiation syndrome (ARS) [1]. ARS manifests different symptoms depending on the radiation doses; for instance, a low-dose exposure causes hematopoietic sub-syndrome, and higher doses of radiation damage the gastrointestinal system and central nervous system [2,3]. In addition, ARS results in a high rate of mortality. A comprehensive understanding of the ARS-induced molecular changes is warranted in order to develop an effective therapeutic strategy for ARS. 

Emerging results have identified microorganisms that colonize in and on the host and act as the key regulators in combating stress or exposure to foreign stimuli [4]. Mostly concentrated inside the human intestine, the microbial community is primarily dominated by bacteria (10^9^–10^11^ cells/g) [5,6], archaea (10^8^–10^10^ cells/g) [7], and viruses (10^5^–10^6^ cells/g) [8]. Together, these gut microbes foster a balanced ecosystem with humans, as they complement each other in accomplishing a wide range of functions, including metabolic and digestive functions, immune fitness, and synaptic activities, thus regulating cognitive and overall psycho-physiological constructs [9,10,11,12]. There is a great deal of emerging interest in understanding the roles of microbes in radiation pathogenesis, assessment of exposure, and rescuing subjects from radiation-induced mortality [9]. 

Indeed, the role of microbes in combating radiation exposure was suggested many years back when germ-free mice were found to be resistant to radiation exposure that typically causes hematopoietic syndrome [13], and this observation was validated recently [14]. An independent study linked a total body irradiation (TBI)-induced surge of pro-inflammatory cytokines to a shift in the ecosystem of intestinal microbes [15]. The association of gut flora and TBI was further underscored by a recent study that identified a group of bacterial taxa, which were selectively proliferated in mice that had survived lethal radiation [16]. Low-dose irradiation can trigger severe mitochondrial damage, and the residual mitochondrial contents that cannot be removed by mitophagy can trigger inflammation [17]. The inflammatory signals produced by activated microglia and infiltrated immune cells promote intracellular signaling cascades and ultimately alter the host metabolism [17]. Since resident microbes are key producers of bioenergy [18], one can postulate that the host–microbiome symbiotic association plays a major role in regulating the networks linked to bioenergy coupled with inflammation and metabolism [19]. 

It is noteworthy that the bacterial communities that are colonized in both humans and rodents share a highly qualitative but minimally quantitative similarity [20], although both communities are dominated by four bacterial phyla: namely, *Firmicutes*, *Bacteroidetes*, *Proteobacteria*, and *Actinobacteria* [3]. The quantitative disparity between the microbial populations that are colonized in human and rodent intestines, respectively, is likely to be a hurdle in finding a phylogenetically conserved solution of translational potential. To overcome this challenge, we plan to exploit the metabolite profile that is at the converging node of multiple kingdoms, including hosts and their resident microbes [21,22]. Current technological capabilities can detect more than 200,000 endogenous metabolites that are linked to ~1900 enzymes encoded in the human genome [23]. In addition, there are around 14,000 metabolites that are synthesized in both humans and microbes. In comparison, there are around 1800 metabolites that are specifically synthesized by the microbial community [22,23]. 

Available evidence [14,15,24,25] has helped build the hypothesis that ARS adversely impacts the host–microbe relationship; hence, a strategic intervention to foster this relationship can mitigate ARS. In this context, the objective of the present work was to identify those taxa that are differentially enriched by a range of radiation doses and the time since TBI exposure and to map this differentially enriched bacterial community toward finding the associated biological functions. In addition, we aimed to construct a DCC-metabolite-enriched network that potentially mirrors the co-perturbations in irradiated mice and bacteria [22]. These two sets of networks were overlapped to inform how TBI putatively disrupts the symbiotic relationship between mice and the DCC bacterial community. Integrating this network information to the rodents’ phenotype, we suggested that there was a likelihood that with the increasing radiation dose, the DCC microbes gradually withdrew from the bidirectional relationship with the host mouse [13,14].

## 2. Materials and Methods

### 2.1. Animals, and Veterinary Care

Male CD2F1 mice (10 to 11 weeks old) were purchased from Envigo (Indianapolis, IN, USA) and acclimated for one to two weeks prior to the start of the study. Experimental animals were identified by either tail tattoos or ear tags and were housed in groups of up to 5 in plastic cages in Allentown NexGen cage systems as described earlier [26]. The animals received Harlan Teklad Rodent Diet 8604 and acidified water (pH 2.5–3.0) ad libitum as soon as they arrived and for the duration of the study. The animal rooms were maintained at 21 ± 2 °C and 50 ± 10% relative humidity with 10–15 cycles of fresh air cycled hourly and a 12:12 h light:dark cycle. 

### 2.2. Total Body Gamma Irradiation Exposure and Monitoring Clinical Signs of Radiation

Twelve- to fourteen-week-old male CD2F1 mice were irradiated bilaterally [27] in the Armed Forces Radiobiology Research Institute’s (AFRRI) cobalt-60 gamma radiation facility in well-ventilated plexiglass boxes (8 mice per box) at a dose rate of ~0.6 Gy/min to total midline doses of 9.5 Gy and 11 Gy, respectively [28]. The dose rate of ~0.6 Gy/min was selected due to the ability to provide a prompt total dose within a short period of time, ~15–20 min, minimizing the time that animals are confined in the radiation boxes. After irradiation, mice were returned to their original cages with free access to food and water ad libitum. All of the irradiations were performed in the morning to minimize the diurnal effect. Animals were monitored closely three times daily (early morning 6:00–8:00, afternoon 13:00–15:00, and late evening 20:00–22:00) by trained personnel because mice start showing signs of pain and distress from radiation damage a few days following exposure. This period is termed a “critical period”, and a health score was given at each time of monitoring in accordance with pre-defined criteria described and approved in the IACUC protocol [26]. Radiation-induced pain and distress were monitored using several criteria including unresponsiveness, abnormal posture, unkempt appearance, immobility, labored breathing, respiratory distress, and lack of coordination [29]. A mouse showing any of the following symptoms was determined to be moribund: inability of the mouse to right itself, limb paralysis, abdominal breathing, a constant twitching, trembling, or tremor that lasted for more than 10 s, or greater than 35% weight loss. Moribund mice were humanly euthanized immediately as per IACUC policy. 

### 2.3. Sample Collection Post-Euthanasia

**Figure 1:**
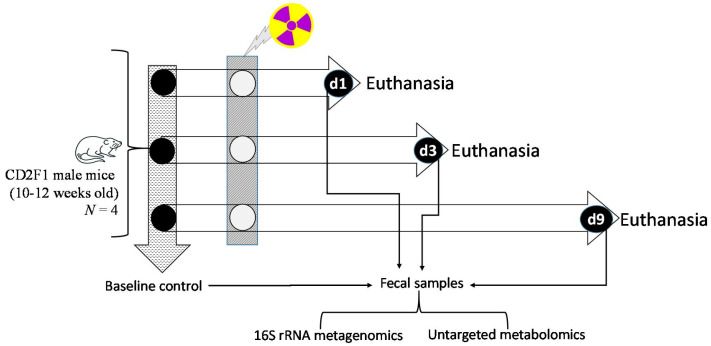
Study design. CD2F1 male mice were exposed to two different doses of total body irradiation (TBI), namely 9.5 Gy and 11 Gy. Descending colon contents (DCCs), which were collected one day before irradiation, were used as the baseline control (CTR). Subsequently, DCCs were collected day 1 (d1), 3 (d3), and 9 (d9) post-TBI. Aliquots from the DCCs were used for 16S rRNA gene sequencing and untargeted metabolomics assays.

The DCCs were harvested post-euthanasia at pre-determined timepoints [26] and were collected from unirradiated animals (baseline control or CTR) and irradiated animals on day 1 (d1), day 3 (d3), and day 9 (d9) post-irradiation (Figure 1). Post-euthanasia, the entire intestine was incised on ice, and the DCCs was extracted from the descending colon tissue, cryogenically homogenized, aliquoted for 16S rRNA gene sequencing and metabolomics assays, respectively, and placed at −80 °C for long-term storage. 

The liver tissue was harvested and snap frozen immediately from six CTR mice and six irradiated animals on d3 and d9 post-irradiation at 9.5 Gy and 11 Gy.

### 2.4. Descending Colon Contents 16S rRNA Gene Sequencing Assay

Following our previously published method, RNA and DNA was extracted from the first aliquot of DCC samples using the Power Soil DNA Isolation Kit (MoBio Laboratories, Inc., Carlsbad, CA, USA). The extracted DCC DNA was used for the 16S rRNA gene sequencing study. To meet this goal, the Illumina 16S Metagenomics Library Preparation manual (Illumina, Inc., San Diego, CA, USA) was followed. Briefly, we used previously designed primers to isolate the hyper-variable V3 and V4 region of the 16S rRNA amplicon [30], the samples were barcoded using Nextera indexes, and the libraries were pooled and sequenced on the Illumina MiSeq platform, using paired-end 300 bp reads and Illumina MiSeq v3 reagents. 

### 2.5. Untargeted Metabolomics of Descending Colon Contents

The second aliquot of DCC samples was used for untargeted metabolomics profiling following our standard protocol [31,32]. For the metabolomics sample preparation, 150 µL of an extraction solution containing internal standards made up of 5 mL water, 5 mL methanol, 10 µL debrisoquine (1 mg/mL in ddH2O), and 50 µL of 4-nitrobenzoic acid (1 mg/mL in methanol) (per 10 mL) was added to the DCCs. The samples were vortexed for 15 min and then incubated on ice for 20 min. Next, 150 µL of chilled acetonitrile was added, the samples were vortexed, and then they were incubated at −20 °C for 20 min. Lastly, the samples were centrifuged at 15,493× *g* for 20 min at 4 °C, and the supernatant was transferred to an MS vial for LC-MS analysis. Next, 2 µL of each prepared sample was injected onto a Waters Acquity BEH C18 1.7 μm, 2.1 × 50 mm column using an Acquity UPLC system coupled to a Xevo G2-S quadrupole-time-of-flight mass spectrometer with an electrospray ionization source (UPLC-ESI-QToF-MS) (Waters Corporation, Milford, MA, USA). The mobile phases consisted of 100% water (solvent A), 100% acetonitrile containing 0.1% formic acid (solvent B), and 100% isopropanol with 0.1% formic acid (solvent C). The solvent flow rate for the metabolomics acquisition was set to 0.4 mL/min with the column set at 60 °C. The LC gradient was as follows: initial—95% A, 5% B; 0.5 min—95% A, 5% B; 8.0 min—2% A, 98% B; 9.0 min—11.8% B, 88.2% C; 10.5 min—11.8% B, 88.2% C; 11.5 min—50% A, 50% B; 12.5 min—95% A, 5% B; 13.0 min—95% A, 5% B. The column eluent was introduced into the Xevo G2-S mass spectrometer by electrospray operating in either negative or positive electrospray ionization mode. The positive mode had a capillary voltage of 3.00 kV and a sampling cone voltage of 30 V. The negative mode had a capillary voltage of 2.00 kV and a sampling cone voltage of 30 V. The desolvation gas flow was set to 600 L/h, and the desolvation temperature was set to 500 °C. The cone gas flow was 25 L/h, and the source temperature was set to 100 °C. The data were acquired in the sensitivity MS mode with a scan time of 0.300 s and an interscan time of 0.014 s. Accurate mass was maintained by infusing Leucine Enkephalin (556.2771 [M+H]^+^/554.2615 [M-H]^−^) in 50% aqueous acetonitrile (2.0 ng/mL) at a rate of 10 µL/min via the Lockspray interface every 10 s. The data were acquired in centroid mode with a 50.0 to 1200.0 *m*/*z* mass range for TOF-MS scanning. An aliquot of each sample was pooled and used as a quality control (QC), which represented all metabolites present. This QC sample was run at the beginning of the sequence to condition the column and then injected after every 10 samples to check mass accuracy, ensure the presence of internal standards, and monitor shifts in retention time and signal intensities.

The untargeted data acquired were first converted to the NetCDF unified data format using the Databridge tool in MassLynx (Waters Corporation, Milford, MA, USA). An in-house implementation of the XCMS R package (Scripps Institute, La Jolla, CA, USA) was used for peak detection with an ordered bijective interpolated warping algorithm utilized for retention time correction and parameters optimized using the Isotopologue Parameter Optimization (IPO) R package [33]. The mass-to-charge ratio and retention time (mzrt) features were normalized based on the internal standards (debrisoquine and 4-nitrobenzoic acid present in the extraction solution in positive and negative modes, respectively) as well as QC-RLSC (QC robust LOESS signal correction) normalization.

### 2.6. Quantitative Detection of Lipopolysaccharide Binding Protein (LBP) in Liver

First, 30 to 40 mg of liver tissue was lysed in ice-cold Pierce IP Lysis Buffer (Thermo Fisher Scientific, Waltham, MA, USA; 87788) containing 1:100 dilution of protease inhibitor cocktail (Sigma-Aldrich, St. Louis, MO; P8340-1ML) using a hand-held homogenizer. To extract the proteins, homogenates were incubated at 4 °C for 10 min and then centrifuged at 16,000× *g* for 15 min at 4 °C. Next, protein concentration was determined using the Pierce Bicinchoninic Acid (BCA) Protein Assay Kit (Thermo Fisher Scientific; 23225) followed by quantitative detection of mouse LBP using the Mouse LBP ELISA Kit (Abcam, Cambridge, MA, USA; ab213876). The standard protocol of the ELISA was slightly modified by blocking the biotin using the Endogenous Biotin-Blocking Kit (Thermo Fisher Scientific; E21390) in order to nullify the effect of the endogenous biotin in the liver, and the rest of the assay was performed following the manufacturer’s protocol.

### 2.7. Statistical Analysis and Data Integration

16S rRNA gene sequencing analysis: This analysis was conducted following our previously published protocols [34,35]. The raw fastq files were imported into R and processed using the DADA2 [36] package from Bioconductor [37], following the standard procedure on demultiplexed sequences. Briefly, the reads were inspected for quality, filtered, and truncated at 220 bp, and the paired sequencing reads were merged to generate a high-quality, full-length amplicon of approximately 460 bp of the V3 and V4 regions. A sequence table of amplicon sequence variants (ASVs) was constructed, the chimeras were removed, and the ASV table was imported into QIIME2 v.2019.7 [38].

Beta diversity was calculated using the Bray–Curtis and Euclidean algorithms [39], and the Principle Coordinate Analysis (PCoA) was estimated using q2-diversity. The Adonis plugin in QIIME2 [40,41] computed a two-way permutational multivariate analysis of variance (PERMANOVA), using the two co-factors of radiation dose (RD) and time since irradiation (TSI), respectively, with a significance cutoff at *p* < 0.05. All of the subsequent multi-factorial comparison analyses used the two co-factors, RD (9.5 Gy vs. 11 Gy) and TSI (d1, d3 and d9 post-TBI), and their cumulative model, e.g., RD × TSI. Alpha diversity was measured by the Simpson [42], Chao1 [43], and Shannon [44] indices. The alpha group significance across the co-factors, RD and TSI, was determined using two-way analysis of variance (ANOVA) and the post-Tukey test calculated in PRISM (GraphPad v.8, San Diego, CA, USA) with the significance value of *p* < 0.05. 

The taxonomic classification of the ASVs was generated by a q2-feature-classifier [45] by the classify-sklearn plugin [46]. The ASV database was mapped on the Silva reference database v132 with a 99% taxonomy reference to curate taxa [47]. Next, the linear discriminant analysis (LDA) effect size (LEfSe) curated the top-ranked taxonomic classifiers that discriminated 9.5 Gy from 11 Gy at the three timepoints post-TBI, using the CTR mice as the global baseline in LEfSe. The taxa which met cutoff LDA > |2| were plotted on a cladogram [48,49,50,51,52,53]. Further, Phylogenetic Investigation of Communities by Reconstruction of Unobserved States (PICRUSt2) was used to predict the MetaCyc pathway abundances using the sequencing-derived taxonomic ASVs to generate the functional profile: a list of networks that are potentially enriched by microbial metabolites.

Metabolomics analysis: In the untargeted metabolomics, a moderated *t*-test *p* < 0.05 identified significantly different peaks at individual timepoints, and the CTR value was used as the global baseline. The differentially expressed mass spectroscopy peaks were annotated using the CEU Mass Mediator 3.0 database (ceumass.eps.uspceu.es, accessed on 1 June 2023). Annotated metabolites were seeded into Ingenuity Pathway Analysis (IPA, QIAGEN, Germantown, MD, USA) to curate the networks with a cutoff z score > |1.5|.

Prism v.8 (GraphPad, San Diego, CA, USA) was used to analyze the liver tissue Mouse LBP ELISA data. A pairwise comparison analysis was conducted using a *t*-test with the significance cutoff of *p* < 0.05. 

## 3. Results

### 3.1. Animal Health Status Following Lethal Doses of Ionizing Radiation

For CD2F1 male mice, the 9.5 Gy TBI was ~LD50/30; i.e., 50% mortality was recorded in 30 days post-TBI [54], and mice start to display clinical symptoms of pain and distress around 12 days post-TBI. To note, in the current study, the mice were found to be perfectly healthy during the collection period, which was conducted until d9 post-TBI (Figure 1). On the other hand, the 11 Gy TBI was ~LD100/30; i.e., 100% mortality was recorded in 30 days post-TBI, and mice start to display the clinical symptoms of pain starting from d7-d8 post-11 Gy TBI [54]. The clinical symptoms of pain in this strain of mice included the inability to remain upright, unresponsiveness, and decreased or labored breathing. Following the pre-determined criteria, 1 out of 18 mice exposed to 11 Gy was euthanized on day 8 due to a high pain score. This mouse was precluded from the present study. 

### 3.2. Descending Colon Contents’ Bacterial Diversity Showed Dose-Dependent Longitudinal Alterations

**Figure 2:**
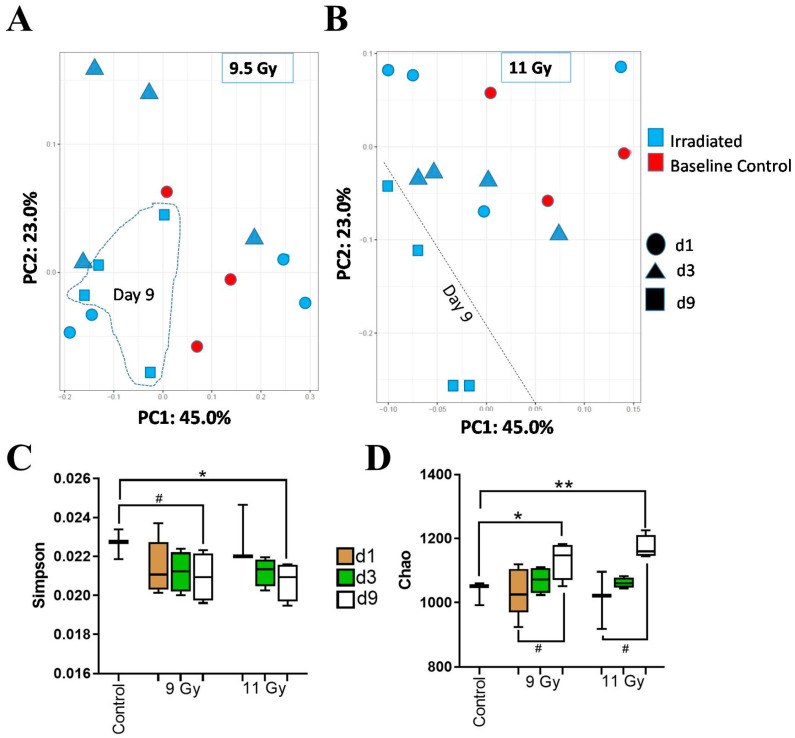
Diversity analysis of descending colon microbiota. (**A**) PCoA plot: Beta diversity of samples exposed to 9.5 Gy TBI using the Bray–Curtis algorithm. Here, the baseline control and irradiated samples are color coded by red and blue, respectively. The timepoints are shape-coded as follows, oval: day 1; triangle: day 3, and square: day 9. The day 9 samples are clustered separately. (**B**) PCoA plot: Beta diversity of samples exposed to 11 Gy TBI using the Bray–Curtis algorithm. Here, the baseline and irradiated samples are color-coded by red and blue, respectively. The timepoints are shape-coded as follows, oval: day 1; triangle: day 3 and square: day 9. The day 9 samples are clustered separately, and the separation is shown by a dotted line. (**C**) Alpha diversity of the entire cohort using Simpson’s index: a box and whisker plot. The timepoints are color-coded, brown: day 1, green: day 3, and white: day 9. The two-way ANOVA with post hoc analysis measured the significance: * *p* < 0.05. # 0.1 < *p* < 0.05. To note, 0.1 < *p* < 0.05 indicates a trend in the diversity profile. (**D**) Alpha diversity of entire cohort using Chao1 index: A box and whisker plot. The timepoints are color-coded, brown: day 1, green: day 3, and white: day 9. The two-way ANOVA with post hoc analysis measured the significance; ** *p* < 0.01, * *p* < 0.05.

Figure S1A depicted that the overall microbial composition of the DCCs was consistent across all the experimental conditions. A PCoA plot using the Bray–Curtis algorithm (Figure S1B) failed to suggest any trend in the bacterial beta diversity among the entire sample set. In support of this trend, a 2-way PERMANOVA analysis using the Bray–Curtis algorithm found TSI (*p* = 0.041) as the sole significant co-factor in explaining the beta-diversity alterations; the remaining factors, RD and the cumulative effects of RD and TSI (e.g., RD × TSI) did not shift the beta diversity. To understand the effects of TSI, additional PCoA plots were made with a separate plot for each of the two radiation doses. Figure 2A displays the PCoA plot depicting the diversity that caused 9.5 Gy TBI, where PC1 and PC2 explained 45% and 23% of total variance, respectively; the samples collected at d9 post-TBI showed a trend by clustering together. This trend was more prominent in the PCoA plot in Figure 2B, where the samples collected at d9 post-11 Gy TBI clustered distinctly apart from rest of the samples. Figure S2A,B present the PCoA plot generated by the Euclidian algorithm for beta diversity, and its clustering pattern is similar to that displayed by the Bray–Curtis algorithm.

Alpha diversity profiles of the bacteria of the DCCs are presented in Figure 2C,D. The two-way ANOVA of alpha diversity measured by Simpson’s index (Figure 2C) found TSI as a significant factor (*p* < 0.05), while RD and their cumulative effects (RD × TSI) emerged as insignificant in explaining the alpha diversity profile. Likewise, the two-way ANOVA of alpha diversity measured by the Chao1 Index found TSI as a significant factor (*p* < 0.01), and its interaction with RD was a moderately significant factor (0.1 < *p* < 0.05), while the exclusive impact of RD emerged as insignificant in explaining the bacterial alpha diversity profile. None of the co-factors, RD and TSI, emerged as significant in explaining the alpha diversity measured by Shannon’s index (Figure S2C).

Furthermore, Simpson’s index at d9 was moderately decreased (0.1 < *p* < 0.05) by 9.5 Gy TBI and significantly decreased (*p* < 0.05) by 11 Gy TBI in comparison to the CTR. Contrastingly, the Chao1 index at d9 was significantly increased by both 9.5 Gy (*p* < 0.05) and 11 Gy (*p* < 0.01) TBI in comparison to the CTR. In fact, the Chao1 index moderately increased from d1 to d9 in both 9.5 Gy and 11 Gy TBI (0.1 < *p* < 0.05).

### 3.3. Shifts in Bacterial Abundances Depended on Radiation Dose and Time since Irradiation

The abundance profile of the bacteria of the DCC at the phylum level is presented in Figure S3; Table S1 lists the detailed information. On average, *Firmicutes* and *Bacteroidetes* together aggregated ~72% of the total phylum-level abundance. *Proteobacteria* was the third most abundant phylum, aggregating around 8% of total abundance on average. The remaining 20% of the abundance profile was populated by a combination of 21 phyla and unclassified entities. 

**Figure 3:**
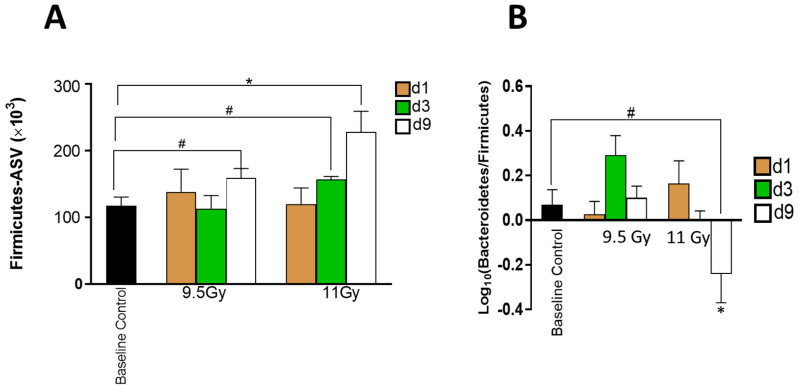
Significantly abundant phyla. (**A**) Abundance of *Firmicutes*. The bar graphs depict the average abundances expressed in ASV ± standard error of mean (SEM) and are plotted against the Y-axis. The timepoints are color-coded, brown: day 1, green: day 3, and white: day 9. The two-way ANOVA with post hoc analysis measured the significance; * *p* < 0.05. # 0.1 < *p* < 0.05. To note, 0.1 < *p* < 0.05 indicates a trend in the abundance profile. (**B**) The log_10_ transformed ratio of *Bacteroidetes* and *Firmicutes*. The Y-axis plots the average log_10_ transformed ratio of abundances of *Bacteroidetes* and *Firmicutes* (*Bacteroidetes*/*Firmicutes*) ± SEM. The two-way ANOVA with post hoc analysis measured the significance; * *p* < 0.05. # 0.1 < *p* < 0.05.

The multiple comparison analysis found that the total abundance of *Firmicutes* significantly varied due to RD (*p* < 0.05) and TSI (*p* < 0.05) but not by their interactive model (Figure 3A). In fact, among the samples exposed to 11 Gy TBI, the abundance profile of *Firmicutes* increased significantly (*p* < 0.001) from d1 to d9 post-11 Gy TBI as measured by the linear regression model.

The log-transformed abundance ratio of *Bacteroidetes* and *Firmicutes* (Figure 3B) significantly shifted due to RD (*p* < 0.05) and its cumulative effect with TSI (e.g., RD × TSI: *p* < 0.05). The exclusive impact of TSI on the relative abundance of *Bacteroidetes* and *Firmicutes* was moderately significant (0.1 < *p* < 0.05). In concurrence with the longitudinal increment of *Firmicutes* post-11 Gy TBI, the log-transformed ratio of *Bacteroidetes* and *Firmicutes* depicted a moderately significant decrement from d1 to d9 post-11 Gy TBI (0.1 < *p* < 0.07).

**Figure 4:**
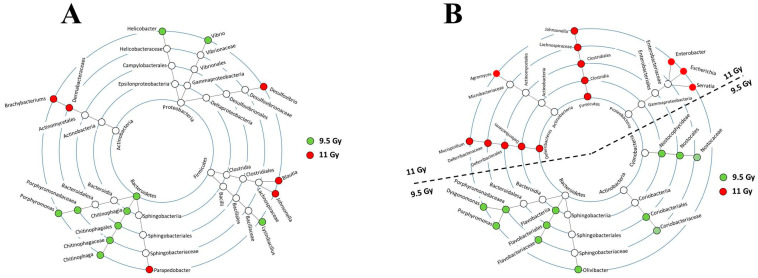
Taxonomic cladogram. Each of the concentric circles represent phylogenetic ranks. From the innermost circle to the outermost circle, they represented Phylum, Class, Order, Family, and Genus, respectively. Each node on the circles represents an individual taxon, and the taxa of one phylogenetic branch are connected by a solid black line. The nodes shifted by 9.5 Gy and 11 Gy TBI are color-coded green and red, respectively; unperturbed nodes are white. (**A**) Cladogram displaying the bacterial abundance profile at day 3 post-9.5 Gy and 11 Gy TBI. (**B**) Cladogram displaying the bacterial abundance profile at day 9 post-9.5 Gy and 11 Gy TBI.

The LEfSe cladogram depicts the taxa that were deferentially abundant due to 9.5 Gy and 11 Gy TBI. Table S2 lists the taxa that are presented in the cladograms. No taxa met the significance cutoff at d1 after either of the radiation doses, 9.5 Gy and 11 Gy TBI. Figure 4A,B plot the cladograms associated with d3 and d9 post-TBI, respectively. Three days after 9.5 Gy TBI, there was potentially a shift in every rank of an entire phylogenetic branch starting from the phylum *Bacteroidetes* to class *Chitinophagia* to order *Chitiniphagales* to family *Chitinophagaceae* to genus *Chitinophaga*. A RD-dependent scenario emerged at d9 post-TBI (Figure 4B) since 9.5 Gy and 11 Gy TBI perturbed a nearly mutually exclusive set of taxa. For instance, 9.5 Gy TBI shifted the taxonomic nodes linked to the phyla *Bacteroidetes*, *Firmicutes* and *Cyanobacteria*. On the other hand, 11 Gy TBI shifted the taxonomic nodes linked to the phyla *Deferribacteres* and *Proteobacteria.* Only the phylum *Actinobacteria* was perturbed by both radiation doses. Notably, at d9 post-11 Gy TBI, there was potentially a shift in every rank of the entire phylogenetic branches stemming from the phyla *Deferribacteres* and *Firmicutes*.

### 3.4. Bionetworks Potentially Linked to Bacterial Shifted with Time since Irradiation

Functional analysis of the bacterial composition was computed using PICRUSt2 followed by LDA-based feature selection. The networks’ activation and inhibition profiles were estimated based on the CTR level (Table S3). To note, in any given condition, if a network was significantly more enriched in comparison to the CTR, this network was considered activated. Alternatively, if a network was significantly less enriched in comparison to the CTR, this network was considered inhibited. Table 1 captures the overall networks’ regulation profile across the RDs and TSI. For instance, at d1 post-9.5 Gy TBI, the majority of the networks were linked to activated amino acid metabolism; subsequently, the networks linked to tryptophan and thiamin became inhibited at d9 post-9.5 Gy TBI. In concurrence, the bioenergy-related networks such as the TCA cycles emerged activated at d9 post-9.5 Gy TBI. On the other hand, the temporal trend in the functional network profile linked to 11 Gy TBI showed that the majority of the networks that were perturbed at d1 post-TBI were linked to amino acid metabolism. With increasing delay since TBI, several bioenergy related networks linked to molecular degradation and fermentation became activated, and this temporal trend operated in sync with the activated biosynthesis of lipids, amino acids, and energy cycle intermediates. Overall, most of these network families, such as amino acid metabolism, biosynthesis, bioenergy, immune functions, and lipid metabolism became activated at d9 post-11 Gy TBI.

### 3.5. Descending Colon Contents’ Metabolite Expression Profile Shifted with Time since Irradiation

**Figure 5:**
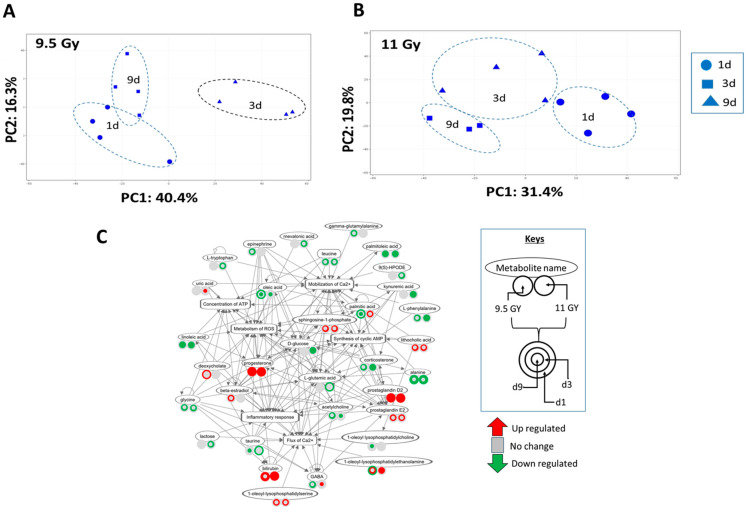
Descending colon metabolite analysis. (**A**) The PCA of the descending colon metabolite profile was perturbed by 9.5 Gy TBI. The timepoints are shape-coded as follows, circle: day 1, square: day 3, and triangle: day 9. All three timepoints are clustered separately and are encompassed by a dotted line. (**B**) The PCA of the descending colon metabolite profile associated with the cohort exposed to 11 Gy TBI. Timepoints are shape-coded as follows, oval: day 1, triangle: day 3, and square: day 9. All three timepoints are clustered separately and are encompassed by a dotted line. (**C**) The metabolite network linked to the flux of calcium ion (Ca2+). The oval and rectangular-shaped nodes represent the metabolites and biofunctions, respectively, and the edges represent the relationship between two nodes. As per the key, each metabolite node is connected to two circles, representing the metabolite’s expression levels in 9.5 Gy and 11 Gy TBI. Furthermore, each circle encompasses three concentric circles representing d1, d3, and d9 by the outer, middle, and inner circles, respectively.

The overall metabolite expression profile showed a dominating temporal bias, as the sample clustering pattern in the Principal Component Analysis (PCA) (Figure 5A,B) nearly mirrored the beta diversity profile of the DCC microbes (Figure 2A,B). For instance, the metabolite expression profiles exposed to either 9.5 Gy (Figure 5A) or 11 Gy (Figure 5B) TBI were distinctly clustered by d1, d3, and d9 post-TBI. The PC1 and PC2 of the PCA plot (Figure 5A) linked to 9.5 Gy TBI explained 40.4% and 16.3% of total variance, respectively; whereas the PC1 and PC2 linked to 11 Gy TBI (Figure 5A) explained 31.4% and 19.8% of total variance, respectively. In this context, the PCoA plots of 16S rRNA gene sequencing data showed distinct separation of the d9 post-TBI samples from the rest of the timepoints, but there was no apparent separation between d1 and d3 post-TBI (Figure 2A,B).

### 3.6. Differential Expression Analysis Identified Putative Early and Time-Independent Metabolite Markers of Irradiation

The metabolites that were identified by differential expression analysis are listed in Table S4, and a corresponding hierarchical clustering plot is presented in Figure S4A. All expression values were computed based on the baseline CTR values. Briefly, there were 40 upregulated and 52 downregulated metabolites at d1 post-9.5 Gy TBI. Likewise, there were 90 upregulated and 116 downregulated metabolites at d3 post-9.5 Gy TBI. In addition, there were 67 upregulated and 57 downregulated metabolites at d9 post-9.5 Gy TBI. There were 21 upregulated and 19 downregulated metabolites at d1 post-11 Gy TBI. Likewise, there were 92 upregulated and 123 downregulated metabolites at d3 post-11 Gy TBI, and there were 55 upregulated and 82 downregulated metabolites at d9 post-11 Gy TBI. A Venn diagram (Figure S4B) identified six metabolites which remained consistently upregulated across all RD and TSI. These metabolites are described in Table 2. Furthermore, there were 12 metabolites that were expressed in at least five of the six study variables (e.g., 2 RD × 3 TSI). Eight of these twelve metabolites showed a longitudinally consistent regulation profile; hence, they became the primary interest for the current study. Four out of these eight metabolites of primary interest were consistently upregulated across all RD and TSI; in contrast, the remaining four metabolites were consistently downregulated across all RD and TSI. These eight metabolites are described in Table 2. In addition, Table 2 includes seven potentially RD-independent early markers; four of the potential markers were upregulated and three were downregulated in both RDs, 9.5 Gy and 11 Gy. 

### 3.7. Regulation Profile of Metabolite-Enriched Networks Shifted Due to Radiation Dose and Time since Irradiation

The functional networks enriched by all differentially expressed metabolites of the DCCs are listed in Table S5, and its overview is presented in Table 1. A rather cohesive regulation pattern emerged at d9 post-11 Gy TBI, since most of the overarching network families (e.g., biosynthesis, bioenergy, immune functions, and lipid metabolism) became primarily inhibited. 

Systems analysis identified the networks linked to calcium ion flux and cyclic AMP, respectively, that were perturbed by all six experimental variables (e.g., 2 RD × 3 TSI). A network linked to calcium ion influx is presented in Figure 5C. This network significantly co-perturbed the four subnetworks of inflammatory response, concentration of ATP, synthesis of cyclic AMP, and metabolism of reactive oxygen species (ROS). The temporal regulation profiles of these subnetworks are presented in Table 3.

### 3.8. LBP Loads in the Liver Samples Were Exclusively Increased by 11 Gy Irradiation

LBP levels in the liver tissue were significantly increased at both d3 and d9 post-11 Gy TBI in comparison to CTR and also to liver tissue exposed to 9.5 Gy TBI (Figure S5). The LBP levels in mice exposed to 9.5 Gy TBI remained unchanged from the CTR level. 

## 4. Discussion

The bidirectional association between the host and its resident microbes is defined as a meta-organism that potentially plays a key role in combat against any foreign stimuli or stress exposure [63]. The purpose of the present work is to understand the longitudinal shift in the bacterial population colonized in the DCC, and in this context, the degree of perturbations that is endured by the meta-organism exposed to lethal radiation. The current hypothesis-driven pilot study is limited by a small sample size; nevertheless, multi-omics analysis of fecal samples collected over time was conducted to enhance the statistical power of the current results. Since the present metagenomics assay measured the load of 16S rRNA, any inference beyond the bacterial family level could have low statistical confidence. In addition, the present analysis precluded all microorganisms except the bacterial kingdom [64]. Further, this study lacks a time-dependent control group, since we assumed that there would be an insignificant change in the bacterial compositions of the DCCs within one week of the lifetime of a healthy young mouse. Indeed, none of the available citations that reported the impacts of age on the microbial ecosystem examined such a short duration in a young cohort [65,66]. 

Diversity calculations estimated the impacts of TBI on the bacterial ecosystem. The beta diversity estimates the shift in the trans-community bacterial profile [67]. In particular, the Bray–Curtis algorithm dissimilarity metrics [68,69] found a trend in the abundance profile at d9 post-TBI that shifted from the earlier timepoints, which could be due to the abundance-based dissimilarity; and the given dissimilarity was most prominent due to 11 Gy TBI. A similar picture emerged from the diversity plot calculated by the Euclidian distance, which is considered a logical routine to operate on a continuous data landscape [70]. Similar results were observed in the alpha diversity estimation that measures the shift in the evenness and richness of the microbial profile within a community [67]. The TBI-induced bacterial profile displayed significant changes in Simpson and Chao1 indices but not in the Shannon diversity estimation [71]. Shannon diversity quantifies both evenness and richness within a community; on the other hand, Chao1 estimates the richness within a community, and Simpson diversity measures the dominant index within a community. Together, the alpha diversity metrics suggested that the temporal reduction in the dominance profile was concurrent with increased richness within this bacterial community of the DCCs. Hence, the alpha and beta diversity metrics revealed a dose-dependent alteration of the bacterial community as 11 Gy TBI caused the most prominent shift in the bacterial ecosystem. Since the mice exposed to lethal 11 Gy TBI (~LD100/30 dose) become moribund following the irradiation, the longitudinal diversity could not be probed beyond d9 post-TBI. 

There was an increment of *Firmicutes*’ abundance, particularly among the moribund mice exposed to 11 Gy TBI. Typically, *Firmicutes* along with *Bacteroidetes* encompass more than 90% of total bacterial community [9]; their abundance ratio (*Bacteroidetes*/*Firmicutes*) significantly decreased over time among the mice exposed to 11 Gy TBI and this change was primarily driven by the increased abundance of *Firmicutes*. Indeed, the reduction in the *Bacteroidetes* and *Firmicutes* ratio has been linked to irradiation [3]; contrasting evidence showed that rectal irradiation caused a pro-inflammatory dysbiosis that was concurrent with a decreased *Firmicutes’* abundance in the rodents’ intestinal flora [15]. 

A decreased *Bacteroidetes*/*Firmicutes* ratio has been linked to several somatic and psychological disorders [72,73,74,75], such as obesity [76,77], and obesity has long been associated with irradiation [78,79]. Indeed, a review reported that the change in this *Bacteroidetes* and *Firmicutes* ratio depends on both RD and TSI [80]. However, a certain analysis contested the applicability of the *Bacteroidetes*/*Firmicutes* ratio as an obesity marker [81], and its counter-argument was built upon the fact that the metabolites originated from these bacterial phyla. For instance, acetate, an obesogenic factor, is one of the major metabolites that is produced by *Bacteroidetes* via metabolizing small chain fatty acids (SCFAs). Acetate promotes fat storage by triggering the brain signals to secrete insulin and ghrelin [82]. In addition, a direct association between the *Bacteroidetes* and inflammation was highlighted by one study that reported an increased abundance of *Bacteroidetes* among the patients with inflammatory bowel diseases (IBS) [83]. On the other hand, emerging knowledge suggested that *Firmicutes* can more effectively harvest energy [84,85]; by metabolizing SCFAs, *Firmicutes* primarily produces butyrate that facilitates bioenergy production and suppresses inflammation [86]. A high abundance of *Firmicutes* was observed in those cohorts that need a high energy intake to survive, such as the human populations living in high altitudes [85] and mice exposed to cold stress [87]. Thus, a delayed increase in *Firmicutes* abundance in mice exposed to lethal 11 Gy TBI could be attributed to the fact that a high amount of energy becomes warranted to survive in extremely stressful conditions. 

*Bacteroidetes* that are typically associated with inflammation [88] remained abundant with 9.5 Gy TBI across the TSI. The LefSe cladogram data showed that the bacterial abundance profile of the DCCs became increasingly distinct with higher radiation doses. In addition to the anti-inflammatory *Firmicutes* [88], the other most enriched phylum linked to 11 Gy TBI was *Deferribacteres*, which is a pro-inflammatory anaerobic bacteria [89]. In tandem, several pro-inflammatory metabolites, namely stercobilin, mesobilirubinogen, and prostaglandin-c2, were consistently elevated across all timepoints post-TBI. Previous radiation studies associated the inflammation inside a gut lumen to intestinal permeability [90]. In support of this, we also reported an elevated load of LBP in the liver, particularly by 11 Gy TBI. Lipopolysaccharide (LPS) is the primary constituent of the outer membrane of Gram-negative bacteria, and this protein is known to cause chronic inflammation in the mouse. An increased expression of LBP is likely to suggest a potential risk of intestinal permeability [91], which can allow the bacteria to escape through the damaged gut lumen, enter the bloodstream, and trigger inflammation. 

A balanced load of intestinal calcium ions benefits various cellular functions, including cell survival and growth, supporting the healthy neuronal synapse, bone formation, and the immune response. Hence, a significantly activated calcium influx network across both RD and TSI could be an indication of dysbiosis caused by Ca+2 overload [91]. An elevated load of Ca+2 can promote intestinal permeability by triggering the apoptosis of epithelium cells and impairing the tight junction proteins, which typically safeguard the intestinal impermeability [92]. In concurrence, there was evidence of consistent activation of AMP production and a potential inhibition of ATP production among the mice exposed to TBI that supported a similar observation [93]. An overloaded AMP molecule along with a depleted ATP concentration is a signature of an energy-deprived condition [94], which has been marked as a potential comorbidity of TBI [95], and current observations found the same outcome.

The host–microbiome communication grid is built upon exchanging metabolites as the key information hub [21,96], and a system deconvolution of metabolite-enriched bionetworks can throw light on the bidirectional relationship between the host and microbes [22]. Hence, two sets of functional networks that were linked to the bacteria and metabolites of the DCCs were contrasted (Table 1). A divergence between these sets of functional networks’ regulation profiles became apparent at d9 post-11 Gy TBI. For instance, the cluster of bacteria-specific biofunctions linked to bioenergy, biosynthesis, and lipid metabolism were activated, but the same network cluster enriched by the metabolites of the DCCs was largely inhibited. Since the lipid metabolism is a key contributor to bioenergy production, an overall activation of these network clusters in TBI-exposed mice potentially suggested a pro-survival environment for the bacterial community after lethal radiation. However, the same clusters of networks, namely lipid metabolism and bioenergy production, were found inhibited when they were enriched by the gut metabolites. It potentially implies that an anti-survival environment is likely to exclusively affect the mice exposed to lethal radiation. Together, we can postulate that the symbiotic relationship between the mouse and its bacteria of the DCCs was likely disrupted at this late stage of 11 Gy TBI. Bacteria colonized in the DCCs potentially withdrew from the symbiotic relationship with the host to create their own pro-survival environment as the mice became increasingly moribund. 

## 5. Conclusions

Our observations supported the hypothesis [9,97] that ARS is associated with the shift in the microbial ecosystem. The shift in the bacterial diversity of the DCCs was found to be dependent on both RD and the TSI. The radiation dose of 11 Gy (LD100/30) caused maximum diversity at day 9-post TBI. Indeed, at this timepoint, the networks enriched by the metabolite profile of the DCCs showed a highly divergent regulatory trend from the networks linked to the bacterial community to the DCCs. We viewed these data in the context of a hypothesis that the metabolite-enriched networks of the DCCs potentially mirror the mouse–microbiome relationship, since both the mouse and bacteria supplement the metabolite profile of the DCCs. Therefore, a sign of divergence between these two networks suggested a disrupted state of the mouse–gut bacterial symbiotic relationship [22]. We presumed that as time progressed after a high dose of TBI, the host and its resident microbes together gradually entered an energy-expensive and resource-limiting condition, when the bacteria of the DCCs developed an opportunistic pro-survival condition exclusively for themselves at the expense of the host or mouse. Our results supported previous observations reporting that the germ-free mice were more irradiation-resistant than the wild-type mice, which was likely because the germ-free mice were not obligated to share their limited resources with their resident microbes during the energy-expensive condition triggered by TBI [13,14]. This observation potentially underscored the importance of critically probing the gut microbial ecosystem, and the current knowledge product could help with improving the chance to survive lethal radiation exposure. 

## Figures and Tables

**Table 1:** Integrative analysis of the networks associated with the bacteria colonized in the descending colon contents (labeled as “Metagenome”) and the metabolites (labeled as “Metabolome”). Each cell includes the number of activated networks and the number of inhibited networks, respectively, separated by a forward slash (“/”). The cells are colored red, blue and yellow and show the number of activated networks that were more, less, or equal to number of inhibited networks, respectively. The gray-colored cells have no enriched networks.

	9.5 Gy						11 Gy					
	Day 1		Day 3		Day 9		Day 1		Day 3		Day 9	
Biofunctions’ Superfamily	Metagenome	Metabolome	Metagenome	Metabolome	Metagenome	Metabolome	Metagenome	Metabolome	Metagenome	Metabolome	Metagenome	Metabolome
Amino acid metabolism	4/1	0/0	0/0	3/2	1/2	1/0	3/2	0/0	3/1	4/0	3/2	0/0
Biosynthesis	0/2	1/1	0/1	0/1	4/3	0/0	0/1	0/0	1/8	1/0	7/1	1/2
Bioenergy	0/0	1/0	0/0	1/1	2/0	1/0	2/0	1/0	1/0	3/1	4/0	2/4
Immune functions	0/0	0/0	0/0	7/1	0/0	0/3	0/0	0/1	0/0	2/0	1/0	0/3
Lipid metabolism	1/0	0/4	0/0	6/1	0/0	2/4	1/1	0/0	2/2	0/1	2/0	0/9

**Table 2:** Consistently regulated metabolites across the time and dose profile. There are three subgroups in these tables. The metabolites which were consistently upregulated across the timepoints are clustered together. There were no metabolites that were significantly downregulated across the timepoints. In addition, there were two subgroups, which were significantly up- or downregulated across all timepoints, except one. Furthermore, a set of potentially early markers was identified. These markers were either up- or downregulated at d1 post-TBI.

Metabolite ID	Metabolite Name	Functional Relevance
All data points are consistently upregulated		
HMDB0240259	Stercobilin/L-urobilin	A pro-inflammatory microbial metabolite, which is generated via the reduction in bilirubin by intestinal microbiota
HMDB0001830	Progesterone	Cancer-related metabolite and gut microbes metabolize and regulate the bioavailability of progesterone [55]
HMDB0001898	Mesobilirubinogen	Closely related to stercobilin, as they have the same parent compound, namely urobilinogen
HMDB0006059	20-Carboxy-leukotriene B4	An oxidized metabolite of leukotriene B4 (LTB4), which is released from polymorphonuclear granulocytes of severely burned patients
HMDB0062552	Goralatide	Protector of hematopoietic progenitors
HMDB0060095	Prostaglandin-c2	Associated with inflammation [56]
*All but one data points are consistently upregulated*		
HMDB0013200	5-Hydroxytryptophol glucuronide (GTOL)	A biomarker of alcohol load in body fluid [57]
HMDB0031039	Heptadecanal	Linked to smoking [58]
HMDB0011563	1-pentadecanoylglycerol	--
HMDB0000054	Bilirubin	Its elevation is linked to several diseases and disorders [59]
*All but one data points are consistently downregulated*		
HMDB0011538	2-Linoleoylglycerol	--
HMDB0060987	2-Hydroxymethylolanzapine	Derivative of olanzapine, an atypical antipsychotic agent
HMDB0000988	S-Adenosylmethioninamine or dadomet	Linked to cancer [60] and psychotic deficiencies [61]
HMDB0000860	Phenylpropionylglycine	Associated with bacteria-driven metabolism [62]
*Early markers:* Consistently upregulated at d1 and d3 post-TBI		
HMDB0001403	Prostaglandin D2 (PGD2)	Associated with inflammation [56]
HMDB0004161	Urobilin	Generated through the degradation of heme
HMDB0062389	A sterol lipid molecule	A member to the class of cholesterols and derivatives
HMDB0006888	5b-Cyprinol sulfate	An intermediate of bile acid biosynthesis
*Early markers:* Consistently downregulated at d1 and d3 post-TBI		
HMDB0062251	Alanine	A member to the class of an alanine or an alanine derivative
HMDB0010727	3-Oxododecanoic acid	An intermediate in fatty acid biosynthesis
HMDB0000860	Phenylpropionylglycine	A fatty acid metabolite that could be a marker of mitochondrial dysfunction

**Table 3:** The regulation profile of the subnetworks coenriched with the network linked to the flux of calcium ions (Figure 5C). The activated and inhibited status of the networks are noted with “Up” (red) and “Down” (blue), respectively.

	9.5 Gy			11 Gy		
Subnetworks	d1	d3	d9	d1	d3	d9
Flux of Ca+2	Up	Up	Up	Up	Up	Up
Synthesis of cAMP	Up	Up	Up	Up	Up	Up
Metabolism of ROS	Down	Up	Down	Up	Up	Down
Inflammatory response	Down	Down	Down	Down	Down	Down
Conc. Of ATP	Down	Down	Down	Down	Down	Down

## Data Availability

All data generated or analyzed during this study are included in this published article and its Supplementary Materials.

## Supplementary Materials

The following supporting information can be downloaded at https://www.mdpi.com/article/10.3390/microorganisms12101995/s1, Figure S1: (A) Overall DCC composition profile, (B) PCoA plot of all experimental conditions; Figure S2: Diversity analysis of descending colon microbiota; Figure S3: (A) The phyla-level abundance profile of the descending colon bacteria, (B) The class level abundance profile of the descending colon bacteria, (C) The order level abundance profile of the descending colon bacteria; Figure S4: (A) Hierarchical cluster using the Euclidian algorithm of the differentially expressed metabolites, (B) Venn diagram of differentially expressed metabolites to curate the common and exclusive candidates across six experimental variables; Figure S5: Abundance of lipopolysaccharide binding protein (LBP) in the liver; Table S1: List of the abundance profile of bacterial phyla, class, order and family in descending colon contents; Table S2: List of those bacterial phyla, class, order, family and genus level taxa that scored LDA ≥ |2|; Table S3: List of functional networks potentially linked to descending colon microbiota. The networks were selected on LDA > |2|; Table S4: List of differentially expressed metabolites in descending colon samples. The metabolites were selected using a two-way ANOVA post hoc *p* < 0.05; Table S5: List of functional networks enriched by metabolites. The networks were selected on z score > |2|.
