# Supplementary material for: Ionizing Radiation Dose Differentially Affects the Host–Microbe Relationship over Time
Source: Microorganisms. 2024 Sep 30;12(10):1995. doi: 10.3390/microorganisms12101995 (PMC11509422; doi:10.3390/microorganisms12101995)
Supplement: Supplementary file 1 [file microorganisms-12-01995-s001.zip › microorganisms-3181536-supplementary.pdf]

Supplementary Tables

Table S1. List of abundance profile of bacterial phyla, class, order and family in descending colon contents. (S1A) Bacterial phyla level OTU across all the experimental conditions. (S1B) Percentage (%) abundance of bacterial phyla. (S1C) Bacterial class level OTU across all the experimental conditions. (S1D) Percentage (%) abundance of bacterial class. (S1E) Bacterial order level OTU across all the experimental conditions. (S1F) Percentage (%) abundance of bacterial order. (S1G) Bacterial family level OTU across all the experimental conditions. (S1H) Percentage (%) abundance of bacterial family.

Table S2. List of those bacterial phyla, class, order, family and genus level taxa that scored LDA ≥ 2.

Table S3. List of functional networks potentially linked to descending colon microbiota. The networks were selected on LDA>|2|.

Table S4. List of differentially expressed metabolites in descending colon samples. The metabolites were selected on ANOVA post-hoc $p<0.05$.

Table S5. List of functional networks enriched by metabolites. The networks were selected on z score >|2|

Supplementary Figures

Figure S1A.

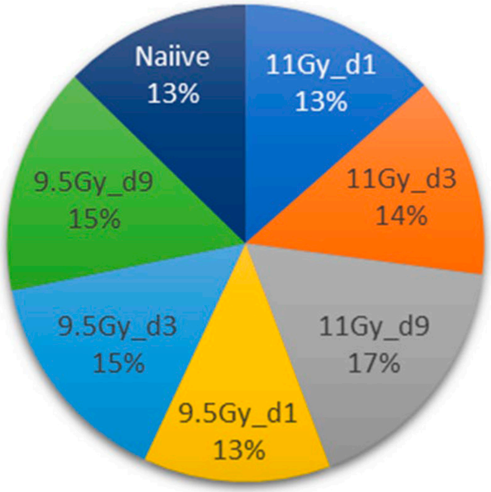

Figure S1B.

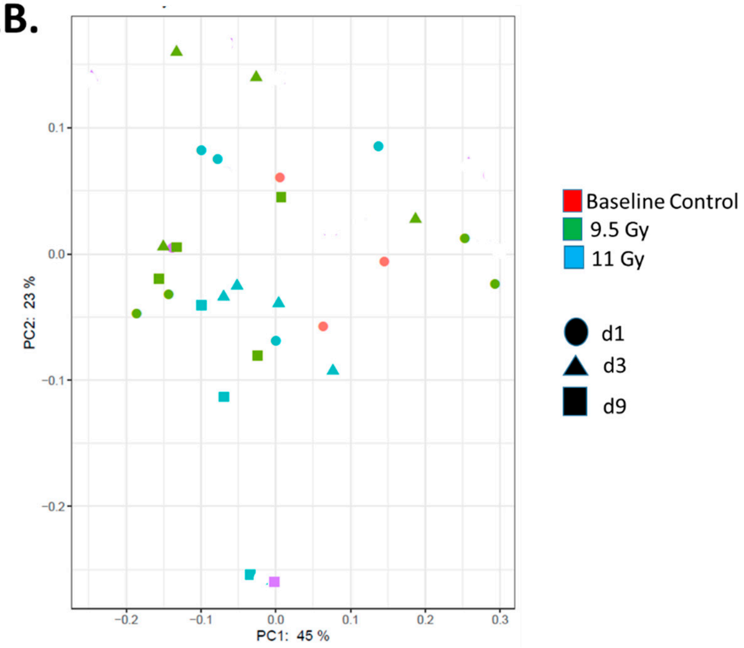

Figure S1. (A) Overall DCC composition profile: A comparative picture of the OTU values across all experimental conditions is depicted in the pie chart. The percentage numbers depict the share of abundance values by each experimental condition. The result indicated a consistent overall composition across all the experimental and control groups. (B) PCoA plot of all experimental conditions: Beta diversity of samples exposed to 9.5 Gy and 11 Gy using Bray-Curtis algorithm. Here the baseline control and irradiated samples are color coded and the timepoints are shape coded. No pattern emerged from this plot.

Figure S2A.

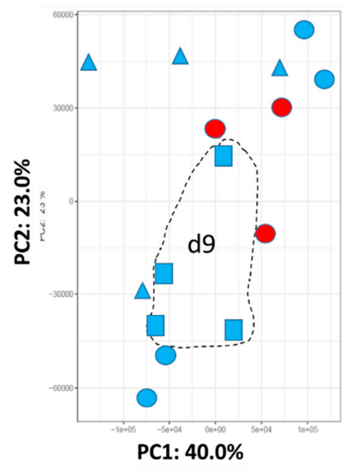

Figure S2B.

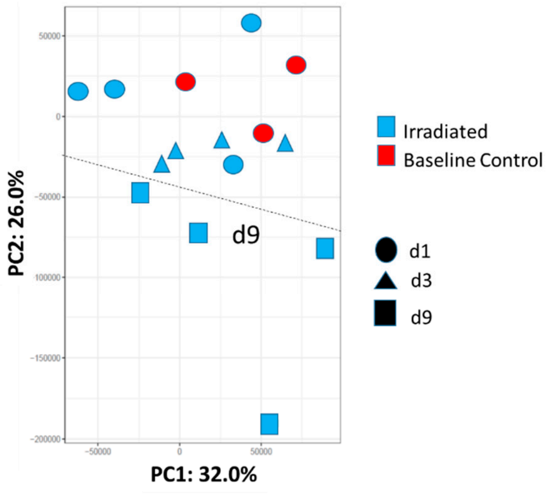

Figure S2C.

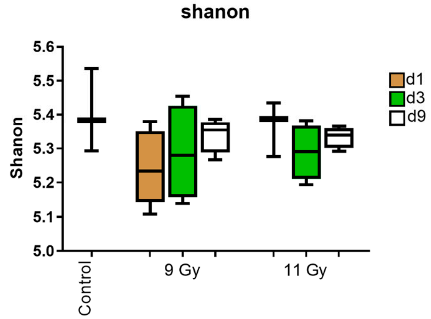

Figure S2. Diversity analysis of descending colon microbiota. (A) PCoA plot of 9.5 Gy TBI: Beta diversity of samples exposed to 9.5 Gy using Euclidian algorithm. Here the baseline control and irradiated samples are color-coded by red and light blue, respectively. The timepoints are shape-coded as follows, circle: day 1, triangle: day 3, and square: day 9. The day 9 samples are clustered separately, so encircled. (B) PCoA plot of 11 Gy TBI: Beta diversity of samples exposed to 11 Gy using Euclidian algorithm. Here the baseline control and irradiate samples are color-coded by red and blue, respectively. The timepoints are shape-coded as follows, circle: day 1, triangle: day 3, and square: day 9. The day 9 samples are clustered separately, so separated by a dotted line. (C) Alpha diversity of entire cohort using Shannon index: A box and whisker plot. The timepoints are color-coded, brown: day 1, green: day 3 and white: day 9. No significant change was observed.

Figure S3A.

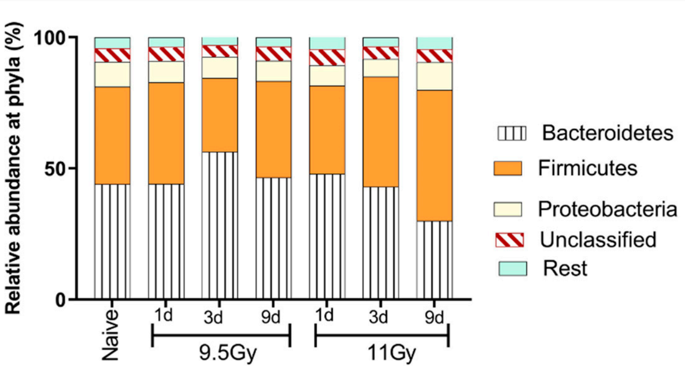

Figure S3B.

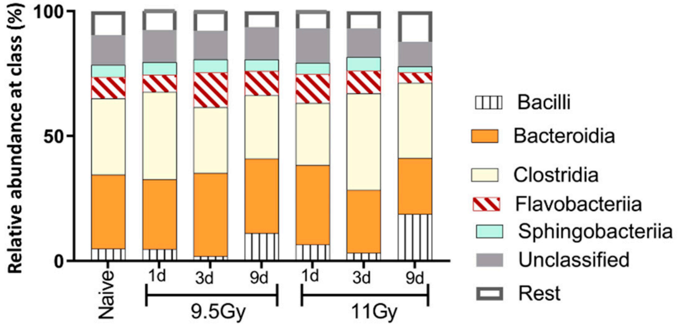

Figure S3C.

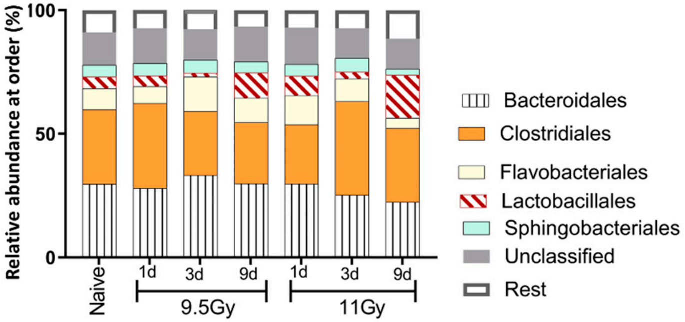

Figure S3D.

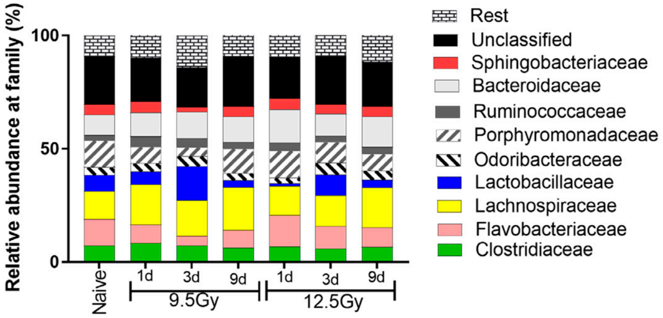

Figure S3. (A) The phyla level abundance profile of the descending colon bacteria. The bar chart presents the percentage abundances of different phyla as per the color codes. Complete list of the abundance profile is in Table S1A-B. Here most abundant four bacterial phyla that encompassed >95% of total abundance were color coded. The rest included *Actinobacteria, Deferribacteres, Cyanobacteria, Tenericutes, Chloroflexi* (noted in descending order of abundance) and so on. (B) The class level abundance profile of the descending colon bacteria. The bar chart presents the percentage abundances of different bacterial classes as per the color codes. Complete list of the abundance profile is in Table S1C-D. Here most abundant six bacterial class that encompassed >90% of total abundance were color coded. The rest included *Gammaproteobacteria, Deltaproteobacteria, Actinobacteria, Deferribacteres, Alphaproteobacteriai* (noted in descending order of abundance) and so on. (C) The order level abundance profile of the descending colon bacteria. The bar chart presents the percentage abundances of different bacterial order as per the color codes. Complete list of the abundance profile is in Table S1E-F. Here most abundant six bacterial orders that encompassed >90% of

total abundance were color coded. The rest included *Enterobacteriales, Actinomycetales, Desulfovibrionales, Deferribacterales, Syntrophobacterales* (noted in descending order of abundance) and so on. (C) The family level abundance profile of the descending colon bacteria. The bar chart presents the percentage abundances of different bacterial family as per the color codes. Complete list of the abundance profile is in Table S1H-G. Here most abundant ten bacterial families that encompassed >90% of total abundance were color coded. The rest included *Desulfovibrionaceae, Prevotellaceae, Deferribacteraceae, Desulfovibrionaceae, Desulfobacteracea and Leuconostocaceae* (noted in descending order of abundance) and so on.

Figure S4A.

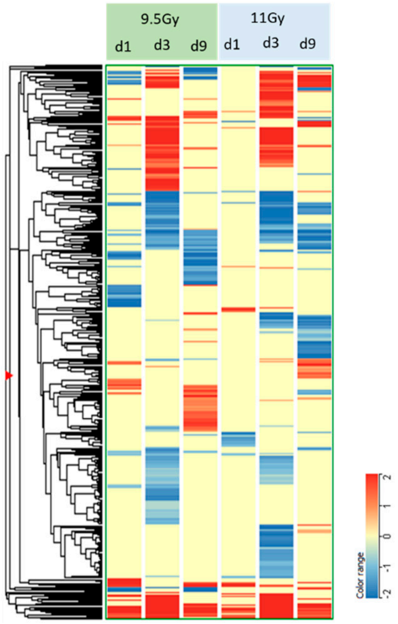

Figure S4B.

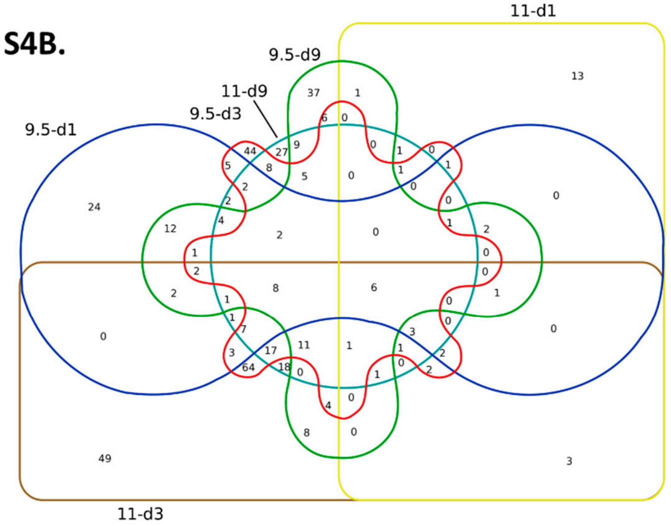

Figure S4. (A) Hierarchical cluster using the Euclidian algorithm of the differentially expressed metabolites. The color key is at right bottom. (B) Venn diagram of differentially expressed metabolites to curate the common and exclusive candidates across six experimental variables (2 radiation doses x 3 timepoints)

Figure S5.

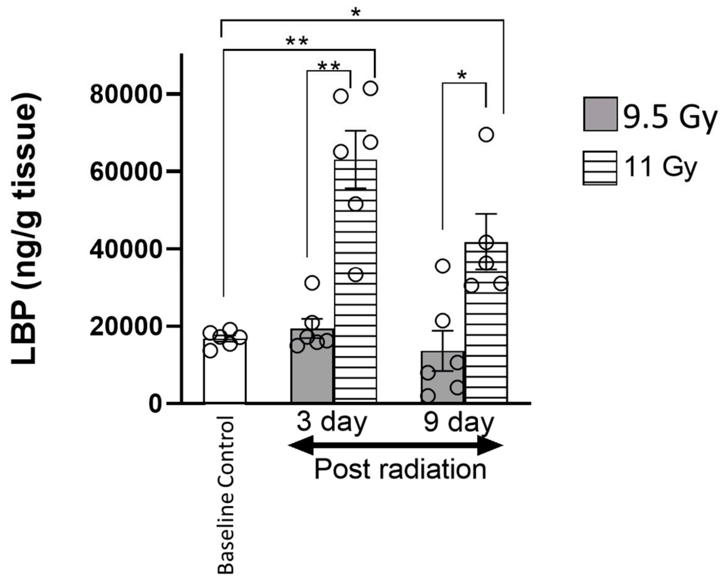

Figure S5. Abundance of lipopolysaccharide binding protein (LBP) in liver. The gray colored and horizontally hashed bars represent 9.5 Gy and 11 Gy irradiation, respectively. ANOVA post-hoc analysis measured the level of significance, *$p < 0.05$. **$p < 0.01$.

Table S1A

Sample ID -->	JJ-10-311	JJ-10-313	JJ-10-314	JJ-10-315	JJ-10-331	JJ-10-333
Study variables: Dose/Time-->	11Gy/day_1	11Gy/day_1	11Gy/day_1	11Gy/day_1	11Gy/day_3	11Gy/day_3
Phylum _						
Phylum_Acidobacteria	5	0	13	0	12	12
Phylum_Actinobacteria	3592	1269	9591	2585	2557	2140
Phylum_Bacteroidetes	147633	141228	221495	243615	181936	115692
Phylum_Caldithrix	6	2	10	11	4	4
Phylum_Chlamydiae	9	17	9	13	4	4
Phylum_Chlorobi	0	0	3	1	2	4
Phylum_Chloroflexi	1395	1130	1827	2107	2506	2220
Phylum_Chrysiogenetes	25	31	23	35	13	7
Phylum_Crenarchaeota	0	0	0	0	0	0
Phylum_Cyanobacteria	2580	3202	3490	2880	1929	1023
Phylum_Deferribacteres	1853	906	1254	3996	5210	3490
Phylum_DNA	2	2	14	9	10	13
Phylum_Euryarchaeota	0	0	1	1	0	0
Phylum_Fibrobacteres	0	2	1	2	0	1
Phylum_Firmicutes	161053	76119	120605	125543	157962	148515
Phylum_Fusobacteria	5	1	3	4	5	1
Phylum_Nitrospirae	78	56	129	111	229	119
Phylum_Proteobacteria	23893	22676	36145	31293	30927	23813
Phylum_Spirochaetes	90	165	463	84	35	37
Phylum_Synergistetes	410	62	340	337	400	435
Phylum_Tenericutes	3360	1479	2997	5933	639	600
Phylum_Thermi	42	61	48	45	39	40
Phylum_Thermodesulfobacteria	23	21	23	31	14	17
Phylum_Thermotogae	2092	82	715	196	3103	449
Phylum_Unclassified	19608	16643	29360	25494	18581	14305
Phylum_Verrucomicrobia	1360	575	1134	710	1269	792

JJ-10-334	JJ-10-335	JJ-10-391	JJ-10-392	JJ-10-393	JJ-10-394	JJ-13-311	JJ-13-312	JJ-13-313
11Gy/day _3	11Gy/day _3	11Gy/day _9	11Gy/day _9	11Gy/day _9	11Gy/day _9	9.5Gy/day _1	9.5Gy/day _1	9.5Gy/day _1
0	1	0	2	8	0	3	8	10
2224	1781	5361	1649	3213	3415	6743	2103	4052
187678	155454	161931	204111	105179	76511	212041	106524	208199
10	5	18	7	10	6	16	1	9
12	8	16	3	14	3	9	3	14
4	1	3	1	1	1	2	3	0
1875	2446	1376	1642	2096	1341	2034	834	1905
20	21	31	11	20	7	21	15	29
0	0	2	0	0	0	0	0	0
2276	2344	3539	997	1776	1114	2665	1325	3710
720	736	1254	7581	13978	3028	3021	567	370
13	3	6	1	11	10	10	3	11
0	0	0	0	0	0	0	0	2
0	0	1	1	0	0	0	0	1
168741	150491	204346	182822	319058	205754	123250	49920	151303
3	4	3	7	4	1	4	1	4
59	210	111	70	238	120	150	36	207
23811	22108	42827	24748	38427	86649	30613	14759	31217
80	17	66	11	120	384	105	111	281
106	280	168	243	773	598	216	77	240
1540	451	1454	215	8210	335	2987	3354	3796
35	33	48	25	88	78	41	14	49
31	12	30	12	27	11	24	10	21
1727	2017	560	176	2797	1407	5095	172	1841
21899	15767	19835	13402	26535	31913	25268	12143	25585
1794	697	5789	671	1297	2816	2407	365	938

JJ-13-314	JJ-13-331	JJ-13-332	JJ-13-333	JJ-13-334	JJ-13-391	JJ-13-392	JJ-13-393	JJ-13-394
9.5Gy/day_1	9.5Gy/day_3	9.5Gy/day_3	9.5Gy/day_3	9.5Gy/day_3	9.5Gy/day_9	9.5Gy/day_9	9.5Gy/day_9	9.5Gy/day_9
6	5	7	43	1	1	11	2	1
4962	1139	3279	1641	2476	1750	5034	2085	3478
240459	113964	220482	319166	254120	154758	241157	173816	235365
15	9	8	3	3	8	10	11	8
5	3	4	7	8	11	19	20	9
0	1	7	1	1	0	0	11	2
2367	1531	1807	2670	2522	1591	2290	1395	2432
22	15	15	15	28	30	26	34	32
0	0	0	0	0	0	0	1	0
2892	1163	1653	2678	2785	3247	4152	3320	4505
1105	1825	884	2659	4919	510	569	639	1365
14	3	6	8	5	13	8	1	10
0	0	0	0	2	2	0	0	1
0	1	0	0	0	1	0	0	1
166752	86830	92546	101413	171334	175230	181133	118142	160960
4	1	4	3	6	6	6	7	8
125	173	235	85	130	39	234	129	235
31812	18550	31546	47868	31207	27969	35460	30286	40059
217	33	25	31	35	49	313	128	61
271	298	195	280	551	228	315	260	391
3684	2808	1515	383	130	565	4870	1526	575
50	29	22	31	50	31	66	31	47
22	4	8	16	19	25	23	24	31
1230	3031	1042	201	680	1944	2104	338	1999
25143	14374	16206	19900	20749	20246	30660	18156	24909
3331	774	461	66	44	1317	1307	1377	1534

NAIVEJJ-10-111	NAIVEJJ-10-112	NAIVEJJ-10-113	NAIVEJJ-10-114
**Naive**	**Naive**	**Naive**	**Naive**
8	2	6	0
2649	3178	2967	2089
109084	178730	232020	128620
11	9	3	4
9	9	9	7
10	1	1	2
1645	1453	2207	1399
12	24	22	15
0	0	0	1
1588	2191	2346	1524
3592	1056	2372	3516
1	5	14	5
0	0	1	1
0	1	4	0
96825	114095	146203	141486
0	3	8	3
281	111	88	56
44951	23279	33434	21706
50	157	245	96
286	148	271	149
909	4384	2884	2387
38	49	39	37
18	27	22	15
344	622	1188	846
15688	18070	25028	15774
1363	838	2006	328

Table S1B

Phylum	Acidobact	Actinobac	Bacteroide	Caldithrix	Chlamydia	Chlorobi	Chloroflex
Study variables: Dose/Time							
naïve	0.001582	0.835126	43.93315	0.002532	0.002637	0.001371	0.474427
9.5Gy-1d	0.001545	0.819156	44.05302	0.002177	0.002949	0.000491	0.426113
9.5Gy-3d	0.003479	0.530288	56.39828	0.001429	0.001367	0.000621	0.529977
9.5Gy-9d	0.000867	0.714066	46.56124	0.00214	0.003412	0.001504	0.445778
11Gy-1d	0.002536	1.357563	47.94081	0.001691	0.003288	0.000845	0.40881
11Gy-3d	0.002236	0.583762	42.98454	0.001543	0.001878	0.000738	0.606906
11Gy-9d	0.001095	0.746643	29.98683	0.002245	0.001971	0.000328	0.353394

Chrysioge	Cyanobact	Deferribac	DNA	Euryarcha	Fibrobacte	Firmicutes	Fusobacte	Nitrospira
0.00538	0.559458	0.86129	0.00116	0.000316	0.000316	37.1783	0.000949	0.047263
0.006811	0.762496	0.744522	0.002106	0	0.000281	38.77221	0.000983	0.033912
0.004536	0.514382	0.639141	0.001367	0.000497	0.000249	28.09085	0.00087	0.038708
0.007056	0.880452	0.1783	0.001851	0.000347	0.000231	36.75094	0.001561	0.03684
0.007421	0.870975	0.376965	0.001691	0.000282	0.000423	33.60814	0.000845	0.024705
0.004092	0.507958	0.681302	0.002616	0	0.000268	41.97487	0.000872	0.041391
0.003778	0.406553	1.414724	0.001533	0	0.000219	49.92842	0.000821	0.029509

Proteobac	Spirochae	Synergiste	Tenericute	Thermi	Thermode	Thermoto	Unclassifi	Verrucomi
9.488111	0.031966	0.061506	0.810228	0.013082	0.00633	0.191163	5.22555	0.266806
8.094462	0.061084	0.100613	0.376193	0.010461	0.005617	0.137474	5.385636	0.199681
8.025521	0.007704	0.082261	0.300465	0.008201	0.00292	0.307797	4.425528	0.083566
7.736572	0.031866	0.069053	0.435831	0.010121	0.005957	0.369265	5.434639	0.320107
7.769823	0.067446	0.076276	0.736083	0.014184	0.006294	0.271381	6.163235	0.28829
6.752577	0.011337	0.081909	0.21668	0.009861	0.004964	0.489443	4.732888	0.305365
10.54712	0.031808	0.09756	0.559189	0.013085	0.00438	0.270452	5.019504	0.578843

crobia

Table S1C

Sample ID -->	JJ-10-311	JJ-10-313	JJ-10-314	JJ-10-315	JJ-10-331	JJ-10-333	JJ-10-334
Study variables: Dose/Time-->	11Gy/day_1	11Gy/day_1	11Gy/day_1	11Gy/day_1	11Gy/day_3	11Gy/day_3	11Gy/day_3
Class _							
Class_Acidimicrobiia	1	0	2	5	10	1	5
Class_Acidobacteria	5	0	13	12	12	0	1
Class_Actinobacteria	3570	1262	9580	2520	2056	2207	1735
Class_Alphaproteobacteria	1423	450	6827	7055	3179	754	480
Class_Anaerolineae	1373	1126	1807	2474	2183	1859	2414
Class_Bacilli	30388	31625	20530	9516	4622	29383	6608
Class_Bacteroidia	98806	82890	133445	96623	59312	112585	109642
Class_Betaproteobacteria	181	76	155	678	732	151	722
Class_Brachyspirae	69	141	226	14	13	51	2
Class_Caldithrixae	6	2	10	4	4	10	5
Class_Chlamydiia	9	17	9	4	4	12	8
Class_Chlorobia	0	0	3	2	4	4	1
Class_Chrysiogenetes	25	31	23	13	7	20	21
Class_Clostridia	127713	42591	97631	146585	141782	137578	142793
Class_Deferribacteres	1853	906	1254	5210	3490	720	736
Class_Dehalococcoidetes	0	0	0	0	0	0	0
Class_Deinococci	42	61	48	39	40	35	33
Class_Deltaproteobacteria	2080	950	1281	3232	6230	2343	3584
Class_Epsilonproteobacteria	47	32	24	55	65	28	35
Class_Erysipelotrichi	159	27	372	731	838	368	326
Class_Fibrobacteria	0	2	1	0	1	0	0
Class_Flavobacteriia	27879	40090	53253	45581	34529	37549	18131
Class_Fusobacteria	5	1	3	5	1	3	4
Class_Gammaproteobacteria	5242	4372	6681	5710	4397	5300	4240
Class_Group_II	2	2	14	10	13	13	3
Class_Halobacteria	0	0	1	0	0	0	0
Class_Holophagae	0	0	0	0	0	0	0
Class_Ktedonobacteria	10	2	12	11	16	16	19
Class_Leptospirae	9	11	8	4	3	7	7
Class_Methylacidiphilae	1	1	2	0	0	0	2
Class_Mollicutes	3360	1479	2997	639	600	1540	451
Class_Nitriliruptoria	8	3	0	4	8	3	1
Class_Nitrospira	78	56	129	229	119	59	210
Class_Nostocophycideae	1943	2363	2407	1426	668	1441	1814
Class_Opitutae	1348	573	1130	1267	790	1789	695
Class_Oscillatoriophycideae	402	451	490	321	248	439	295
Class_Sphingobacteriia	14429	13087	22812	27120	13724	24501	18582
Class_Spirochaetes	12	13	229	17	21	22	8
Class_Synechococcophycideae	2	1	250	0	3	182	0
Class_Synergistia	410	62	340	400	435	106	280

Class_Thermobacula	9	2	7	21	21	0	13
Class_Thermodesulfobacteria	23	21	23	14	17	31	12
Class_Thermoprotei	0	0	0	0	0	0	0
Class_Thermotogae	2092	82	715	3103	449	1727	2017
Class_Unclassified	44089	40867	64947	46730	33085	51816	38952
Class_Verrucomicrobiae	11	1	2	2	2	5	0

JJ-10-335 11Gy/day _3	JJ-10-391 11Gy/day _9	JJ-10-392 11Gy/day _9	JJ-10-393 11Gy/day _9	JJ-10-394 11Gy/day _9	JJ-13-311 9.5Gy/day _1	JJ-13-312 9.5Gy/day _1	JJ-13-313 9.5Gy/day _1	JJ-13-314 9.5Gy/day _1
21	8	15	1	1	11	0	1	2
0	2	8	0	14	2	3	3	0
5329	1610	3158	3388	4132	1471	4138	1820	2570
874	782	1467	792	2070	907	870	251	613
1360	1619	2075	1320	2057	1206	2199	524	2085
117279	71088	128606	33351	26920	3088	4145	19130	6143
117205	156576	86253	46975	138385	56364	178084	42112	167817
304	2616	102	163	788	178	226	101	144
20	2	108	334	214	429	38	18	70
18	7	10	6	22	5	2	2	11
16	3	14	3	12	10	17	3	13
3	1	1	1	2	3	1	1	1
31	11	20	7	25	28	34	10	35
85126	109193	187700	170169	158298	67822	200028	67433	117814
1254	7581	13978	3028	619	862	8838	285	3996
0	0	0	0	1	0	0	0	1
48	25	88	78	62	30	44	13	45
21294	2222	4624	3986	4830	8582	9599	673	3098
123	47	73	55	63	84	80	11	52
293	475	93	346	593	74	179	307	85
1	1	0	0	1	0	0	0	2
19059	26538	7848	20061	38540	16045	42399	7286	50340
3	7	4	1	1	4	8	1	4
3367	8380	14791	71929	8246	5110	7694	2017	5636
6	1	11	10	4	9	16	1	9
0	0	0	0	3	0	1	1	1
0	0	0	0	0	0	0	0	0
11	18	20	14	15	5	29	4	15
6	1	7	1	12	20	14	10	4
4	1	3	2	2	1	0	0	0
1454	215	8210	335	3832	1122	255	149	5933
1	6	5	3	3	2	2	0	3
111	70	238	120	193	125	125	40	111
2674	669	1180	457	2820	1815	2335	1163	1976
5777	669	1282	2755	1860	408	64	501	710
334	221	388	304	607	309	481	202	606
15940	13452	7659	7262	34676	10542	19059	7780	17495
39	8	5	48	68	25	18	4	9
158	1	1	272	90	0	23	0	3
168	243	773	598	438	266	639	90	337

5	5	1	6	4	7	15	3	6
30	12	27	11	32	13	28	7	31
2	0	0	0	0	0	0	0	0
560	176	2797	1407	419	465	736	338	196
48459	33845	50226	45844	64333	32261	64541	19945	57014
8	1	11	59	7	1	0	0	0

JJ-13-331	JJ-13-332	JJ-13-333	JJ-13-334	JJ-13-391	JJ-13-392	JJ-13-393	JJ-13-394	NAIVEJJ-10-111
9.5Gy/day_3	9.5Gy/day_3	9.5Gy/day_3	9.5Gy/day_3	9.5Gy/day_9	9.5Gy/day_9	9.5Gy/day_9	9.5Gy/day_9	Naive
1	6	0	1	3	6	6	8	24
5	7	43	1	1	11	2	1	8
1127	3239	1619	2449	1705	5010	2069	3409	2597
4042	2517	19760	1151	605	1907	445	888	5570
1523	1791	2660	2495	1569	2272	1374	2393	1623
6414	11926	5402	3529	70997	7623	56979	40599	12237
55276	142332	183681	174517	93164	163872	108116	154391	63258
365	936	244	237	316	362	128	259	412
13	15	15	15	20	210	93	24	27
9	8	3	3	8	10	11	8	11
3	4	7	8	11	19	20	9	9
1	7	1	1	0	0	11	2	10
15	15	15	28	30	26	34	32	12
78616	77413	94608	166723	101824	171108	60282	118701	82818
1825	884	2659	4919	510	569	639	1365	3592
1	0	0	0	0	0	1	0	0
29	22	31	50	31	66	31	47	38
1872	11454	2778	6261	5874	3609	8247	8290	25359
44	64	38	59	33	64	47	65	102
1096	2267	752	202	96	824	83	247	794
1	0	0	0	1	0	0	1	0
37394	47093	92448	45776	38991	44269	39843	43161	22889
1	4	3	6	6	6	7	8	0
3113	3178	4594	5308	3108	6990	3266	6475	4248
3	6	8	5	13	8	1	10	1
0	0	0	2	2	0	0	1	0
0	0	0	0	0	0	0	0	0
5	8	7	21	10	13	8	15	11
3	4	8	10	15	16	9	18	9
0	0	0	1	2	1	0	1	1
2808	1515	383	130	565	4870	1526	575	909
1	2	2	3	3	7	2	2	2
173	235	85	130	39	234	129	235	281
769	1203	1822	1967	2490	2576	2313	3579	1035
770	457	62	42	1311	1293	1375	1533	1362
233	313	543	470	419	568	494	448	361
14571	19822	26379	21576	15161	21926	16322	25202	16576
17	6	8	10	14	87	26	19	13
35	0	2	7	1	592	115	6	3
298	195	280	551	228	315	260	391	286

2	8	3	6	11	5	12	23	11
4	8	16	19	25	23	24	31	18
0	0	0	0	0	0	1	0	0
3031	1042	201	680	1944	2104	338	1999	344
31051	41947	57994	52435	48411	66283	47048	63547	32501
4	4	4	1	4	13	2	0	0

NAIVEJJ-10-112	NAIVEJJ-10-113	NAIVEJJ-10-114						
Naive	Naive	Naive						
0	0	0						
2	6	0						
3168	2944	2058						
2023	2194	633						
1447	2183	1371						
26090	4503	21004						
115339	155080	91440						
155	330	67						
131	143	79						
9	3	4						
9	9	7						
1	1	2						
24	22	15						
86129	139333	119104						
1056	2372	3516						
0	1	0						
49	39	37						
1826	3040	4620						
35	64	33						
337	977	168						
1	4	0						
38122	45357	16238						
3	8	3						
5153	8089	5708						
5	14	5						
0	1	1						
0	0	0						
6	14	27						
15	19	13						
0	1	0						
4384	2884	2387						
4	7	4						
111	88	56						
1624	1629	1035						
837	2004	322						
340	373	329						
15960	20159	13622						
11	83	4						
3	104	4						
148	271	149						

0	8	1						
27	22	15						
0	0	1						
622	1188	846						
43235	57816	35132						
1	1	6						

Table S1D

**Class**	Class_Acidi	Class_Acid	Class_Actir	Class_Alph	Class_Anae	Class_Bacil	Class_Bact
Study variables: Dose/Time							
Gy_11_day_1	0.000296	0.001095	1.06227	0.570353	0.421187	6.573019	31.6816
Gy_11_day_3	0.001516	0.001763	0.573762	0.765536	0.60791	3.189294	25.16731
Gy_11_day_9	0.002402	0.000496	0.743225	0.210941	0.346527	18.7309	22.4003
Gy_9.5_day_1	0.001507	0.001518	0.837201	0.288798	0.42415	4.692949	27.95538
Gy_9.5_day_3	0.000555	0.003182	0.537546	1.627161	0.53485	1.90185	33.24167
Gy_9.5_day_9	0.001332	0.000798	0.680459	0.210418	0.434921	11.10305	29.77417
Naive	0.002148	0.00119	0.782784	0.814024	0.47652	4.855885	29.62972

Class_Beta	Class_Brac	Class_Cald	Class_Chla	Class_Chlo	Class_Chry	Class_Clost	Class_Defe	Class_Deha
0.036517	0.03502	0.001794	0.003463	0.000231	0.007914	24.95547	0.508176	5.62E-05
0.159902	0.005111	0.001519	0.001851	0.000753	0.004041	38.64716	0.693081	0
0.180786	0.026478	0.00224	0.001911	0.000332	0.00373	30.16477	1.351391	0
0.085981	0.066292	0.002088	0.00301	0.000649	0.007605	35.00454	0.579295	5.05E-05
0.124187	0.00384	0.001753	0.00133	0.000673	0.004704	26.38758	0.627679	0.000101
0.060675	0.019447	0.002204	0.00353	0.000886	0.00729	25.41839	0.177439	7.11E-05
0.07142	0.025871	0.002108	0.002494	0.001178	0.005181	30.57689	0.802636	5.51E-05

Class_Dein	Class_Delta	Class_Epsil	Class_Erysi	Class_Fibro	Class_Flavo	Class_Fuso	Class_Gam	Class_Grou
0.013904	0.478815	0.010511	0.039727	0.000359	11.58612	0.000832	1.471672	0.001644
0.010016	1.088515	0.012708	0.156788	7.97E-05	9.089718	0.000849	1.319009	0.002645
0.012992	1.773429	0.016325	0.068665	0.000113	4.156587	0.000817	5.699108	0.001518
0.010604	1.803248	0.018446	0.091493	5.05E-05	6.85329	0.001038	1.669773	0.002151
0.008513	1.417054	0.013665	0.311428	0.000101	13.91377	0.000825	1.029143	0.001362
0.009888	1.573666	0.011996	0.065388	0.000116	9.762364	0.001595	1.113024	0.001821
0.011957	2.928869	0.017746	0.162228	0.000292	8.552839	0.000891	1.641745	0.001611

Class_Halo	Class_Holo	Class_Kted	Class_Lept	Class_Met	Class_Moll	Class_Nitril	Class_Nitro	Class_Nost
0.000114	0	0.002406	0.002335	0.000278	0.874374	0.000993	0.024292	0.604956
0	0	0.004253	0.0014	0.000141	0.211643	0.001134	0.041886	0.355405
0	0	0.003436	0.000785	0.000543	0.505203	0.000817	0.028753	0.270918
0.000342	0	0.003259	0.005081	0.00022	0.360449	0.000481	0.036162	0.634225
0.000102	0	0.002463	0.001482	5.08E-05	0.41233	0.000488	0.044201	0.350068
0.000181	0	0.002632	0.003328	0.00023	0.413626	0.000783	0.035438	0.6377
0.000133	0	0.004296	0.003945	0.000145	0.74136	0.001164	0.042337	0.379807

Class_Opit	Class_Oscil	Class_Sphi	Class_Spiro	Class_Syne	Class_Syne	Class_Ther	Class_Ther	Class_Ther
0.250837	0.132209	4.51852	0.015865	0.014943	0.072315	0.001542	0.006613	0
0.297523	0.08671	5.544064	0.004607	0.011212	0.085325	0.003878	0.004928	0
0.586911	0.068015	2.457502	0.005755	0.025272	0.096085	0.000972	0.004306	0.000111
0.218162	0.118776	5.007229	0.007816	0.005594	0.096085	0.002157	0.005461	0
0.114029	0.095749	5.227608	0.003036	0.004005	0.085354	0.001196	0.00271	0
0.325446	0.113286	4.526364	0.008007	0.037585	0.069008	0.003007	0.006059	7.11E-05
0.31759	0.102965	4.804052	0.006842	0.006531	0.062794	0.001504	0.005933	7.81E-05

Class_Ther	Class_Uncl	Class_Verrucomicrobiae
0.202015	13.8124	0.000955
0.47241	11.37206	0.000584
0.259364	9.784687	0.004578
0.159279	12.93765	0.000473
0.421992	11.53769	0.000926
0.356507	13.02476	0.001036
0.206999	11.94264	0.000596

Table S1E

Sample ID -->	JJ-10-311	JJ-10-313	JJ-10-314	JJ-10-315	JJ-10-331	JJ-10-333
Study variables: Dose/Time-->	11Gy/day _1	11Gy/day _1	11Gy/day _1	11Gy/day _1	11Gy/day _3	11Gy/day _3
Order _						
Order_(Microviridae)	2	2	14	10	13	13
Order_Acholeplasmatales	499	244	420	327	254	181
Order_Acidimicrobiales	1	0	2	5	10	1
Order_Acidobacteriales	5	0	13	12	12	0
Order_Actinomycetales	3487	1252	9436	2487	2021	2188
Order_Aeromonadales	0	0	0	0	0	1
Order_Alteromonadales	153	165	281	144	102	189
Order_Anaerolineales	2	0	5	1	0	0
Order_Anaeroplasmatales	2650	1120	2393	219	196	1203
Order_Bacillales	490	206	446	460	480	482
Order_Bacteroidales	98806	82890	133445	96623	59312	112585
Order_Bdellovibrionales	27	17	34	49	34	183
Order_Bifidobacteriales	70	7	140	21	20	10
Order_Borreliales	8	10	224	14	19	19
Order_Brachyspirales	69	141	226	14	13	51
Order_Burkholderiales	165	69	137	612	676	139
Order_Caldilineales	1371	1126	1802	2473	2183	1859
Order_Caldithrixales	6	2	10	4	4	10
Order_Campylobacterales	47	32	24	55	65	28
Order_Caulobacterales	45	34	58	46	61	80
Order_Cerasicoccales	5	2	4	5	2	2
Order_Chlamydiales	9	17	9	4	4	12
Order_Chlorobiales	0	0	3	2	4	4
Order_Chromatiales	22	18	21	46	46	26
Order_Chroococcales	396	444	483	309	243	434
Order_Chrysiogenales	25	31	23	13	7	20
Order_Clostridiales	126139	41997	95973	144431	138186	135989
Order_Coriobacteriales	690	308	851	1643	3183	935
Order_Deferribacterales	1853	906	1254	5210	3490	720
Order_Dehalococcoidales	0	0	0	0	0	0
Order_Deinococcales	17	34	33	17	16	12
Order_Desulfobacterales	0	0	0	0	0	0
Order_Desulfovibrionales	1591	460	993	2986	5911	1888
Order_Desulfurococcales	0	0	0	0	0	0
Order_Desulfuromonadales	47	41	12	34	32	20
Order_Enterobacteriales	1531	845	1193	1653	1266	1443
Order_Entomoplasmatales	70	26	69	43	98	78
Order_Erysipelotrichales	159	27	372	731	838	368
Order_Euzebyales	8	3	0	4	8	3
Order_Exiguobacterales	1	3	5	3	1	4

Order_Fibrobacterales	0	2	1	0	1	0
Order_Flavobacteriales	27879	40090	53253	45581	34529	37549
Order_Fusobacteriales	5	1	3	5	1	3
Order_Gallionellales	0	0	0	0	0	0
Order_Gemellales	819	54	80	158	354	82
Order_Halanaerobiales	43	20	31	29	20	59
Order_Halobacteriales	0	0	1	0	0	0
Order_Holophagales	0	0	0	0	0	0
Order_Hydrogenophilales	3	2	1	4	1	0
Order_Kiloniellales	3	4	7	11	3	2
Order_Lactobacillales	27700	31141	19770	8425	3523	26956
Order_Legionellales	1050	1481	2858	174	234	1022
Order_Leptospirales	9	11	8	4	3	7
Order_Methylacidiphilales	1	1	2	0	0	0
Order_Methylophilales	2	0	0	15	1	1
Order_Mycoplasmatales	40	14	22	22	17	46
Order_Myxococcales	5	4	1	10	12	4
Order_Natranaerobiales	240	21	55	50	36	67
Order_Neisseriales	0	0	0	0	1	1
Order_Nitrospirales	78	56	129	229	119	59
Order_Nostocales	1942	2345	2388	1423	663	1428
Order_Oceanospirillales	27	17	38	61	37	29
Order_Oscillatoriales	1	0	0	0	0	0
Order_Pasteurellales	730	564	648	507	471	549
Order_Pelagicoccales	1333	563	1112	1256	786	1780
Order_Pseudanabaenales	2	1	250	0	3	182
Order_Pseudomonadales	540	321	465	306	267	1188
Order_Puniceicoccales	2	5	6	1	0	3
Order_Rhizobiales	45	34	71	118	57	57
Order_Rhodobacterales	83	39	139	113	67	52
Order_Rhodocyclales	7	1	12	5	12	6
Order_Rhodospirillales	1030	71	6294	6484	2769	351
Order_Rickettsiales	77	150	107	93	56	81
Order_Sphingobacteriales	14429	13087	22812	27120	13724	24501
Order_Sphingomonadales	75	70	56	43	49	65
Order_Spirochaetales	2	3	3	3	2	3
Order_Stigonematales	1	18	19	2	4	11
Order_Sulfolobales	0	0	0	0	0	0
Order_Synergistales	410	62	340	400	435	106
Order_Syntrophobacterales	249	308	101	94	167	151
Order_Thermales	24	27	15	22	24	23
Order_Thermicanales	16	3	14	19	19	8
Order_Thermoanaerobacterales	81	30	134	74	41	48
Order_Thermobaculales	9	2	7	21	21	0
Order_Thermodesulfobacteriales	23	21	23	14	17	31
Order_Thermogemmatisporales	10	2	12	11	16	16
Order_Thermoproteales	0	0	0	0	0	0

Order_Thermotogales	2092	82	715	3103	449	1727
Order_Thiohalorhabdales	54	16	25	85	100	53
Order_Thiotrichales	180	114	228	423	378	258
Order_Turicibacterales	0	0	0	0	0	0
Order_Unclassified	47250	42325	66950	50083	35338	54861
Order_Verrucomicrobiales	11	1	2	2	2	5
Order_Vibrionales	18	12	19	21	24	24
Order_Xanthomonadales	18	54	58	52	70	23

JJ-10-334	JJ-10-335	JJ-10-391	JJ-10-392	JJ-10-393	JJ-10-394	JJ-13-311	JJ-13-312	JJ-13-313
11Gy/day _3	11Gy/day _3	11Gy/day _9	11Gy/day _9	11Gy/day _9	11Gy/day _9	9.5Gy/day _1	9.5Gy/day _1	9.5Gy/day _1
3	6	1	11	10	4	9	16	1
176	429	45	969	82	611	241	84	55
5	21	8	15	1	1	11	0	1
1	0	2	8	0	14	2	3	3
1712	5315	1585	3107	3353	4030	1448	4125	1808
1	3	0	0	1	0	0	0	0
164	228	109	201	260	266	225	248	105
1	0	0	4	5	4	4	0	0
92	854	7	6982	0	2943	729	110	6
502	1149	821	1922	409	387	283	359	271
109642	117205	156576	86253	46975	138385	56364	178084	42112
19	307	74	68	27	123	21	31	12
20	10	24	18	8	83	8	11	10
4	37	5	4	47	63	18	16	2
2	20	2	108	334	214	429	38	18
671	257	2464	50	130	751	142	198	90
2413	1360	1619	2071	1315	2053	1202	2199	524
5	18	7	10	6	22	5	2	2
35	123	47	73	55	63	84	80	11
33	123	39	107	65	261	50	56	22
2	1	2	0	4	5	3	2	3
8	16	3	14	3	12	10	17	3
1	3	1	1	1	2	3	1	1
43	30	39	117	137	30	26	64	4
286	302	217	379	301	595	302	470	201
21	31	11	20	7	25	28	34	10
140895	83755	107517	186120	169092	153906	66448	198556	66526
1434	847	1085	848	601	3608	505	1060	779
736	1254	7581	13978	3028	619	862	8838	285
0	0	0	0	0	1	0	0	0
9	36	11	56	44	29	17	14	6
0	0	0	0	0	0	1	0	0
3459	550	2027	2774	1315	4295	1247	9147	567
0	0	0	0	0	0	0	0	0
6	238	22	272	286	30	87	41	4
1568	838	3335	4728	60281	2254	841	1548	740
110	79	146	47	198	139	68	20	68
326	293	475	93	346	593	74	179	307
1	1	6	5	3	3	2	2	0
2	2	30	25	19	14	2	2	2

0	1	1	0	0	1	0	0	0
18131	19059	26538	7848	20061	38540	16045	42399	7286
4	3	7	4	1	1	4	8	1
0	0	0	1	0	0	0	0	0
84	41	53	104	176	91	32	99	47
19	109	46	136	30	46	275	30	24
0	0	0	0	0	3	0	1	1
0	0	0	0	0	0	0	0	0
2	1	1	4	1	1	5	8	1
1	1	0	2	7	11	4	1	0
5570	105578	68693	118999	32327	25886	2483	3483	17710
152	183	30	1499	3817	2497	1752	379	242
7	6	1	7	1	12	20	14	10
2	4	1	3	2	2	1	0	0
4	2	6	2	5	3	1	4	1
11	48	15	39	39	27	33	21	7
6	108	9	11	6	4	73	16	0
67	36	68	75	151	97	15	16	17
0	1	3	4	18	0	0	1	0
210	111	70	238	120	193	125	125	40
1812	2667	668	1175	453	2810	1806	2329	1159
26	20	46	87	71	33	27	104	13
0	3	0	0	1	0	0	0	0
703	533	1154	1043	1272	1584	578	757	386
683	5765	658	1265	2740	1845	397	45	492
0	158	1	1	272	90	0	23	0
676	149	140	460	169	740	160	243	201
3	4	7	9	8	3	1	8	5
36	52	62	74	97	67	46	72	19
41	59	88	137	57	67	75	98	12
7	35	14	38	5	6	11	6	4
163	244	232	577	329	1419	519	320	104
59	51	212	77	69	100	86	154	26
18582	15940	13452	7659	7262	34676	10542	19059	7780
43	290	99	370	90	53	66	64	45
3	2	3	1	1	4	6	2	2
2	7	1	5	4	8	8	6	4
0	2	0	0	0	0	0	0	0
280	168	243	773	598	438	266	639	90
50	13812	34	1239	2066	217	3791	229	40
24	12	14	32	34	33	13	30	7
6	12	22	55	68	9	11	33	2
42	103	32	130	42	96	26	48	12
13	5	5	1	6	4	7	15	3
12	30	12	27	11	32	13	28	7
19	11	18	20	14	15	5	29	4
0	0	0	0	0	0	0	0	0

2017	560	176	2797	1407	419	465	736	338
100	8	45	57	42	57	117	84	16
327	411	281	354	243	214	250	522	93
0	0	0	0	0	2	0	0	0
40416	66423	39066	64787	52314	66285	37701	68921	21397
0	8	1	11	59	7	1	0	0
21	18	40	65	100	36	24	67	16
43	180	97	120	87	92	24	76	17

JJ-13-314	JJ-13-331	JJ-13-332	JJ-13-333	JJ-13-334	JJ-13-391	JJ-13-392	JJ-13-393	JJ-13-394
9.5Gy/day _1	9.5Gy/day _3	9.5Gy/day _3	9.5Gy/day _3	9.5Gy/day _3	9.5Gy/day _9	9.5Gy/day _9	9.5Gy/day _9	9.5Gy/day _9
9	3	6	8	5	13	8	1	10
816	1082	657	191	59	87	1035	268	150
2	1	6	0	1	3	6	6	8
0	5	7	43	1	1	11	2	1
2542	1113	3219	1606	2433	1684	4986	2049	3367
0	0	1	0	0	0	0	0	0
215	91	170	127	181	201	231	198	260
1	1	3	0	1	1	5	1	0
4920	708	600	51	5	19	3327	1015	227
286	851	408	299	486	820	475	745	879
167817	55276	142332	183681	174517	93164	163872	108116	154391
14	18	136	32	108	42	198	169	44
14	11	16	10	13	15	22	14	33
7	13	2	5	7	9	82	24	14
70	13	15	15	15	20	210	93	24
112	328	866	212	208	285	311	108	237
2084	1522	1788	2660	2494	1568	2267	1373	2393
11	9	8	3	3	8	10	11	8
52	44	64	38	59	33	64	47	65
41	31	51	22	32	91	59	31	68
6	2	1	6	7	1	4	4	4
13	3	4	7	8	11	19	20	9
1	1	7	1	1	0	0	11	2
47	32	37	28	51	46	31	16	79
598	227	311	529	456	407	555	482	431
35	15	15	15	28	30	26	34	32
116393	76170	75386	93391	165356	98348	168487	59052	116881
945	1887	1681	654	824	2910	1986	908	1365
3996	1825	884	2659	4919	510	569	639	1365
1	1	0	0	0	0	0	1	0
13	16	11	12	10	10	26	17	19
0	0	0	0	0	0	0	0	0
2608	1434	2879	2347	5834	4819	2887	869	5725
0	0	0	0	0	0	0	0	0
50	20	65	38	31	48	35	114	96
1517	593	1085	1694	1432	955	1691	475	2569
47	218	179	75	36	87	204	145	86
85	1096	2267	752	202	96	824	83	247
3	1	2	2	3	3	7	2	2
2	1	2	7	1	40	3	1	5

2	1	0	0	0	1	0	0	1
50340	37394	47093	92448	45776	38991	44269	39843	43161
4	1	4	3	6	6	6	7	8
0	0	0	0	0	0	0	0	0
235	81	82	37	70	52	321	23	104
17	49	38	30	24	63	14	44	72
1	0	0	0	2	2	0	0	1
0	0	0	0	0	0	0	0	0
6	3	4	7	6	1	5	2	0
6	2	1	18	2	1	3	1	3
5439	5072	10861	4750	2756	69012	6573	51418	36018
755	248	161	225	137	317	2583	977	321
4	3	4	8	10	15	16	9	18
0	0	0	0	1	2	1	0	1
10	1	2	1	4	8	12	0	5
19	21	13	9	24	27	36	28	40
2	5	45	5	6	10	6	28	24
22	40	22	29	26	29	29	26	21
1	0	0	0	1	0	1	0	0
111	173	235	85	130	39	234	129	235
1957	764	1202	1816	1965	2487	2560	2306	3572
39	46	22	21	50	23	41	19	39
1	0	0	0	0	0	0	0	0
535	411	488	579	692	329	980	308	1288
698	766	446	41	25	1288	1276	1359	1513
3	35	0	2	7	1	592	115	6
336	242	121	152	186	373	419	354	262
3	0	4	5	3	6	6	6	5
33	40	43	104	59	66	53	30	72
102	65	62	164	59	44	89	39	94
6	6	10	6	7	5	16	15	11
173	3720	2153	19037	744	227	1426	109	338
154	75	88	173	137	58	113	59	86
17495	14571	19822	26379	21576	15161	21926	16322	25202
40	27	46	29	42	60	63	124	136
2	4	3	3	3	5	4	2	4
19	4	1	6	2	3	15	7	6
0	0	0	0	0	0	0	1	0
337	298	195	280	551	228	315	260	391
229	230	5656	211	202	663	300	4765	1603
32	13	11	19	39	21	40	14	28
20	19	10	9	19	8	6	24	19
74	142	76	16	45	37	111	55	70
6	2	8	3	6	11	5	12	23
31	4	8	16	19	25	23	24	31
15	5	8	7	21	10	13	8	15
0	0	0	0	0	0	0	0	0

196	3031	1042	201	680	1944	2104	338	1999
82	55	51	68	145	139	99	33	199
208	189	171	347	430	204	263	314	415
0	0	0	0	0	24	1	143	0
59794	33851	46390	60511	55214	51081	68136	54728	69394
0	4	4	4	1	4	13	2	0
25	17	20	25	42	32	31	6	26
44	172	60	59	26	43	86	159	42

NAIVEJJ-10-111	NAIVEJJ-10-112	NAIVEJJ-10-113	NAIVEJJ-10-114
Naive	Naive	Naive	Naive
1	5	14	5
213	612	977	266
24	0	0	0
8	2	6	0
2561	3152	2906	2016
0	0	0	0
178	139	207	110
2	2	3	1
483	3553	1536	2016
358	319	595	348
63258	115339	155080	91440
21	84	250	32
26	4	32	11
4	4	75	0
27	131	143	79
385	139	304	52
1621	1445	2180	1370
11	9	3	4
102	35	64	33
92	76	82	60
2	1	3	1
9	9	9	7
10	1	1	2
31	22	65	32
269	335	369	326
12	24	22	15
81646	85211	137612	118067
752	496	934	702
3592	1056	2372	3516
0	0	1	0
17	26	15	19
0	0	0	0
3626	1164	2263	4362
0	0	0	1
92	49	42	27
1157	1160	2250	1061
129	68	167	31
794	337	977	168
2	4	7	4
3	7	3	9

0	1	4	0
22889	38122	45357	16238
0	3	8	3
0	0	0	0
123	74	122	80
25	9	20	25
0	0	1	1
0	0	0	0
1	2	1	4
4	2	4	0
11440	25381	3496	20206
276	1797	2236	1172
9	15	19	13
1	0	1	0
3	1	3	5
45	37	23	16
102	2	5	3
59	25	31	12
1	0	1	0
281	111	88	56
1030	1620	1625	1029
25	17	55	33
0	0	0	0
541	649	1280	581
1351	830	1989	313
3	3	104	4
175	453	464	777
4	2	8	4
457	42	69	63
86	45	108	43
6	5	7	5
4702	1652	1709	307
70	98	76	50
16576	15960	20159	13622
45	53	58	39
9	4	8	3
4	4	4	6
0	0	0	0
286	148	271	149
14577	398	196	123
21	23	24	18
21	6	22	11
53	80	93	38
11	0	8	1
18	27	22	15
11	6	14	27
0	0	0	0

344	622	1188	846
46	62	49	138
220	232	250	342
0	0	0	0
41846	44745	60425	37387
0	1	1	6
13	17	35	22
34	36	78	37

Table S1F

Order	Order_(Mi	Order_Ach	Order_Acid	Order_Acid	Order_Acti	Order_Aer	Order_Alte	Order_Ana
Study variables: Dose/Time								
Gy_11_day	0.001518	0.108252	0.000245	0.00146	1.203946	0	0.056313	0.000568
Gy_11_day	0.002645	0.063618	0.001516	0.001763	0.566182	0.000131	0.039913	0.000132
Gy_11_day	0.001518	0.07764	0.002402	0.000496	0.73648	0.000227	0.044153	0.000492
Gy_9.5_da	0.002151	0.071391	0.001507	0.001518	0.826975	0	0.066823	0.000679
Gy_9.5_da	0.001362	0.166431	0.000555	0.003182	0.533318	6.72E-05	0.036214	0.000354
Gy_9.5_da	0.001821	0.083235	0.001332	0.000798	0.674316	0	0.051898	0.00038
Naive	0.001611	0.13762	0.002148	0.00119	0.773038	0	0.045908	0.000566

Order_Ana	Order_Baci	Order_Bac	Order_Bde	Order_Bifid	Order_Bor	Order_Bra	Order_Bur	Order_Cald
0.565442	0.104689	29.67258	0.007208	0.01806	0.019354	0.04145	0.034184	0.404846
0.108068	0.130901	25.16731	0.018088	0.004894	0.0038	0.005111	0.147073	0.607778
0.38116	0.227153	22.4003	0.026191	0.003266	0.005365	0.026478	0.165033	0.346036
0.241346	0.109012	27.95538	0.01187	0.007097	0.006347	0.066292	0.076946	0.423472
0.114922	0.153388	33.24167	0.018059	0.003352	0.002059	0.00384	0.112654	0.534496
0.248396	0.174839	29.77417	0.026719	0.004762	0.007037	0.019447	0.053612	0.434541
0.540307	0.114915	29.62972	0.024191	0.005237	0.00478	0.025871	0.065251	0.475954

Order_Cald	Order_Cam	Order_Cau	Order_Cer	Order_Chla	Order_Chld	Order_Chr	Order_Chr	Order_Chr
0.001568	0.01012	0.012828	0.001013	0.003643	0.000233	0.005874	0.128926	0.007931
0.001519	0.012708	0.014832	0.000728	0.001851	0.000753	0.011085	0.084639	0.004041
0.00224	0.016325	0.018093	0.00041	0.001911	0.000332	0.017722	0.065395	0.00373
0.002088	0.018446	0.024887	0.001137	0.00301	0.000649	0.008119	0.116688	0.007605
0.001753	0.013665	0.0093	0.000926	0.00133	0.000673	0.009726	0.093593	0.004704
0.002204	0.011996	0.014493	0.000754	0.00353	0.000886	0.009741	0.110136	0.00729
0.002108	0.017746	0.022894	0.000494	0.002494	0.001178	0.010436	0.093919	0.005181

Order_Clos	Order_Cori	Order_Def	Order_Deh	Order_Dei	Order_Des	Order_Des	Order_Des	Order_Des
24.10438	0.166963	0.378266	0	0.00836	0	0.278412	0	0.010318
37.99892	0.511855	0.693081	0	0.003676	0	1.011761	0	0.006265
29.85262	0.185684	1.351391	0	0.007952	0	0.357726	0	0.044701
34.42014	0.403823	0.579295	5.05E-05	0.005001	0.000119	0.865784	0	0.01434
25.8729	0.378954	0.627679	0.000101	0.003471	0	0.753008	0	0.009876
24.88418	0.420066	0.177439	7.11E-05	0.004119	0	0.812013	0	0.01792
30.23025	0.209217	0.802636	5.51E-05	0.005698	0	0.873498	7.81E-05	0.016174

Order_Ent	Order_Ent	Order_Erys	Order_Euz	Order_Exig	Order_Fibr	Order_Flav	Order_Fus	Order_Gall
0.336803	0.014936	0.046603	0.001099	0.000855	0.000328	11.67767	0.00081	0
0.399778	0.0229	0.156788	0.001134	0.000646	7.97E-05	9.089718	0.000849	0
4.089482	0.026883	0.068665	0.000817	0.004158	0.000113	4.156587	0.000817	4.77E-05
0.39218	0.025906	0.091493	0.000481	0.001327	5.05E-05	6.85329	0.001038	0
0.290686	0.039721	0.311428	0.000488	0.000637	0.000101	13.91377	0.000825	0
0.312333	0.030391	0.065388	0.000783	0.003047	0.000116	9.762364	0.001595	0
0.393707	0.028053	0.162228	0.001164	0.001639	0.000292	8.552839	0.000891	0

Order_Gem	Order_Hala	Order_Hal	Order_Hol	Order_Hyd	Order_Kilo	Order_Lact	Order_Leg	Order_Lep
0.086941	0.008797	7.76E-05	0	0.000599	0.001316	7.941499	0.502309	0.002813
0.048766	0.008269	0	0	0.000466	0.001105	2.815321	0.101649	0.0014
0.020859	0.01699	0	0	0.000364	0.000572	17.42243	0.313101	0.000785
0.019754	0.03996	0.000342	0	0.001157	0.001078	4.332273	0.387337	0.005081
0.019136	0.010245	0.000102	0	0.001229	0.001273	1.62225	0.0542	0.001482
0.026153	0.011622	0.000181	0	0.000452	0.000439	10.28934	0.233247	0.003328
0.029292	0.005939	0.000133	0	0.000601	0.000722	4.615835	0.368468	0.003945

Order_Met	Order_Met	Order_Myc	Order_Myx	Order_Nat	Order_Neis	Order_Nitr	Order_Nos	Order_Oce
0.000371	0.000181	0.007075	0.001031	0.028574	0	0.024076	0.654783	0.007519
0.000141	0.001342	0.006253	0.002234	0.014696	0.00014	0.041886	0.353898	0.010272
0.000543	0.00085	0.007737	0.007416	0.018548	0.001501	0.028753	0.269992	0.012161
0.00022	0.000599	0.007273	0.009636	0.009883	4.57E-05	0.036162	0.631793	0.011524
5.08E-05	0.000489	0.004674	0.004087	0.008308	5.08E-05	0.044201	0.349092	0.009736
0.00023	0.001363	0.00758	0.004181	0.006229	4.9E-05	0.035438	0.635859	0.006877
0.000145	0.000896	0.0092	0.009781	0.00972	0.000145	0.042337	0.378383	0.009067

Order_Osc	Order_Past	Order_Pela	Order_Pse	Order_Pse	Order_Pun	Order_Rhiz	Order_Rho	Order_Rho
9.03E-05	0.186941	0.277265	0.0197	0.125104	0.001273	0.013837	0.023171	0.001688
0	0.151267	0.295141	0.011212	0.1593	0.000454	0.017756	0.018297	0.002118
0.000227	0.221805	0.583902	0.025272	0.048404	0.001533	0.0158	0.018272	0.004862
0	0.239477	0.213918	0.005594	0.096704	0.001362	0.014914	0.018543	0.002469
0	0.138647	0.110968	0.004005	0.049738	0.000672	0.015154	0.021971	0.001937
0	0.158427	0.320953	0.037585	0.083348	0.001367	0.012732	0.014876	0.002747
0	0.210939	0.314574	0.006531	0.134438	0.001255	0.052636	0.020239	0.001672

Order_Rho	Order_Rick	Order_Sph	Order_Sph	Order_Spir	Order_Stig	Order_Sulf	Order_Syn	Order_Syn
0.590177	0.03407	4.714313	0.019898	0.00079	0.003822	0	0.071178	0.068957
0.651197	0.019209	5.544064	0.013491	0.000736	0.001246	0	0.085325	0.031702
0.074153	0.022756	2.457502	0.044872	0.00039	0.000926	0.000111	0.096085	0.9548
0.163212	0.026112	5.007229	0.02	0.001299	0.002212	0	0.096085	0.479156
1.513148	0.029148	5.227608	0.009417	0.00091	0.000875	0	0.085354	0.424308
0.109926	0.017955	4.526364	0.022866	0.000868	0.001739	7.11E-05	0.069008	0.47977
0.657522	0.021392	4.804052	0.014074	0.001768	0.001334	0	0.062794	1.353461

Order_The	Order_The	Order_The	Order_The	Order_The	Order_The	Order_The	Order_The	Order_Thic
0.006718	0.002907	0.021473	0.001607	0.006496	0.002085	0	0.254673	0.008823
0.00634	0.003585	0.013661	0.003878	0.004928	0.004253	0	0.47241	0.023425
0.00504	0.008639	0.016293	0.000972	0.004306	0.003436	0	0.259364	0.008259
0.005602	0.003564	0.01188	0.002157	0.005461	0.003259	0	0.159279	0.022986
0.004992	0.004015	0.022595	0.001196	0.00271	0.002463	0	0.421992	0.019781
0.005769	0.003507	0.015388	0.003007	0.006059	0.002632	0	0.356507	0.026528
0.006259	0.004382	0.018579	0.001504	0.005933	0.004296	0	0.206999	0.022046

Order_Thio	Order_Turi	Order_Und	Order_Ver	Order_Vibr	Order_Xanthomonadales
0.048242	0	14.7699	0.001274	0.004605	0.012899
0.09467	0	12.04407	0.000584	0.006127	0.013185
0.070434	0	12.16729	0.004578	0.012402	0.02652
0.07796	0.000101	14.09561	0.000473	0.010063	0.013446
0.069894	0	12.38754	0.000926	0.006455	0.025749
0.070011	0.011753	14.13863	0.001036	0.00536	0.020475
0.076832	0	13.20726	0.000596	0.006031	0.012817

Table S1G

Sample ID -->	JJ-10-311	JJ-10-313	JJ-10-314	JJ-10-315	JJ-10-331	JJ-10-333
Study variables: Dose/Time-->	11Gy/day _1	11Gy/day _1	11Gy/day _1	11Gy/day _1	11Gy/day _3	11Gy/day _3
Family _						
Family_Acetobacteraceae	3	4	9	11	11	9
Family_Acholeplasmataceae	499	244	420	327	254	181
Family_Acidimicrobiaceae	1	0	2	5	10	1
Family_Acidobacteriaceae	5	0	13	12	12	0
Family_Actinomycetaceae	674	209	2675	301	32	18
Family_Actinopolysporaceae	3	0	9	3	3	2
Family_Actinosynnemataceae	8	1	4	43	37	6
Family_Aerococcaceae	21	8	10	4	15	36
Family_Aeromonadaceae	0	0	0	0	0	1
Family_Alcaligenaceae	89	3	32	296	263	21
Family_Alcanivoracaceae	0	0	0	0	0	0
Family_Alteromonadaceae	112	132	181	102	68	139
Family_Aminiphilaceae	4	1	8	10	6	4
Family_Amoebophilaceae	33	61	43	27	16	25
Family_Anaerobrancaceae	240	21	54	48	34	67
Family_Anaerolinaceae	2	0	5	1	0	0
Family_Anaeroplasmataceae	2650	1120	2393	219	196	1203
Family_Anaplasmataceae	16	18	15	22	12	18
Family_Aurantimonadaceae	0	0	0	0	0	2
Family_Bacillaceae	64	74	93	67	96	52
Family_Bacteroidaceae	36621	19896	43191	32842	16390	31355
Family_Bartonellaceae	0	0	1	0	0	0
Family_Bdellovibrionaceae	27	17	34	49	34	183
Family_Beijerinckiaceae	0	0	1	0	0	0
Family_Bifidobacteriaceae	70	7	140	21	20	10
Family_Bogoriellaceae	0	0	0	0	0	0
Family_Borreliaceae	8	10	224	14	19	19
Family_Brachyspiraceae	69	141	226	14	13	51
Family_Bradyrhizobiaceae	19	7	17	38	23	18
Family_Brevibacteriaceae	568	148	549	819	954	1332
Family_Brucellaceae	0	0	0	0	0	0
Family_Burkholderiaceae	3	6	10	9	2	7
Family_Caldicellulosiruptoraceae	14	7	8	28	16	12
Family_Caldilineaceae	1371	1126	1802	2473	2183	1859
Family_Caldithrixaceae	6	2	10	4	4	10
Family_Campylobacteraceae	33	15	8	38	35	15
Family_Carboxydocellaceae	3	1	4	2	5	1
Family_Carnobacteriaceae	3	0	1	1	0	0
Family_Caulobacteraceae	45	34	58	46	61	80
Family_Cellulomonadaceae	1	0	0	0	0	0

Family_Cerasicoccaceae	5	2	4	5	2	2
Family_Chitinophagaceae	108	101	165	149	103	97
Family_Chlorobiaceae	0	0	3	2	4	4
Family_Chromatiaceae	17	8	13	21	21	12
Family_Chroococcaceae	1	0	0	0	2	0
Family_Chrysiogenaceae	25	31	23	13	7	20
Family_Clostridiaceae	38679	13193	26673	34441	35345	17818
Family_Comamonadaceae	63	58	88	250	345	93
Family_Contubernalisaceae	0	0	1	2	2	0
Family_Coprobacillaceae	16	3	33	493	414	29
Family_Coriobacteriaceae	690	308	851	1643	3183	935
Family_Corynebacteriaceae	90	133	74	65	124	210
Family_Coxiellaceae	364	0	331	10	8	22
Family_Cyanobacteriaceae	0	0	0	0	0	0
Family_Cystobacteraceae	0	2	0	3	3	0
Family_Deferribacteraceae	1853	906	1254	5210	3490	720
Family_Dehalobacteriaceae	451	138	280	915	1049	744
Family_Dehalococcoidaceae	0	0	0	0	0	0
Family_Deinococcaceae	17	34	33	17	16	12
Family_Dermabacteraceae	2	2	2	4	2	5
Family_Dermacoccaceae	0	0	0	0	0	0
Family_Desulfobacteraceae	212	273	62	74	157	129
Family_Desulfohalobiaceae	69	62	61	214	337	22
Family_Desulfonatronumaceae	11	5	7	11	12	11
Family_Desulfovibrionaceae	1508	392	923	2758	5554	1853
Family_Desulfurococcaceae	0	0	0	0	0	0
Family_Desulfuromonadaceae	1	0	1	4	1	0
Family_Dethiosulfovibrionaceae	136	29	113	233	212	54
Family_Dietziaceae	0	0	0	0	0	0
Family_Ectothiorhodospiraceae	7	10	7	24	24	15
Family_Enterobacteriaceae	1531	845	1193	1653	1266	1443
Family_Enterococcaceae	258	97	634	147	125	64
Family_Entomoplasmataceae	70	26	69	43	98	78
Family_Erysipelotrichaceae	143	24	339	238	422	339
Family_Erythrobacteraceae	29	10	18	13	14	30
Family_Eubacteriaceae	982	251	730	764	887	1022
Family_Euzebyaceae	8	3	0	4	8	3
Family_Exiguobacteraceae	1	3	5	3	1	4
Family_Ferrimonadaceae	0	0	1	0	0	0
Family_Fibrobacteraceae	0	2	1	0	1	0
Family_Flammeovirgaceae	1	0	0	0	0	1
Family_Flavobacteriaceae	27879	40090	53253	45581	34529	37549
Family_Flexibacteraceae	508	855	620	547	298	729
Family_Francisellaceae	1	1	2	1	0	0
Family_Frankiaceae	0	0	0	0	0	0
Family_Fusobacteriaceae	3	1	2	4	1	1
Family_Gallionellaceae	0	0	0	0	0	0

Family_Gemellaceae	819	54	80	158	354	82
Family_Geobacteraceae	1	1	3	5	1	3
Family_Geodermatophilaceae	0	0	0	0	0	0
Family_Glycomycetaceae	2	1	1	1	2	0
Family_Gomphosphaeriaceae	2	0	1	1	0	0
Family_Gordoniaceae	0	0	0	0	0	0
Family_Hahellaceae	0	0	0	0	0	0
Family_Halanaerobiaceae	42	20	31	29	18	59
Family_Halobacteriaceae	0	0	1	0	0	0
Family_Halobacteroidaceae	1	0	0	0	2	0
Family_Halomonadaceae	16	9	21	42	30	18
Family_Helicobacteraceae	8	11	9	7	15	8
Family_Heliobacteriaceae	247	88	268	180	184	264
Family_Holophagaceae	0	0	0	0	0	0
Family_Hydrogenophilaceae	3	2	1	4	1	0
Family_Hyphomicrobiaceae	18	12	25	40	18	23
Family_Hyphomonadaceae	3	3	43	37	17	5
Family_Idiomarinaceae	1	1	4	1	5	2
Family_Kiloniellaceae	3	4	7	11	3	2
Family_Kineosporiaceae	0	0	0	0	0	0
Family_Lachnospiraceae	64749	23053	47279	76262	65811	95900
Family_Lactobacillaceae	24002	28745	17007	6869	2556	23615
Family_Legionellaceae	685	1480	2525	163	226	1000
Family_Leptospiraceae	9	11	8	4	3	7
Family_Leuconostocaceae	1825	920	866	637	307	1921
Family_Listeriaceae	2	2	0	2	2	0
Family_Litoricolaceae	9	4	14	17	6	10
Family_Methylacidiphilaceae	1	1	2	0	0	0
Family_Methylobacteriaceae	1	14	1	3	5	3
Family_Methylocystaceae	0	0	0	1	3	1
Family_Methylophilaceae	2	0	0	15	1	1
Family_Microbacteriaceae	31	12	14	37	37	13
Family_Micrococcaceae	1	2	1	0	0	0
Family_Microcystaceae	94	136	102	85	34	34
Family_Micromonosporaceae	55	29	69	86	127	77
Family_Microviridae	2	2	14	10	13	13
Family_Moraxellaceae	538	318	463	304	262	1185
Family_Mycobacteriaceae	2	0	4	1	2	0
Family_Mycoplasmataceae	40	14	22	22	17	46
Family_Nannocystaceae	5	2	1	7	8	3
Family_Neisseriaceae	0	0	0	0	1	1
Family_Nitrospinaceae	0	0	0	0	0	0
Family_Nocardiaceae	4	1	3	4	4	5
Family_Nocardioidaceae	10	8	30	53	25	54
Family_Nostocaceae	1942	2345	2388	1423	663	1428
Family_Oceanospirillaceae	0	2	2	2	1	0
Family_Odoribacteraceae	11091	11916	11910	8802	14755	28592

Family_Oxalobacteraceae	4	1	4	7	7	11
Family_Paenibacillaceae	291	66	96	129	96	275
Family_Paraprevotellaceae	4	4	52	118	78	9
Family_Pasteurellaceae	730	564	648	507	471	549
Family_Pelagicoccaceae	1333	563	1112	1256	786	1780
Family_Pelobacteraceae	0	0	0	0	0	0
Family_Peptococcaceae	903	217	1619	1139	822	453
Family_Peptostreptococcaceae	30	6	14	38	25	31
Family_Phormidiaceae	121	121	183	93	84	150
Family_Phyllobacteriaceae	0	0	0	0	0	0
Family_Piscirickettsiaceae	2	4	0	17	9	3
Family_Planococcaceae	8	5	10	17	8	10
Family_Polyangiaceae	0	0	0	0	1	1
Family_Porphyromonadaceae	32739	37270	53102	38020	18025	33246
Family_Prevotellaceae	4489	778	5801	2715	1638	3461
Family_Promicromonosporaceae	0	0	1	0	0	0
Family_Propionibacteriaceae	7	6	10	7	4	22
Family_Pseudanabaenaceae	2	1	250	0	3	182
Family_Pseudoalteromonadaceae	0	2	2	1	1	1
Family_Pseudomonadaceae	2	3	2	2	5	3
Family_Pseudonocardiaceae	0	1	3	3	1	0
Family_Psychromonadaceae	1	0	1	0	0	1
Family_Puniceicoccaceae	2	5	6	1	0	3
Family_Rhabdochlamydiaceae	0	0	0	0	0	0
Family_Rhizobiaceae	4	0	15	27	6	6
Family_Rhodobacteraceae	63	26	82	50	29	39
Family_Rhodocyclaceae	7	1	12	5	12	6
Family_Rhodospirillaceae	991	46	6244	6431	2729	279
Family_Rhodothermaceae	386	56	189	410	1209	190
Family_Rickettsiaceae	61	131	92	70	44	63
Family_Rikenellaceae	209	131	223	209	36	70
Family_Rivulariaceae	1	18	19	2	4	11
Family_Ruminococcaceae	11482	2455	13920	19617	21900	9659
Family_Saccharospirillaceae	0	0	0	0	0	0
Family_Saprospiraceae	4	3	11	6	3	1
Family_Shewanellaceae	36	29	89	37	26	44
Family_Sinobacteraceae	0	8	12	4	6	3
Family_Sphingobacteriaceae	13327	11928	21709	25912	12041	23383
Family_Sphingomonadaceae	46	60	38	30	35	35
Family_Spirochaetaceae	2	3	3	3	2	3
Family_Sporichthyaceae	0	0	0	0	1	0
Family_Sporolactobacillaceae	0	0	0	0	0	0
Family_Staphylococcaceae	12	25	142	50	31	14
Family_Streptococcaceae	464	350	696	274	208	312
Family_Streptomycetaceae	59	44	57	106	124	70
Family_Streptosporangiaceae	1145	325	4012	349	22	11
Family_Sulfobacillaceae	1	4	3	2	3	5

Family_Sulfolobaceae	0	0	0	0	0	0
Family_Symbiobacteriaceae	0	0	1	0	1	1
Family_Synergistaceae	259	30	210	138	205	46
Family_Syntrophaceae	13	4	2	7	3	0
Family_Syntrophobacteraceae	23	31	34	12	4	22
Family_Syntrophomonadaceae	52	3	21	19	12	11
Family_Thermaceae	24	27	15	22	24	23
Family_Thermicanaceae	16	3	14	19	19	8
Family_Thermoactinomycetaceae	8	3	9	26	17	12
Family_Thermoanaerobacteraceae	58	15	124	32	19	23
Family_Thermobaculaceae	9	2	7	21	21	0
Family_Thermodesulfobacteriaceae	23	21	23	14	17	31
Family_Thermodesulfovibrionaceae	78	56	129	229	119	59
Family_Thermogemmatisporaceae	10	2	12	11	16	16
Family_Thermomonosporaceae	13	0	1	7	8	2
Family_Thermoproteaceae	0	0	0	0	0	0
Family_Thermotogaceae	2092	82	715	3103	449	1727
Family_Thermovenabulum	8	7	0	8	6	13
Family_Thiohalorhabdaceae	54	16	25	85	100	53
Family_Thiotrichaceae	175	109	219	382	349	252
Family_Tsukamurellaceae	0	0	0	0	1	0
Family_Turicibacteraceae	0	0	0	0	0	0
Family_Unclassified	71709	59513	94181	76455	57052	82686
Family_Veillonellaceae	78	28	49	53	37	43
Family_Verrucomicrobiaceae	11	1	2	2	2	5
Family_Vibrionaceae	18	12	19	21	24	24
Family_Waddliaceae	0	0	0	0	0	0
Family_Xanthobacteraceae	0	0	5	1	1	0
Family_Xanthomonadaceae	18	46	46	48	64	20
Family_Yaniellaceae	107	24	7	227	218	4

JJ-10-334	JJ-10-335	JJ-10-391	JJ-10-392	JJ-10-393	JJ-10-394	JJ-13-311	JJ-13-312	JJ-13-313
11Gy/day _3	11Gy/day _3	11Gy/day _9	11Gy/day _9	11Gy/day _9	11Gy/day _9	9.5Gy/day _1	9.5Gy/day _1	9.5Gy/day _1
4	8	6	8	12	10	12	4	2
176	429	45	969	82	611	241	84	55
5	21	8	15	1	1	11	0	1
1	0	2	8	0	14	2	3	3
33	1421	19	174	36	662	25	40	178
4	9	1	13	3	7	5	6	2
16	1	9	7	8	8	9	22	0
7	96	64	80	76	25	47	2	18
1	3	0	0	1	0	0	0	0
280	21	1336	0	2	381	40	82	31
0	0	1	1	0	0	0	0	0
111	206	62	142	144	208	179	157	84
2	5	1	14	9	11	6	12	4
18	17	24	31	32	40	137	32	8
67	34	67	74	151	93	13	16	16
1	0	0	4	5	4	4	0	0
92	854	7	6982	0	2943	729	110	6
17	27	44	30	15	20	15	37	4
0	0	0	0	0	0	0	2	0
75	70	90	184	122	103	63	61	45
59531	64787	118113	21129	6840	44443	33590	84016	9653
0	0	0	0	0	0	0	0	0
19	307	74	68	27	123	21	31	12
0	0	0	0	0	0	0	0	0
20	10	24	18	8	83	8	11	10
0	0	0	0	0	0	0	0	0
4	37	5	4	47	63	18	16	2
2	20	2	108	334	214	429	38	18
10	11	13	4	4	31	14	30	7
884	355	912	1356	1343	1004	756	2682	778
0	0	0	0	5	1	0	0	0
12	2	21	11	5	13	10	28	4
7	11	11	22	10	14	5	20	3
2413	1360	1619	2071	1315	2053	1202	2199	524
5	18	7	10	6	22	5	2	2
18	68	16	41	35	25	31	38	5
0	26	7	41	2	7	2	7	5
1	2	1	6	0	1	4	0	1
33	123	39	107	65	261	50	56	22
0	0	1	0	0	0	0	0	0

2	1	2	0	4	5	3	2	3
71	61	164	89	74	107	116	162	31
1	3	1	1	1	2	3	1	1
13	25	25	84	153	17	20	41	3
1	0	0	0	0	0	0	1	1
21	31	11	20	7	25	28	34	10
28064	43389	19334	35242	34214	36139	12922	30631	11493
312	232	899	38	120	308	73	68	50
0	2	0	1	0	4	2	0	1
19	26	237	1	0	38	22	0	3
1434	847	1085	848	601	3608	505	1060	779
68	14	5	377	320	70	31	82	59
4	3	2	11	7	107	18	3	2
1	0	0	0	0	0	1	0	0
1	31	7	3	1	1	24	7	0
736	1254	7581	13978	3028	619	862	8838	285
955	86	324	559	584	488	277	903	94
0	0	0	0	0	1	0	0	0
9	36	11	56	44	29	17	14	6
2	2	7	2	8	3	3	3	0
0	0	0	0	0	0	0	0	0
22	13725	25	1212	2056	183	3733	193	27
186	58	73	32	139	169	63	206	17
6	38	3	31	9	26	22	6	1
3266	453	1949	2706	1161	4099	1162	8930	548
0	0	0	0	0	0	0	0	0
1	16	0	6	5	3	14	2	1
186	96	174	262	307	269	123	160	49
0	0	0	0	0	0	0	0	0
30	5	16	34	16	13	7	24	2
1568	838	3335	4728	60281	2254	841	1548	740
147	2000	2615	427	123	312	738	165	76
110	79	146	47	198	139	68	20	68
307	267	238	92	346	555	52	179	304
18	137	23	149	45	18	34	28	20
1578	565	718	1262	563	1958	314	650	410
1	1	6	5	3	3	2	2	0
2	2	30	25	19	14	2	2	2
1	0	0	0	8	0	0	0	0
0	1	1	0	0	1	0	0	0
0	0	0	0	1	2	0	1	0
18131	19059	26538	7848	20061	38540	16045	42399	7286
440	1077	231	322	258	814	441	498	122
0	0	1	2	0	0	2	0	0
0	0	0	0	0	0	0	0	0
2	2	6	3	1	1	4	7	1
0	0	0	1	0	0	0	0	0

84	41	53	104	176	91	32	99	47
0	27	0	0	4	4	13	0	1
0	0	0	0	0	0	0	0	0
0	1	4	3	6	3	1	2	1
0	0	0	2	0	0	0	2	0
0	0	0	0	0	0	0	0	0
0	0	0	0	0	0	0	0	0
19	105	46	134	30	43	275	27	24
0	0	0	0	0	3	0	1	1
0	4	0	2	0	3	0	3	0
18	8	37	66	37	18	14	83	8
9	8	15	12	13	17	20	26	3
102	66	39	1151	757	532	87	274	86
0	0	0	0	0	0	0	0	0
2	1	1	4	1	1	5	8	1
19	13	11	32	23	24	20	30	8
10	7	10	16	8	2	5	9	2
2	4	3	3	9	3	7	7	0
1	1	0	2	7	11	4	1	0
0	0	0	0	0	0	0	1	0
71120	25611	67710	98700	96059	86970	35367	126049	38342
4272	87703	60710	102837	29558	22607	777	2622	15570
148	180	27	1486	3810	2390	1732	376	240
7	6	1	7	1	12	20	14	10
466	11022	2669	10384	1145	895	326	247	1396
0	7	3	9	0	3	2	4	3
6	11	8	14	9	8	12	12	5
2	4	1	3	2	2	1	0	0
2	1	2	10	8	1	2	1	2
0	0	0	1	1	0	0	0	0
4	2	6	2	5	3	1	4	1
49	35	29	41	52	33	32	36	14
1	0	0	1	0	0	1	0	0
22	15	12	16	7	82	84	138	24
103	65	106	141	405	109	73	213	45
3	6	1	11	10	4	9	16	1
674	142	135	456	157	734	157	233	198
1	0	1	2	5	2	2	3	0
11	48	15	39	39	27	33	21	7
4	77	1	7	4	2	48	8	0
0	1	3	4	18	0	0	1	0
0	0	0	0	0	0	1	0	0
0	2	3	7	3	2	4	4	1
6	6	1	11	6	195	57	278	15
1812	2667	668	1175	453	2810	1806	2329	1159
2	1	0	5	22	4	1	7	0
9666	6029	1467	44494	30940	9039	2231	11526	7975

5	1	0	0	3	5	2	4	2
123	897	189	1350	90	97	64	62	187
3	3	267	2	0	21	4	69	4
703	533	1154	1043	1272	1584	578	757	386
683	5765	658	1265	2740	1845	397	45	492
0	0	2	1	0	0	0	0	0
787	469	154	1774	310	1384	931	548	313
26	14	8	22	40	27	24	24	7
88	119	54	168	164	203	103	158	75
0	0	0	0	0	0	1	0	0
5	1	27	36	16	6	4	28	2
10	30	15	43	18	23	9	11	3
1	0	1	1	1	1	1	1	0
25313	27064	30373	8660	5885	56852	12405	63175	15294
219	43	1005	898	18	3730	401	3770	916
0	0	0	1	0	0	0	0	0
13	3	6	12	76	17	2	8	4
0	158	1	1	272	90	0	23	0
2	1	4	3	18	2	5	3	1
2	7	5	4	12	6	3	10	3
1	438	2	10	11	2	155	9	1
0	1	0	6	2	0	1	2	1
3	4	7	9	8	3	1	8	5
0	0	0	0	0	0	0	0	1
4	16	18	5	6	8	7	4	2
22	39	51	77	29	59	44	49	8
7	35	14	38	5	6	11	6	4
95	214	204	494	236	1362	495	280	80
604	363	299	2680	967	237	62	361	96
41	24	168	45	54	79	71	117	22
88	52	36	11	31	18	53	44	8
2	7	1	5	4	8	8	6	4
28082	8334	11573	30051	21889	16634	10392	11792	3627
0	0	0	0	2	0	0	0	0
1	4	4	2	3	5	9	9	1
47	15	35	40	28	52	31	76	18
7	11	7	18	13	8	8	6	1
17366	14331	12583	4501	5899	33348	9700	17925	7499
25	152	76	221	45	35	32	36	25
3	2	3	1	1	4	6	2	2
0	0	0	0	0	1	0	1	0
0	0	0	0	0	0	0	0	0
45	20	412	28	60	51	28	32	3
265	791	1325	1258	458	637	200	171	57
92	55	94	153	134	156	112	168	55
79	1993	29	42	50	1057	5	27	347
4	0	5	8	8	6	2	5	1

0	2	0	0	0	0	0	0	0
1	2	1	1	0	0	0	2	0
82	61	66	480	265	150	135	456	37
1	5	0	2	2	0	1	1	1
27	76	9	25	7	34	56	35	12
8	20	10	21	7	13	10	8	2
24	12	14	32	34	33	13	30	7
6	12	22	55	68	9	11	33	2
17	9	9	21	9	9	7	29	2
25	87	11	87	26	57	16	20	6
13	5	5	1	6	4	7	15	3
12	30	12	27	11	32	13	28	7
210	111	70	238	120	193	125	125	40
19	11	18	20	14	15	5	29	4
10	2	2	5	3	5	4	5	3
0	0	0	0	0	0	0	0	0
2017	560	176	2797	1407	419	465	736	338
9	4	9	20	5	24	5	5	3
100	8	45	57	42	57	117	84	16
319	407	226	268	218	206	242	458	90
0	0	0	0	4	0	0	0	0
0	0	0	0	0	2	0	0	0
66691	96352	54390	98861	72745	102983	52513	113340	42859
30	36	23	55	24	62	15	15	20
0	8	1	11	59	7	1	0	0
21	18	40	65	100	36	24	67	16
1	1	1	1	0	0	0	0	0
0	7	11	0	0	0	0	1	0
36	169	89	102	74	84	16	70	16
110	3	7	11	73	79	4	154	11

JJ-13-314	JJ-13-331	JJ-13-332	JJ-13-333	JJ-13-334	JJ-13-391	JJ-13-392	JJ-13-393	JJ-13-394
9.5Gy/day _1	9.5Gy/day _3	9.5Gy/day _3	9.5Gy/day _3	9.5Gy/day _3	9.5Gy/day _9	9.5Gy/day _9	9.5Gy/day _9	9.5Gy/day _9
10	7	11	14	12	7	18	6	12
816	1082	657	191	59	87	1035	268	150
2	1	6	0	1	3	6	6	8
0	5	7	43	1	1	11	2	1
252	173	631	30	31	86	725	299	538
1	1	6	2	4	3	6	1	5
11	5	18	8	16	13	7	4	13
11	14	16	11	2	24	15	47	39
0	0	1	0	0	0	0	0	0
19	152	436	105	94	109	98	39	115
0	1	0	0	0	1	0	0	0
151	70	129	88	113	164	163	162	185
5	5	6	13	1	4	8	5	4
39	19	32	27	20	31	48	23	57
21	39	20	29	24	29	29	25	20
1	1	3	0	1	1	5	1	0
4920	708	600	51	5	19	3327	1015	227
29	15	20	43	28	17	26	20	18
0	0	0	0	0	0	1	0	1
57	586	125	54	60	426	160	87	217
44369	13313	80304	71110	85724	22235	59380	24999	69995
0	0	0	0	0	0	0	0	0
14	18	136	32	108	42	198	169	44
1	0	0	0	0	1	1	0	0
14	11	16	10	13	15	22	14	33
0	0	0	0	0	0	0	0	0
7	13	2	5	7	9	82	24	14
70	13	15	15	15	20	210	93	24
14	12	14	28	29	17	14	18	31
1025	288	367	782	1542	688	1317	278	653
0	0	0	0	2	0	0	0	1
12	8	13	7	10	12	12	9	11
18	18	30	8	19	6	26	3	11
2084	1522	1788	2660	2494	1568	2267	1373	2393
11	9	8	3	3	8	10	11	8
29	23	21	14	20	20	32	27	39
1	11	2	0	2	4	9	11	13
1	1	4	1	1	0	1	0	5
41	31	51	22	32	91	59	31	68
1	0	0	1	0	0	0	0	0

6	2	1	6	7	1	4	4	4
144	102	130	288	139	77	144	73	127
1	1	7	1	1	0	0	11	2
21	10	21	12	22	28	11	11	52
0	0	1	1	0	0	0	0	0
35	15	15	15	28	30	26	34	32
23927	26476	23212	20816	27465	20257	39736	19507	25734
73	139	354	74	85	148	173	56	102
0	1	2	0	2	0	0	1	0
4	677	1373	2	0	12	46	9	9
945	1887	1681	654	824	2910	1986	908	1365
210	77	51	115	33	154	230	167	148
5	7	7	6	2	5	349	26	29
0	0	0	0	0	0	0	2	0
1	3	6	1	3	4	1	4	3
3996	1825	884	2659	4919	510	569	639	1365
676	438	519	675	1557	1561	707	211	1886
1	1	0	0	0	0	0	1	0
13	16	11	12	10	10	26	17	19
1	0	2	2	4	2	3	2	7
0	0	0	0	0	0	0	0	0
197	214	5613	186	164	614	250	4696	1552
119	234	441	239	146	61	114	79	130
2	10	20	6	15	6	14	12	24
2487	1188	2414	2102	5671	4750	2757	776	5568
0	0	0	0	0	0	0	0	0
1	2	6	0	2	3	0	7	3
252	110	90	106	186	156	225	167	224
0	0	0	0	0	0	0	0	0
27	23	15	16	29	19	20	5	27
1517	593	1085	1694	1432	955	1691	475	2569
98	111	1755	91	103	68	609	124	322
47	218	179	75	36	87	204	145	86
81	418	893	750	202	84	776	74	238
15	10	17	8	9	14	26	62	66
1653	418	633	2831	989	2835	1088	893	652
3	1	2	2	3	3	7	2	2
2	1	2	7	1	40	3	1	5
0	0	0	0	0	0	0	0	2
2	1	0	0	0	1	0	0	1
0	0	0	0	0	0	1	0	0
50340	37394	47093	92448	45776	38991	44269	39843	43161
492	319	479	629	569	728	588	621	1476
2	0	2	0	0	1	1	0	1
0	0	0	0	0	0	0	0	0
2	1	3	3	5	5	6	4	7
0	0	0	0	0	0	0	0	0

235	81	82	37	70	52	321	23	104
1	2	11	1	0	2	2	8	1
0	0	0	0	0	0	0	0	0
1	0	3	1	1	1	2	0	2
0	0	1	0	0	0	1	0	0
0	0	2	1	0	0	0	0	0
0	0	0	0	0	0	0	0	0
17	48	36	28	23	63	14	44	70
1	0	0	0	2	2	0	0	1
0	1	2	2	1	0	0	0	2
28	18	14	16	44	6	18	8	19
17	16	13	15	20	2	16	2	9
101	102	111	48	234	87	248	224	218
0	0	0	0	0	0	0	0	0
6	3	4	7	6	1	5	2	0
5	10	8	19	15	30	21	10	17
2	15	11	99	7	4	16	3	6
1	4	2	1	2	3	1	1	5
6	2	1	18	2	1	3	1	3
0	0	0	0	0	0	0	0	2
68158	29144	37825	50226	94587	56451	96481	26962	64309
3204	3959	7582	3719	2163	64892	4580	44242	30171
748	241	152	219	135	311	2233	951	291
4	3	4	8	10	15	16	9	18
249	338	685	415	202	2447	337	4757	3753
5	2	28	1	2	1	6	2	27
8	25	6	3	5	11	19	10	16
0	0	0	0	1	2	1	0	1
3	3	6	1	4	4	3	0	8
0	0	0	1	1	0	1	0	0
10	1	2	1	4	8	12	0	5
30	36	23	16	32	42	38	24	40
0	1	0	3	0	1	0	2	2
201	74	73	209	145	47	84	65	48
82	54	72	69	130	83	142	52	89
9	3	6	8	5	13	8	1	10
331	240	105	150	182	370	417	351	250
3	1	3	1	1	1	0	1	1
19	21	13	9	24	27	36	28	40
1	2	39	4	3	6	5	24	21
1	0	0	0	1	0	1	0	0
0	0	0	0	0	0	0	0	0
1	3	4	0	3	3	2	2	3
37	8	29	38	199	80	125	29	12
1957	764	1202	1816	1965	2487	2560	2306	3572
1	2	1	1	1	1	0	1	2
26698	6868	8184	15955	6787	19318	26347	25722	17326

3	5	5	6	3	0	12	0	2
50	69	78	51	54	238	107	435	416
4	57	67	246	74	16	111	7	30
535	411	488	579	692	329	980	308	1288
698	766	446	41	25	1288	1276	1359	1513
0	0	0	0	0	0	3	0	0
855	1564	852	423	926	628	650	380	1085
20	16	16	24	29	39	54	26	46
159	51	90	130	117	156	207	186	173
1	0	0	0	0	0	0	0	1
10	11	4	5	19	8	0	5	7
10	7	12	14	13	15	17	18	16
0	0	0	0	0	0	0	0	0
72270	25326	38493	74165	63684	35101	52206	34580	37123
6271	1450	2813	4625	2884	637	4475	2041	6897
0	0	0	0	0	0	0	0	0
16	4	5	12	7	8	4	10	22
3	35	0	2	7	1	592	115	6
3	0	1	1	0	0	3	1	5
5	2	16	2	4	3	2	3	12
3	7	202	8	2	15	5	159	45
1	2	0	0	1	0	0	1	0
3	0	4	5	3	6	6	6	5
0	0	0	0	0	0	0	0	0
5	10	9	42	1	7	9	1	4
75	27	42	40	27	27	51	32	77
6	6	10	6	7	5	16	15	11
121	3690	2088	18993	682	179	1351	75	268
272	226	357	222	207	243	678	111	474
125	60	68	130	109	41	87	39	68
419	64	142	156	99	64	222	199	537
19	4	1	6	2	3	15	7	6
11951	12529	7845	10113	23285	9051	14473	7228	15637
1	0	0	1	0	1	0	0	0
8	7	16	13	6	5	2	4	5
58	13	33	37	64	32	61	31	61
4	7	4	8	2	3	12	6	4
16469	13830	18745	25061	20546	13989	20388	15404	22955
25	17	29	21	33	46	37	62	70
2	4	3	3	3	5	4	2	4
1	0	0	0	2	1	0	0	2
0	0	0	0	0	0	0	0	0
17	87	60	63	24	27	32	92	54
887	376	441	179	83	291	602	279	191
104	71	77	84	112	94	186	62	109
286	171	1090	17	10	88	1185	473	928
1	1	3	2	3	4	1	1	1

0	0	0	0	0	0	0	1	0
0	0	0	1	0	0	0	1	0
74	164	89	159	354	63	72	73	151
3	7	3	2	3	4	3	6	9
29	9	38	22	35	45	44	63	41
8	44	13	4	11	23	19	7	29
32	13	11	19	39	21	40	14	28
20	19	10	9	19	8	6	24	19
7	7	9	19	28	2	9	14	14
48	36	32	5	15	27	68	42	51
6	2	8	3	6	11	5	12	23
31	4	8	16	19	25	23	24	31
111	173	235	85	130	39	234	129	235
15	5	8	7	21	10	13	8	15
2	9	2	3	4	3	1	0	50
0	0	0	0	0	0	0	0	0
196	3031	1042	201	680	1944	2104	338	1999
8	87	7	3	11	4	17	8	8
82	55	51	68	145	139	99	33	199
190	169	162	337	398	188	261	305	392
0	0	1	0	0	1	8	0	0
0	0	0	0	0	24	1	143	0
88624	48306	64566	87244	87948	76411	106179	81907	101965
27	34	32	35	33	22	73	30	56
0	4	4	4	1	4	13	2	0
25	17	20	25	42	32	31	6	26
0	0	0	0	0	1	0	1	1
0	1	0	3	0	0	0	0	0
40	164	56	51	24	40	74	153	38
46	8	36	202	37	20	138	26	53

NAIVEJJ-10-111	NAIVEJJ-10-112	NAIVEJJ-10-113	NAIVEJJ-10-114
Naive	Naive	Naive	Naive
4	3	14	5
213	612	977	266
24	0	0	0
8	2	6	0
299	679	424	22
7	3	3	3
23	4	5	5
15	8	9	10
0	0	0	0
153	50	83	20
0	0	1	1
154	105	128	72
6	4	11	1
13	30	54	17
59	24	30	9
2	2	3	1
483	3553	1536	2016
12	21	35	14
0	0	0	0
79	50	236	61
20920	53847	86052	34853
0	0	0	1
21	84	250	32
0	0	0	0
26	4	32	11
0	0	0	0
4	4	75	0
27	131	143	79
5	11	18	32
528	532	683	1132
0	0	1	0
13	6	8	3
6	8	20	10
1621	1445	2180	1370
11	9	3	4
35	20	34	16
1	1	9	4
0	1	3	1
92	76	82	60
0	0	0	1

2	1	3	1
84	115	167	64
10	1	1	2
20	9	37	11
2	0	0	1
12	24	22	15
21085	19343	40629	14443
193	65	183	24
0	1	1	3
377	84	65	4
752	496	934	702
59	93	106	179
13	406	348	57
1	0	0	0
25	0	0	0
3592	1056	2372	3516
357	527	449	1404
0	0	1	0
17	26	15	19
0	0	5	0
0	0	1	0
14506	367	158	109
359	102	108	20
51	8	16	16
3213	1054	2137	4325
0	0	0	1
8	2	5	2
128	17	114	68
0	0	0	0
11	12	27	21
1157	1160	2250	1061
7174	217	266	44
129	68	167	31
417	251	912	164
22	16	30	15
798	455	1396	604
2	4	7	4
3	7	3	9
0	0	1	0
0	1	4	0
0	0	1	0
22889	38122	45357	16238
459	483	578	231
0	0	1	1
0	0	0	0
0	3	7	1
0	0	0	0

123	74	122	80
31	1	6	1
0	0	0	0
1	0	1	0
1	0	0	0
0	0	0	0
0	0	0	0
24	9	20	25
0	0	1	1
1	0	0	0
17	14	22	24
17	13	16	12
174	111	326	189
0	0	0	0
1	2	1	4
16	18	24	17
24	13	12	6
3	0	3	4
4	2	4	0
0	0	0	0
43194	49293	74120	77634
3129	22780	1696	18347
263	1391	1887	1114
9	15	19	13
349	936	391	817
13	2	32	6
6	2	27	6
1	0	1	0
3	2	1	3
1	0	0	0
3	1	3	5
19	11	54	6
0	0	1	0
10	59	26	24
65	59	91	54
1	5	14	5
174	451	441	774
0	1	1	1
45	37	23	16
77	2	5	3
1	0	1	0
0	0	0	0
4	1	3	2
56	27	21	153
1030	1620	1625	1029
2	0	1	2
10569	10681	13671	21075

7	6	10	2
80	66	95	111
3	54	116	17
541	649	1280	581
1351	830	1989	313
0	0	0	0
620	280	587	514
26	14	16	26
101	121	163	139
0	0	0	1
15	1	7	11
10	10	17	13
0	0	0	0
16999	33665	28713	23355
559	3329	10835	1770
0	0	0	0
8	6	5	10
3	3	104	4
1	0	4	0
1	2	23	3
408	4	4	2
0	0	3	0
4	2	8	4
0	0	1	0
300	5	11	2
28	23	81	23
6	5	7	5
4668	1613	1657	256
227	67	280	204
58	77	41	36
247	228	237	30
4	4	4	6
10124	9318	11111	13441
0	0	0	0
2	3	1	5
19	34	64	33
9	9	8	2
15717	15202	19017	13057
23	37	28	24
9	4	8	3
0	0	0	1
0	0	0	0
74	77	36	16
343	633	456	399
83	38	96	130
462	1121	748	14
6	4	2	1

0	0	0	0
2	0	0	3
125	123	127	76
3	1	7	0
66	30	30	14
3	5	19	10
21	23	24	18
21	6	22	11
11	10	7	31
36	61	55	22
11	0	8	1
18	27	22	15
281	111	88	56
11	6	14	27
6	2	3	4
0	0	0	0
344	622	1188	846
7	8	18	6
46	62	49	138
193	230	225	317
0	0	2	0
0	0	0	0
62432	65909	86581	58807
36	47	76	30
0	1	1	6
13	17	35	22
0	0	0	0
124	4	2	3
25	27	70	35
129	8	97	10

Table S1H

**Family**	Family_Ace	Family_Ach	Family_Aci	Family_Aci	Family_Act	Family_Act	Family_Act	Family_Aer
Study variables: Dose/Time								
Gy_11_day	0.001471	0.108252	0.000245	0.00146	0.294596	0.000969	0.001158	0.003676
Gy_11_day	0.002257	0.070013	0.002149	0.00141	0.082873	0.001062	0.005705	0.007562
Gy_11_day	0.001892	0.07764	0.002402	0.000496	0.090713	0.001359	0.001384	0.017388
Gy_9.5_da	0.002376	0.093784	0.001295	0.001214	0.062571	0.001256	0.00248	0.008149
Gy_9.5_da	0.00276	0.166431	0.000555	0.003182	0.06303	0.000808	0.002931	0.003147
Gy_9.5_da	0.002386	0.083235	0.001332	0.000798	0.090463	0.000819	0.002142	0.007656
Naive	0.001736	0.13762	0.002148	0.00119	0.100572	0.001241	0.003011	0.003194

Family_Aer	Family_Alc	Family_Alc	Family_Alt	Family_Am	Family_Am	Family_An	Family_An	Family_An
0	0.010896	0	0.040714	0.001107	0.013968	0.028497	0.000568	0.565442
0.000238	0.049026	0	0.031483	0.001402	0.005323	0.013047	0.000105	0.124514
0.000227	0.077475	0.000105	0.030452	0.001545	0.00572	0.018331	0.000492	0.38116
0	0.026651	0	0.04775	0.002144	0.018532	0.008382	0.000588	0.414182
6.72E-05	0.054753	0.000101	0.025919	0.001612	0.006446	0.007971	0.000354	0.114922
0	0.020587	6.42E-05	0.039708	0.001214	0.008959	0.006106	0.00038	0.248396
0	0.023418	0.000133	0.033997	0.001509	0.007621	0.009359	0.000566	0.540307

Family_An	Family_Aur	Family_Bac	Family_Bac	Family_Bar	Family_Bd	Family_Bei	Family_Bifi	Family_Bo
0.004866	0	0.022277	9.153416	7.76E-05	0.007208	7.76E-05	0.01806	0
0.004875	9.65E-05	0.019264	10.41171	0	0.028152	0	0.004361	0
0.006347	0	0.025153	11.76428	0	0.026191	0	0.003266	0
0.005359	7.31E-05	0.020184	11.18467	0	0.010125	4.49E-05	0.006307	0
0.006442	0	0.073573	14.6663	0	0.018059	0	0.003352	0
0.004729	0.000101	0.052717	9.776508	0	0.026719	0.000113	0.004762	0
0.005604	0	0.028435	13.20281	7.81E-05	0.024191	0	0.005237	0

Family_Bor	Family_Bra	Family_Bra	Family_Bre	Family_Bru	Family_Bur	Family_Cal	Family_Cal	Family_Cal
0.019354	0.04145	0.003913	0.112448	0	0.001799	0.002763	0.404846	0.001568
0.004689	0.00498	0.005254	0.230909	0	0.001672	0.003858	0.546832	0.002018
0.005365	0.026478	0.001786	0.217298	0.000301	0.002135	0.002892	0.346036	0.00224
0.005392	0.05618	0.005126	0.347103	4.04E-05	0.003506	0.002931	0.432433	0.002165
0.002059	0.00384	0.005034	0.171418	0.000102	0.002544	0.005208	0.534496	0.001753
0.007037	0.019447	0.004678	0.16265	5.23E-05	0.002574	0.002449	0.434541	0.002204
0.00478	0.025871	0.004729	0.211501	5.51E-05	0.002269	0.002995	0.475954	0.002108

Family_Ca	Family_Car	Family_Car	Family_Cau	Family_Cel	Family_Cer	Family_Chi	Family_Chl	Family_Chr
0.005482	0.000707	0.000348	0.012828	9.03E-05	0.001013	0.035222	0.000233	0.003547
0.008865	0.001624	0.000195	0.017347	0	0.000627	0.025279	0.000736	0.004795
0.008763	0.003924	0.000455	0.018093	5.7E-05	0.00041	0.02145	0.000332	0.016033
0.007239	0.001355	0.000583	0.021752	4.49E-05	0.001179	0.031377	0.000564	0.005385
0.005461	0.001351	0.000471	0.0093	5.01E-05	0.000926	0.04057	0.000673	0.004145
0.006812	0.00216	0.000311	0.014493	0	0.000754	0.023834	0.000886	0.005838
0.007692	0.00097	0.000315	0.022894	7.81E-05	0.000494	0.029976	0.001178	0.005335

Family_Chr	Family_Chr	Family_Clo	Family_Co	Family_Co	Family_Co	Family_Cor	Family_Cor	Family_Cox
9.03E-05	0.007931	7.217055	0.019791	7.76E-05	0.004381	0.166963	0.030552	0.058549
0.000184	0.004614	8.318663	0.066675	0.000315	0.054223	0.447231	0.025681	0.002421
0	0.00373	7.259964	0.073223	0.000159	0.015011	0.185684	0.03831	0.001227
0.000153	0.007657	6.221365	0.030971	0.000468	0.004161	0.365527	0.025069	0.006604
0.000117	0.004704	6.68329	0.045914	0.000337	0.161026	0.378954	0.018672	0.001582
0	0.00729	5.981027	0.027297	7.11E-05	0.004136	0.420066	0.040772	0.020801
0.000257	0.005181	6.643136	0.0339	0.000361	0.043661	0.209217	0.031779	0.053934

Family_Cya	Family_Cys	Family_Def	Family_De	Family_De	Family_Dei	Family_Der	Family_Der	Family_De
0	0.000251	0.378266	0.07976	0	0.00836	0.000587	0	0.0582
5.64E-05	0.001776	0.61035	0.20533	0	0.004545	0.000767	0	0.632768
0	0.002329	1.351391	0.085081	0	0.007952	0.001087	0	0.94755
9.54E-05	0.00263	0.643017	0.120433	8.53E-05	0.004585	0.000562	0	0.382448
0	0.00091	0.627679	0.192247	0.000101	0.003471	0.000438	0	0.416612
0.000142	0.000747	0.177439	0.24848	7.11E-05	0.004119	0.000784	0	0.466602
8.95E-05	0.002237	0.802636	0.204182	5.51E-05	0.005698	0.000276	5.51E-05	1.341694

Family_De	Family_De	Family_De	Family_De	Family_De	Family_Det	Family_Die	Family_Ect	Family_Ent
0.018741	0.002164	0.256956	0	0.000168	0.024685	0	0.00243	0.336803
0.046117	0.003867	0.783081	0	0.00103	0.042319	0	0.005345	0.357169
0.017284	0.004309	0.335364	0	0.001478	0.046245	0	0.003776	4.089482
0.027686	0.003573	0.778233	0	0.00169	0.045457	0	0.003516	0.381918
0.072758	0.003421	0.676255	0	0.000708	0.031966	0	0.005616	0.290686
0.021919	0.00318	0.786388	0	0.000847	0.04463	0	0.003968	0.312333
0.046962	0.00727	0.818809	7.81E-05	0.001291	0.024272	0	0.004974	0.393707

Family_Ent	Family_Ent	Family_Ery	Family_Ery	Family_Eub	Family_Euz	Family_Exi	Family_Fer	Family_Fib
0.084649	0.014936	0.042222	0.00527	0.176796	0.001099	0.000855	7.76E-05	0.000328
0.115688	0.02184	0.084137	0.010098	0.257455	0.000952	0.000606	5.64E-05	0.000108
0.288311	0.026883	0.053654	0.018761	0.166517	0.000817	0.004158	0.000481	0.000113
0.102243	0.022837	0.072854	0.00799	0.254667	0.00052	0.001151	0	0.00013
0.139005	0.039721	0.150234	0.003015	0.276988	0.000488	0.000637	0	0.000101
0.059884	0.030391	0.061154	0.010032	0.332858	0.000783	0.003047	0.000105	0.000116
0.675672	0.028053	0.118424	0.005943	0.228212	0.001164	0.001639	5.51E-05	0.000292

Family_Fla	Family_Fla	Family_Fle	Family_Fra	Family_Fra	Family_Fus	Family_Gal	Family_Ge	Family_Ge
9.03E-05	11.67767	0.201224	0.000371	0	0.000552	0	0.086941	0.000448
4.82E-05	8.121153	0.153807	4.91E-05	0	0.00051	0	0.04084	0.001657
6.02E-05	4.156587	0.104059	0.000152	0	0.000657	4.77E-05	0.020859	0.001745
0.000117	7.744921	0.129411	0.000281	0	0.000884	0	0.026364	0.001562
0	13.91377	0.124966	0.000134	0	0.000707	0	0.019136	0.000992
4.9E-05	9.762364	0.196886	0.000166	0	0.001266	0	0.026153	0.000847
5.51E-05	8.552839	0.125644	0.000133	0	0.000679	0	0.029292	0.003255

Family_Ge	Family_Gly	Family_Go	Family_Go	Family_Ha	Family_Hal	Family_Hal	Family_Hal	Family_Hal
0	0.000384	0.000258	0	0	0.008707	7.76E-05	9.03E-05	0.004203
0	0.000221	4.91E-05	0	0	0.011167	0	0.000306	0.006213
0	0.000788	9.54E-05	0	0	0.016672	0	0.000318	0.007931
0	0.000451	7.31E-05	0	0	0.032501	0.000319	0.000231	0.007284
0	0.000303	6.72E-05	0.000185	0	0.009858	0.000102	0.000387	0.005804
0	0.000267	4.9E-05	0	0	0.011518	0.000181	0.000105	0.00283
0	0.000145	8.95E-05	0	0	0.005849	0.000133	8.95E-05	0.005613

Family_Hel	Family_Hel	Family_Hol	Family_Hy	Family_Hy	Family_Hy	Family_Idio	Family_Kilo	Family_Kin
0.0028	0.054135	0	0.000599	0.00507	0.003983	0.000526	0.001316	0
0.002549	0.04199	0	0.000417	0.005871	0.004017	0.000755	0.000929	0
0.002656	0.106375	0	0.000364	0.004262	0.002205	0.001079	0.000572	0
0.004657	0.054322	0	0.001195	0.005127	0.001209	0.00109	0.001132	3.66E-05
0.004264	0.032102	0	0.001229	0.003266	0.007574	0.000692	0.001273	0
0.001526	0.045068	0	0.000452	0.004555	0.001568	0.000574	0.000439	0.000105
0.004274	0.056274	0	0.000601	0.005375	0.004211	0.000746	0.000722	0

Family_Lac	Family_Lac	Family_Leg	Family_Lep	Family_Leu	Family_List	Family_Lito	Family_Me	Family_Me
12.4067	7.092651	0.443389	0.002813	0.347395	0.000431	0.002401	0.000371	0.001924
17.71423	5.788479	0.087004	0.001387	0.660964	0.000538	0.002528	0.000291	0.000768
15.77759	15.03357	0.311721	0.000785	1.330629	0.000991	0.002279	0.000543	0.001128
19.00851	3.034735	0.336915	0.004245	0.24955	0.001031	0.002846	0.000176	0.000635
12.82095	1.207231	0.052483	0.001482	0.111364	0.002236	0.003343	5.08E-05	0.000961
13.63391	9.111378	0.21228	0.003328	0.707944	0.001913	0.003185	0.00023	0.000822
17.553	3.441013	0.3144	0.003945	0.183763	0.00354	0.002638	0.000145	0.000701

Family_Me	Family_Me	Family_Mic	Family_Mic	Family_Mic	Family_Mic	Family_Mic	Family_Mo	Family_My
0	0.000181	0.005391	0.000419	0.033461	0.013957	0.001518	0.124392	0.000491
0.000289	0.001163	0.009123	5.64E-05	0.009889	0.024733	0.002383	0.133094	0.000233
0.000108	0.00085	0.008689	4.77E-05	0.002705	0.040762	0.001518	0.046816	0.000453
0	0.000928	0.008674	9.54E-05	0.028187	0.028061	0.002125	0.090996	0.000516
0.000101	0.000489	0.007624	0.000252	0.030248	0.020379	0.001362	0.048156	0.000404
4.9E-05	0.001363	0.008357	0.000311	0.014266	0.020641	0.001821	0.082217	0.000188
8.95E-05	0.000896	0.005936	5.51E-05	0.008436	0.019286	0.001611	0.132703	0.000205

Family_My	Family_Na	Family_Nei	Family_Nit	Family_No	Family_No	Family_No	Family_Oc	Family_Od
0.007075	0.00078	0	0	0.000719	0.004234	0.654783	0.000406	3.420259
0.007142	0.004655	0.000157	0	0.000782	0.007406	0.401975	0.000319	3.565218
0.007737	0.004921	0.001501	0	0.000797	0.001277	0.269992	0.001618	4.404409
0.006672	0.004996	8.15E-05	9.54E-05	0.00077	0.026879	0.593382	0.000558	3.125017
0.004674	0.003177	5.08E-05	0	0.000726	0.014779	0.349092	0.000371	2.390519
0.00758	0.003434	4.9E-05	0	0.00059	0.013953	0.635859	0.00024	5.266147
0.0092	0.007544	0.000145	0	0.000751	0.020057	0.378383	0.00039	4.112125

Family_Ox	Family_Pae	Family_Par	Family_Pas	Family_Pel	Family_Pel	Family_Pep	Family_Pep	Family_Ph
0.000797	0.042005	0.004897	0.186941	0.277265	0	0.234361	0.004548	0.040302
0.001647	0.072624	0.011502	0.144767	0.493034	0	0.195422	0.007044	0.027418
0.000236	0.130585	0.015488	0.221805	0.583902	0.000162	0.138217	0.004693	0.027593
0.000906	0.036248	0.004396	0.215625	0.202503	0	0.239477	0.005968	0.039651
0.001296	0.017538	0.026365	0.138647	0.110968	0	0.284101	0.005374	0.023678
0.000693	0.073195	0.008537	0.158427	0.320953	0.000147	0.155931	0.009405	0.042431
0.001765	0.025803	0.011867	0.210939	0.314574	0	0.148088	0.006244	0.037565

Family_Phy	Family_Pis	Family_Pla	Family_Pol	Family_Por	Family_Pre	Family_Pro	Family_Pro	Family_Pse
0	0.000682	0.002125	0	11.7511	0.95299	7.76E-05	0.002161	0.0197
0	0.001879	0.003727	0.000168	7.251805	0.4189	0	0.002526	0.016011
0	0.004276	0.005662	0.000165	4.007017	0.103641	4.77E-05	0.005655	0.025272
0.00014	0.002329	0.002987	0.000172	10.81221	0.714879	0	0.002353	0.00461
0	0.0026	0.002878	0	12.10678	0.714326	0	0.001698	0.004005
5.23E-05	0.001235	0.003912	0	9.212124	0.766114	0	0.002571	0.037585
7.81E-05	0.002659	0.003565	0	7.344109	1.024573	0	0.002203	0.006531

Family_Pse	Family_Pse	Family_Pse	Family_Psy	Family_Pu	Family_Rh	Family_Rhi	Family_Rh	Family_Rh
0.000406	0.000712	0.000358	0.000168	0.001273	0	0.001525	0.015312	0.001688
0.000318	0.000986	0.019787	9.28E-05	0.000541	0	0.002936	0.009162	0.003254
0.00151	0.001588	0.025653	0.000462	0.001533	0	0.002517	0.0105	0.004862
0.000918	0.001467	0.015443	0.00033	0.001224	0.000116	0.001594	0.01267	0.002245
0.000117	0.001582	0.014789	0.000254	0.000672	0	0.003773	0.008936	0.001937
0.00048	0.001131	0.014862	7.11E-05	0.001367	0	0.001171	0.010535	0.002747
0.00031	0.001736	0.037176	0.000165	0.001255	5.51E-05	0.027968	0.010419	0.001672

Family_Rh	Family_Rh	Family_Ric	Family_Rik	Family_Riv	Family_Ru	Family_Sac	Family_Sap	Family_She
0.579641	0.056545	0.029078	0.052606	0.003822	2.424696	0	0.001591	0.013793
0.518038	0.156581	0.01266	0.023209	0.001308	4.779036	0	0.000769	0.008913
0.061328	0.223347	0.016314	0.00734	0.000926	3.875289	0.00012	0.000727	0.006425
0.127168	0.052053	0.022411	0.027149	0.002624	3.052116	4.49E-05	0.001865	0.012532
1.500382	0.068551	0.022706	0.028879	0.000875	3.487781	5.01E-05	0.002741	0.008643
0.09709	0.081524	0.013226	0.057223	0.001739	2.622153	6.42E-05	0.000965	0.010439
0.644831	0.056495	0.015788	0.053874	0.001334	3.237067	0	0.00084	0.010246

Family_Sin	Family_Sph	Family_Sph	Family_Spi	Family_Spo	Family_Spo	Family_Sta	Family_Str	Family_Str
0.001934	4.383844	0.014628	0.00079	0	0	0.015235	0.139799	0.015269
0.001608	4.784877	0.013575	0.000678	6.37E-05	0	0.008533	0.091946	0.024121
0.002653	2.085603	0.026055	0.00039	0	0	0.029555	0.207212	0.023788
0.001601	4.537889	0.009808	0.001129	0.000122	0	0.007012	0.097528	0.034183
0.001481	4.96172	0.006402	0.00091	0.000102	0	0.017229	0.080949	0.022275
0.001417	4.192963	0.012834	0.000868	0.000169	0	0.012665	0.078017	0.025261
0.002048	4.565694	0.008131	0.001768	7.81E-05	0	0.015382	0.132421	0.025602

Family_Str	Family_Sul	Family_Sul	Family_Sy	Family_Syn	Family_Syn	Family_Syn	Family_Syn	Family_The
0.455399	0.000825	0	7.76E-05	0.043443	0.001831	0.008603	0.006701	0.006718
0.112338	0.000756	8.91E-05	0.000257	0.029402	0.000814	0.006814	0.00357	0.005607
0.117691	0.001148	0.000111	0.000216	0.046012	0.000494	0.006361	0.003108	0.00504
0.09729	0.000777	0	7.31E-05	0.043226	0.000383	0.01069	0.002363	0.00592
0.091959	0.000556	0	5.01E-05	0.048569	0.001164	0.006348	0.006095	0.004992
0.145914	0.000429	7.11E-05	7.11E-05	0.02066	0.001301	0.011668	0.004422	0.005769
0.164112	0.001012	0	0.000413	0.03295	0.000726	0.010806	0.002456	0.006259

Family_TheFamily_TheFamily_TheFamily_TheFamily_TheFamily_TheFamily_TheFamily_TheFamily_The

0.002907	0.001797	0.016739	0.001607	0.006496	0.024076	0.002085	0.001252	0
0.003403	0.004298	0.009178	0.003325	0.005279	0.038456	0.003893	0.001603	0
0.008639	0.002558	0.01119	0.000972	0.004306	0.028753	0.003436	0.000645	0
0.00375	0.002638	0.007413	0.001996	0.005762	0.033918	0.003281	0.001204	0
0.004015	0.00369	0.006814	0.001196	0.00271	0.044201	0.002463	0.001401	0
0.003507	0.002297	0.01072	0.003007	0.006059	0.035438	0.002632	0.002857	0
0.004382	0.004509	0.012349	0.001504	0.005933	0.042337	0.004296	0.001158	0

Family_The	Family_The	Family_Thi	Family_Thi	Family_Tsu	Family_Tur	Family_Un	Family_Vei	Family_Ver
0.254673	0.001601	0.008823	0.046466	0	0	21.24722	0.014357	0.001274
0.402885	0.002088	0.019096	0.089272	6.37E-05	0	19.43101	0.01033	0.000823
0.259364	0.001991	0.008259	0.061466	0.000241	0	17.56373	0.007386	0.004578
0.136231	0.002337	0.022074	0.067132	0	8.08E-05	22.26987	0.008018	0.000378
0.421992	0.010001	0.019781	0.065134	6.72E-05	0	18.07768	0.009029	0.000926
0.356507	0.002077	0.026528	0.067044	0.000457	0.011753	21.26504	0.010053	0.001036
0.206999	0.002662	0.022046	0.070941	0.00011	0	19.68331	0.013128	0.000596

Family_Vib	Family_Wa	Family_Xan	Family_Xan	Family_Yaniellaceae
0.004605	0	0.000388	0.010964	0.013216
0.005704	0.000101	0.000425	0.016961	0.031567
0.012402	0.00016	0.001017	0.02381	0.005483
0.009174	0	3.66E-05	0.011133	0.012547
0.006455	0	0.000252	0.024167	0.015228
0.00536	0.000188	0	0.019058	0.012671
0.006031	0	0.011728	0.010768	0.018248

Table S2

Day 3

	9.5Gy	11Gy
p_bacteroidetes	3.3	
c_chitinophagia	2	
o_chitinophagales	2.7	
f_chitinophagaceae	2.2	
f_dermabacteraceaes		3.2
f_porphyromonadaceaea	2.1	
g_blautia		2.3
g_brachybacteriums		3
g_chitinophaga	3.7	
g_desulfovibrio		2.7
g_hellicobacter	2.3	
g_johnsonella		2.2
g_lysinibacillus	2.6	
g_parapedobacter		3.1
g_porphyromonas	3.1	
g_vivrio	2.7	

Day 9

p_deferribacteres		2.1
p_firmicutes		3.6
c_clostridia		3.7
c_deferribacteres		2
c_flavobacteriia	2.6	
c_nostocophycideae	2.2	
o_clostridiales		3.2
o_coribacteriales	2.8	
o_deferribacterales		2.7
o_flavobacteriales	2.9	
o_nostocales	2.9	
f_coribacteriacae	2.2	
f_deferribacteraceae		2.2
f_flavobacteriaceae	2.6	
f_lachnospiraceae		2
f_nostocaceae	3.3	
f_porphyromomadaceaea	3.6	
g_agromyces		3.1
g_dysgonomonas	2	
g_enterobacter		2.6
g_escheichia		3.4
g_johnsonella		3
g_muscispirillum		2
g_olivibacter	3	
g_pophyromonas	2.2	

g_serratia	3.1

Table S3

Radiation	Day post-radiation
**11 Gy**	**1d**
	3d
	9d

9.5	1d
	3d
	9d

Pathway	LDA
L-arginine biosynthesis IV (archaebacteria)	2.68
acetylene degradation	2.66
L-lysine biosynthesis I	2.65
GDP-D-glycero-α-D-manno-heptose biosynthesis	2.51
L-arginine biosynthesis I (via L-ornithine)	2.32
sucrose degradation IV (sucrose phosphorylase)	2.3
CMP-3-deoxy-D-manno-octulosonate biosynthesis I	-2.3
lipid IVA biosynthesis	-2.4
Kdo transfer to lipid IVA III (Chlamydia)	-2.4
acetylene degradation	2.7
L-lysine biosynthesis I	2.69
CDP-diacylglycerol biosynthesis II	2.67
Bifidobacterium shunt	2.62
L-arginine biosynthesis II (acetyl cycle)	2.59
L-lysine biosynthesis II	2.57
chorismate biosynthesis I	2.41
CDP-diacylglycerol biosynthesis I	2.32
polyisoprenoid biosynthesis (E. coli)	-2.5
superpathway of thiamin diphosphate biosynthesis I	-2.6
phosphopantothenate biosynthesis I	-2.7
superpathway of GDP-mannose-derived O-antigen building blocks biosynthesis	-2.7
CMP-3-deoxy-D-manno-octulosonate biosynthesis I	-2.7
lipid IVA biosynthesis	-2.8
Kdo transfer to lipid IVA III (Chlamydia)	-2.8
fatty acid elongation -- saturated	-2.8
preQ0 biosynthesis	-2.9
pyruvate fermentation to propanoate I	-2.9
6-hydroxymethyl-dihydropterin diphosphate biosynthesis III (Chlamydia)	-2.9
6-hydroxymethyl-dihydropterin diphosphate biosynthesis I	-2.9
thiazole biosynthesis I (E. coli)	-2.9
taxadiene biosynthesis (engineered)	3.03
glycerol degradation to butanol	2.76
heterolactic fermentation	2.75

superpathway of geranylgeranyldiphosphate biosynthesis I (via mevalonate)	2.61
lactose and galactose degradation I	2.53
superpathway of L-tryptophan biosynthesis	2.53
mevalonate pathway I	2.5
teichoic acid (poly-glycerol) biosynthesis	2.49
hexitol fermentation to lactate, formate, ethanol and acetate	2.29
superpathway of (Kdo)2-lipid A biosynthesis	2.26
superpathway of chorismate metabolism	2.23
superpathway of menaquinol-10 biosynthesis	2.23
superpathway of menaquinol-6 biosynthesis I	2.22
glyoxylate cycle	2.19
superpathway of menaquinol-9 biosynthesis	2.19
enterobactin biosynthesis	2.16
superpathway of demethylmenaquinol-9 biosynthesis	2.13
superpathway of demethylmenaquinol-6 biosynthesis I	2.1
superpathway of L-threonine metabolism	2.09
ppGpp metabolism	2.09
glucose and glucose-1-phosphate degradation	2.03
enterobacterial common antigen biosynthesis	2
L-histidine biosynthesis	-2.6
superpathway of menaquinol-8 biosynthesis II	-2.6
chondroitin sulfate degradation I (bacterial)	-2.7
L-arginine biosynthesis III (via N-acetyl-L-citrulline)	-2.9
L-methionine biosynthesis III	-3
purine ribonucleosides degradation	2.8
L-arginine biosynthesis I (via L-ornithine)	2.54
L-arginine biosynthesis IV (archaebacteria)	2.42
superpathway of thiamin diphosphate biosynthesis I	-2.4
superpathway of GDP-mannose-derived O-antigen building blocks biosynthesis	-2.6
pyruvate fermentation to propanoate I	-2.7
preQ0 biosynthesis	-2.7
L-arginine biosynthesis II (acetyl cycle)	2.64
dTDP-N-acetylthomosamine biosynthesis	2.31
nitrate reduction VI (assimilatory)	2.12
superpathway of purine deoxyribonucleosides degradation	2.78

acetylene degradation	2.75
mixed acid fermentation	2.7
1,4-dihydroxy-6-naphthoate biosynthesis II	2.66
1,4-dihydroxy-6-naphthoate biosynthesis I	2.63
guanosine ribonucleotides de novo biosynthesis	2.6
TCA cycle VIII (helicobacter)	2.54
urea cycle	2.5
teichoic acid (poly-glycerol) biosynthesis	2.45
hexitol fermentation to lactate, formate, ethanol and acetate	2.43
L-tryptophan biosynthesis	-2.5
6-hydroxymethyl-dihydropterin diphosphate biosynthesis III (Chlamydia)	-2.6
superpathway of thiamin diphosphate biosynthesis I	-2.7
thiazole biosynthesis I (E. coli)	-2.7
preQ0 biosynthesis	-2.8

Tabke S4

9-5Gy-d1	Name
HMDB0240259	L-urobilin
HMDB0013200	5-hydroxytryptophol glucuronide
HMDB0000488	4E,15Z-bilirubin IXa
HMDB0000054	bilirubin
HMDB0011692	2'-cytidylic acid
HMDB0000095	5'-CMP
HMDB0001830	progesterone
HMDB0001898	mesobilirubinogen
HMDB0005084	N-acetylleukotriene e4
HMDB0009928	16:0/20:2(11Z,14Z) phosphatidylinositol-3-phosphate
HMDB0006059	20-carboxy-leukotriene B4
HMDB0002596	deoxycholic acid 3-glucuronide
HMDB0003128	cortolone
HMDB0001403	prostaglandin D2
HMDB0004161	D-urobilin
HMDB0011563	1-pentadecanoylglycerol
HMDB0062389	4alpha-formyl-5alpha-cholesta-8,24-dien-3beta-ol
HMDB0006709	ubiquinone Q2
HMDB0006888	5beta-cyprinolsulfate
HMDB0060766	4-hydroxyatomoxetine
HMDB0032857	tripropionin
HMDB0062552	goralatide
HMDB0031039	heptadecanal
HMDB0000253	pregnenolone
HMDB0003034	prostaglandin D3
HMDB0011517	1-arachidonoyl-2-hydroxy-sn-glycero-3-phosphoethanolamine
HMDB0000273	thymidine
HMDB0002314	11,12-DHET
HMDB0002265	14,15-DHET
HMDB0004234	12-keto-leukotriene B4
HMDB0062298	15-deoxy-delta-12-14 prostaglandin D2
HMDB0060095	prostaglandin C2
HMDB0062747	12(S)-hydroxyeicosatrienoic acid
HMDB0013243	Leu-Phe
HMDB0000306	tyramine
HMDB0001542	N-acetyllactosamine
HMDB0000626	deoxycholate
HMDB0029160	gamma-glutamyltryptophan
HMDB0003533	3,7,12-trihydroxycholestan-26-al
HMDB0000866	N-acetyl-L-tyrosine
HMDB0062251	alanine
HMDB0001262	maltotriose
HMDB0004256	7-hydroxy-6-methyl-8-ribityl lumazine
HMDB0003406	D-serine

HMDB0006869	S-(2-methylbutanoyl)-dihydrolipoamide
HMDB0061705	6-oxopiperidine-2-carboxylic acid
HMDB0011538	2-linoleoylglycerol
HMDB0010727	3-oxododecanoate
HMDB0060987	2-hydroxy methyl olanzapine
HMDB0059889	isohexyl alcohol
HMDB0000988	S-adenosyl-3-methylthiopropylamine
HMDB0062698	saccharopine
HMDB0039066	6',7'-dihydroxybergamottin
HMDB0005066	tetradecanoylcarnitine
HMDB0006116	3-hydroxyhippuric acid
HMDB0011506	1-oleoyl-lysophosphatidylethanolamine
HMDB0006547	stearidonic acid
HMDB0061701	2-18:1(9Z) lysophosphatidylcholine
HMDB0041927	monomethylpropion
HMDB0013122	dm18:0 lysoplasmenylcholine
HMDB0004949	D-erythro-C16-ceramide
HMDB0011564	1-palmitoylglycerol
HMDB0011533	2-palmitoylglycerol
HMDB0061702	2-16:0 lysophosphatidylcholine
HMDB0000207	oleic acid
HMDB0000860	phenylpropionylglycine
HMDB0010392	1-20:2(11Z,14Z) lysophosphatidylcholine
HMDB0007070	1-15:0/2-16:1(9Z) diacylglycerol
HMDB0000220	palmitic acid
HMDB0011511	1-20:0 lysophosphatidylethanolamine
HMDB0010381	1-15:0 lysophosphatidylcholine
HMDB0011130	1-stearoyl-2-hydroxy-sn-glycero-3-phosphoethanolamine
HMDB0010388	1-18:3(9Z,12Z,15Z) lysophosphatidylcholine
HMDB0010391	1-20:1(11Z) lysophosphatidylcholine
HMDB0010382	1-16:0 lysophosphatidylcholine
HMDB0011512	1-20:1(11Z) lysophosphatidylethanolamine
HMDB0010385	1-18:1(11Z) lysophosphatidylcholine
HMDB0011128	2-18:0 lysophosphatidylcholine
HMDB0010384	L-alpha-lysophosphatidylcholine, stearoyl
HMDB0010390	1-20:0 lysophosphatidylcholine
HMDB0006819	2-hexaprenyl-3-methyl-6-methoxy-1,4-benzoquinone
HMDB0004160	I-urobilin
HMDB0008028	16:1(9Z)/dm18:0 phosphatidylcholine
HMDB0011769	d18:0/24:1 dihydroceramide
HMDB0004956	d18:1/24:0 ceramide
HMDB0007210	1-18:1(11Z)/2-24:1(15Z) diacylglycerol
HMDB0007239	1-18:1(9Z)/2-24:1(15Z) diacylglycerol
HMDB0004952	d18:1/22:0 ceramide
HMDB0240261	18:0 lysophosphatidylinositol
HMDB0061704	2-18:0 lysophosphatidylinositol
HMDB0007982	1-palmitoyl-2-arachidonoyl-phosphatidylcholine

HMDB0007880	14:0/20:2(11Z,14Z) phosphatidylcholine

Type	Expression	9-5Gy-d3
chemical - endogenous mammalian	4.046	HMDB0007
chemical - endogenous mammalian	3.558	HMDB0003
chemical - endogenous mammalian	3.365	HMDB0010
chemical - endogenous mammalian	3.365	HMDB0007
chemical - endogenous mammalian	3.225	HMDB0240
chemical - endogenous mammalian	3.225	HMDB0002
chemical - endogenous mammalian	3.127	HMDB0013
chemical - endogenous mammalian	3.09	HMDB0005
chemical - endogenous mammalian	2.929	HMDB0009
chemical - endogenous mammalian	2.805	HMDB0010
chemical - endogenous mammalian	2.671	HMDB0006
chemical - endogenous mammalian	2.336	HMDB0001
chemical - endogenous mammalian	2.103	HMDB0005
chemical - endogenous mammalian	1.998	HMDB0008
chemical - endogenous mammalian	1.986	HMDB0001
chemical - endogenous mammalian	1.963	HMDB0062
chemical - endogenous mammalian	1.847	HMDB0002
chemical - endogenous mammalian	1.737	HMDB0011
chemical - endogenous mammalian	1.73	HMDB0000
chemical - endogenous mammalian	1.69	HMDB0000
chemical - endogenous mammalian	1.687	HMDB0006
chemical - endogenous mammalian	1.607	HMDB0000
chemical - endogenous mammalian	1.59	HMDB0000
chemical - endogenous mammalian	1.48	HMDB0010
chemical - endogenous mammalian	1.459	HMDB0011
chemical - endogenous mammalian	1.427	HMDB0011
chemical - endogenous mammalian	1.397	HMDB0000
chemical - endogenous mammalian	1.389	HMDB0062
chemical - endogenous mammalian	1.389	HMDB0006
chemical - endogenous mammalian	1.36	HMDB0060
chemical - endogenous mammalian	1.36	HMDB0001
chemical - endogenous mammalian	1.36	HMDB0006
chemical - endogenous mammalian	1.29	HMDB0031
chemical - endogenous mammalian	1.289	HMDB0011
chemical - endogenous mammalian	1.243	HMDB0009
chemical - endogenous mammalian	1.081	HMDB0010
chemical - endogenous mammalian	0.961	HMDB0006
chemical - endogenous mammalian	0.901	HMDB0004
chemical - endogenous mammalian	0.86	HMDB0002
chemical - endogenous mammalian	0.834	HMDB0005
chemical - endogenous mammalian	-0.768	HMDB0010
chemical - endogenous mammalian	-0.822	HMDB0008
chemical - endogenous mammalian	-0.856	HMDB0061
chemical - endogenous mammalian	-0.897	HMDB0002

chemical - endogenous mammalian	-0.906	HMDB0059
chemical - endogenous mammalian	-0.931	HMDB0006
chemical - endogenous mammalian	-0.991	HMDB0062
chemical - endogenous mammalian	-1.01	HMDB0001
chemical - endogenous mammalian	-1.033	HMDB0010
chemical - endogenous mammalian	-1.084	HMDB0000
chemical - endogenous mammalian	-1.12	HMDB0002
chemical - endogenous mammalian	-1.167	HMDB0060
chemical - endogenous mammalian	-1.215	HMDB0060
chemical - endogenous mammalian	-1.238	HMDB0011
chemical - endogenous mammalian	-1.262	HMDB0032
chemical - endogenous mammalian	-1.381	HMDB0002
chemical - endogenous mammalian	-1.384	HMDB0001
chemical - endogenous mammalian	-1.421	HMDB0011
chemical - endogenous mammalian	-1.456	HMDB0000
chemical - endogenous mammalian	-1.494	HMDB0010
chemical - endogenous mammalian	-1.527	HMDB0010
chemical - endogenous mammalian	-1.543	HMDB0062
chemical - endogenous mammalian	-1.543	HMDB0011
chemical - endogenous mammalian	-1.561	HMDB0000
chemical - endogenous mammalian	-1.575	HMDB0004
chemical - endogenous mammalian	-1.581	HMDB0062
chemical - endogenous mammalian	-1.588	HMDB0000
chemical - endogenous mammalian	-1.623	HMDB0000
chemical - endogenous mammalian	-1.631	HMDB0062
chemical - endogenous mammalian	-1.661	HMDB0060
chemical - endogenous mammalian	-1.664	HMDB0013
chemical - endogenous mammalian	-1.664	HMDB0006
chemical - endogenous mammalian	-1.688	HMDB0010
chemical - endogenous mammalian	-1.71	HMDB0005
chemical - endogenous mammalian	-1.72	HMDB0011
chemical - endogenous mammalian	-1.755	HMDB0006
chemical - endogenous mammalian	-1.804	HMDB0000
chemical - endogenous mammalian	-1.921	HMDB0013
chemical - endogenous mammalian	-1.921	HMDB0010
chemical - endogenous mammalian	-1.996	HMDB0011
chemical - endogenous mammalian	-2.158	HMDB0005
chemical - endogenous mammalian	-2.225	HMDB0060
chemical - endogenous mammalian	-2.242	HMDB0000
chemical - endogenous mammalian	-2.481	HMDB0002
chemical - endogenous mammalian	-2.481	HMDB0011
chemical - endogenous mammalian	-2.632	HMDB0008
chemical - endogenous mammalian	-2.632	HMDB0000
chemical - endogenous mammalian	-2.752	HMDB0000
chemical - endogenous mammalian	-3.342	HMDB0028
chemical - endogenous mammalian	-3.342	HMDB0007
chemical - endogenous mammalian	-3.55	HMDB0001

chemical - endogenous mammalian	-3.913	HMDB0000
		HMDB0001
		HMDB0000
		HMDB0000
		HMDB0000
		HMDB0039
		HMDB0002
		HMDB0062
		HMDB0004
		HMDB0005
		HMDB0011
		HMDB0000
		HMDB0061
		HMDB0000
		HMDB0000
		HMDB0001
		HMDB0002
		HMDB0041
		HMDB0001
		HMDB0000
		HMDB0008
		HMDB0001
		HMDB0010
		HMDB0010
		HMDB0000
		HMDB0033
		HMDB0002
		HMDB0033
		HMDB0000
		HMDB0010
		HMDB0004
		HMDB0061
		HMDB0000
		HMDB0006
		HMDB0000
		HMDB0062
		HMDB0000
		HMDB0006
		HMDB0000
		HMDB0035
		HMDB0060
		HMDB0003
		HMDB0062
		HMDB0006
		HMDB0000
		HMDB0006
		HMDB0000

HMDB0061
HMDB0000
HMDB0001
HMDB0094
HMDB0062
HMDB0000
HMDB0061
HMDB0000
HMDB0006
HMDB0003
HMDB0000
HMDB0000
HMDB0000
HMDB0006
HMDB0013
HMDB0000
HMDB0004
HMDB0062
HMDB0001
HMDB0013
HMDB0007
HMDB0000
HMDB0006
HMDB0000
HMDB0003
HMDB0013
HMDB0003
HMDB0061
HMDB0060
HMDB0006
HMDB0011
HMDB0006
HMDB0041
HMDB0013
HMDB0062
HMDB0000
HMDB0011
HMDB0000
HMDB0001
HMDB0000
HMDB0029
HMDB0012
HMDB0028
HMDB0028
HMDB0011
HMDB0000
HMDB0004

HMDB0000
HMDB0011
HMDB0006
HMDB0004
HMDB0006
HMDB0003
HMDB0000
HMDB0012
HMDB0000
HMDB0001
HMDB0000
HMDB0000
HMDB0000
HMDB0004
HMDB0013
HMDB0041
HMDB0000
HMDB0005
HMDB0002
HMDB0011
HMDB0004

Name
15:0/14:0 phosphatidylcholine
cortolone
beta-D-glucuronoside
1-15:0/2-16:0 diacylglycerol
L-urobilin
adrenic acid
5-hydroxytryptophol glucuronide
eicosa-11Z, 14Z-dienoic acid
16:0/20:4(5Z,8Z,11Z,14Z) phosphatidylinositol-3-phosphate
mead acid
5beta-cyprinolsulfate
progesterone
N-acetylleukotriene e4
14:0/22:0 phosphatidylethanolamine
hexadecanal
9Z-eicosenoic acid
gondoic acid
1-pentadecanoylglycerol
ergocalciferol
ergosterol
heptadecanoylcarnitine
bilirubin
24,25-dihydroxyvitamin D3
1-20:0 lysophosphatidylcholine
1-alkyl-2-lyso-sn-glycero-3-phosphocholine
1-arachidonoyl-2-hydroxy-sn-glycero-3-phosphoethanolamine
sphingosine-1-phosphate
platelet activating factor-C16
20-carboxy-leukotriene B4
dehydrodolichol diphosphate
mesobilirubinogen
alpha-linolenyl carnitine
heptadecanal
1-20:0 lysophosphatidylethanolamine
16:0/20:2(11Z,14Z) phosphatidylinositol-3-phosphate
1-20:1(11Z) lysophosphatidylcholine
beta-tocopherol
(5Z,8Z,11Z,13E)-15(S)-hydroperoxyeicosatetraenoic acid
prostaglandin B1
pregnane
1-18:3(6Z,9Z,12Z) lysophosphatidylcholine
14:1(9Z)/22:2(13Z,16Z) phosphatidylethanolamine
1-oleoyl-lysophosphatidylserine
oleoylethanolamide

ethyl 3-hydroxytridecanoic acid
(all Z)-7,10,13,16,19-docosapentaenoic acid
prostaglandin D2
prostaglandin E2
dm18:1(9Z) lysoplasmenylcholine
lithocholic acid
lauroylcarnitine
prostaglandin C2
4-hydroxyatomoxetine
1-(1Z-hexadecenyl)-sn-glycero-3-phosphoethanolamine
tripropionin
11,12-DHET
protoporphyrinogen
1-palmitoyl-2-hydroxy-sn-glycero-3-phosphoethanolamine
pentadecanoic acid
1-16:0 lysophosphatidylcholine
1-20:2(11Z,14Z) lysophosphatidylcholine
goralatide
1-oleoyl-lysophosphatidylethanolamine
L-palmitoylcarnitine
3-tyramine
L-alpha-lysophosphatidylcholine, palmitoyl
beta-estradiol
18-oxocortisol
12(R)-hydroxyeicosatrienoic acid
sterol
9-hexadecenoylcarnitine
1-alpha,24(R),25-trihydroxyvitamin D3
dm16:0 lysoplasmenylcholine
N-arachidonylglycine
1-arachidonoylglycerol
estradiol-3-glucuronide
5beta-cholestane-3alpha,7alpha,12alpha,24r,25-pentol
dm18:0 lysoplasmenylcholine
1-15:0 lysophosphatidylcholine
2-18:2(9Z,12Z) lysophosphatidylethanolamine
oleoylcarnitine
arachidonic acid
cholest-5-ene
3-sulfodeoxycholic acid
1-1z-alkenyl-2-acylglycerophosphate
15:0/18:4(6Z,9Z,12Z,15Z) phosphatidylethanolamine
N-acetyl-L-tyrosine
vitamin A
Ile-Phe
1-stearoyl-lysophosphatidic acid
2-keto-4-methylthiobutyric acid

xanthine
acetyl phosphate
2-oxoglutaric acid
indole
12-oxolithocholic acid
D-alpha-D-digalacturonic acid
tetracosahexaenoic acid
D-carnitine
trihexosylceramide (d18:1/24:1(15Z))
tristearin
2-[3-carboxy-3-(methylammonio)propyl]-L-histidine
thiamine
13,16-docosadienoic acid
glutaric acid
epinephrine
N-formylmethionine
erythrose
beta-lactose
D-glyceraldehyde
alpha-D-galactose
24:0/24:0 phosphatidylcholine
5-phosphoribosylamine
20:5(5Z,8Z,11Z,14Z,17Z)/18:3(9Z,12Z,15Z)/20:5(5Z,8Z,11Z,14Z,17Z)[iso3] triacylglycerol
2-phenylacetamide
indole-3-lactic acid
elemicin
myrmicacin
10-undecenoic acid
L-threonine
3-oxododecanoate
ganglioside GA2 (d18:1/22:0)
succinylcarnitine
L-methionine
10-formyldihydrofolic acid
cytosine
leucine
desoxycorticosterone
S-(2-methylbutanoyl)-dihydrolipoamide
glycine
dihydroactinidiolide
D-gal alpha 1->6D-gal alpha 1->6D-glucose
raffinose
alanine
ecgonine methyl ester
azelaic acid
ecgonine
L-valine

6-oxopiperidine-2-carboxylic acid
octanoic acid
5-methoxytryptophol
N-acetylproline
S-(2-hydroxyethyl)glutathione
linoleic acid
3-carboxy-4-methyl-5-propyl-2-furanpropanoic acid
acetyl-L-carnitine
stearidonic acid
VPGPR enterostatin
estriol
glycogen
coproporphyrin I
gamma-glutamylalanine
glutarylcarnitine
GABA
7-hydroxy-6-methyl-8-ribityl lumazine
saccharopine
linolenic acid
propenoylcarnitine
1-20:5(5Z,8Z,11Z,14Z,17Z)/2-15:0 diacylglycerol
octadecane-1,18-dioic acid
17alpha,21-dihydroxypregnenolone
acetylcholine
D-serine
hydroxypropionylcarnitine
N,N'-dimethylarginine
2-hydroxy-ethinylestradiol
2-hydroxy methyl olanzapine
alpha-tocotrienol
2-linoleoylglycerol
dityrosine
pregnanetriolone
4-hydroxyhippuric acid
4-oxo-1-(3-pyridyl)-1-butanone
phenylpropanoic acid
acetylleucine
isoferulic acid
3-methylguanine
phenylpropionylglycine
tyrosine methyl ester
(-)-salsoline
Ile-Trp
Leu-Trp
1-20:1(11Z) lysophosphatidylethanolamine
L-phenylalanine
2-oxoarginine

valerylglycine
1-20:2 (11Z,14Z) lysophosphatidylethanolamine
carboxyaminoimidazole ribotide
9(S),12(S),13(S)-trihydroxy-10(E)-octadecenoic acid
phenylacetaldehyde
D-glutamic acid
3-methoxytyramine
palmitoleic acid
dodecanedioic acid
corticosterone
5-hydroxyindol-3-acetic acid
1-11-undecanedioic acid
undecanoic acid
4-(2-amino-3-hydroxyphenyl)-2,4-dioxobutanoic acid
butenylcarnitine
monomethylpropion
S-adenosyl-3-methylthiopropylamine
tetrahydrocurcumin
N1,N12-diacetylspermine
1-14:1(9Z) monoacylglycerol
D-erythro-C16-ceramide

Type	Expression	9-5Gy-d9
chemical - endogenous mammalian	7.278	HMDB0240
chemical - endogenous mammalian	5.83	HMDB0001
chemical - endogenous mammalian	5.451	HMDB0001
chemical - endogenous mammalian	5.244	HMDB0001
chemical - endogenous mammalian	4.647	HMDB0006
chemical - endogenous mammalian	4.563	HMDB0062
chemical - endogenous mammalian	4.546	HMDB0005
chemical - endogenous mammalian	4.5	HMDB0000
chemical - endogenous mammalian	4.273	HMDB0000
chemical - endogenous mammalian	4.215	HMDB0000
chemical - endogenous mammalian	3.896	HMDB0061
chemical - endogenous mammalian	3.892	HMDB0009
chemical - endogenous mammalian	3.817	HMDB0002
chemical - endogenous mammalian	3.768	HMDB0004
chemical - endogenous mammalian	3.764	HMDB0010
chemical - endogenous mammalian	3.504	HMDB0000
chemical - endogenous mammalian	3.504	HMDB0004
chemical - endogenous mammalian	3.503	HMDB0061
chemical - endogenous mammalian	3.453	HMDB0039
chemical - endogenous mammalian	3.453	HMDB0006
chemical - endogenous mammalian	3.435	HMDB0062
chemical - endogenous mammalian	3.369	HMDB0000
chemical - endogenous mammalian	3.285	HMDB0060
chemical - endogenous mammalian	3.274	HMDB0012
chemical - endogenous mammalian	3.22	HMDB0013
chemical - endogenous mammalian	3.068	HMDB0013
chemical - endogenous mammalian	3.057	HMDB0000
chemical - endogenous mammalian	3.055	HMDB0008
chemical - endogenous mammalian	3.004	HMDB0000
chemical - endogenous mammalian	2.952	HMDB0002
chemical - endogenous mammalian	2.913	HMDB0032
chemical - endogenous mammalian	2.902	HMDB0060
chemical - endogenous mammalian	2.867	HMDB0003
chemical - endogenous mammalian	2.841	HMDB0001
chemical - endogenous mammalian	2.752	HMDB0031
chemical - endogenous mammalian	2.706	HMDB0005
chemical - endogenous mammalian	2.685	HMDB0000
chemical - endogenous mammalian	2.576	HMDB0002
chemical - endogenous mammalian	2.576	HMDB0002
chemical - endogenous mammalian	2.55	HMDB0000
chemical - endogenous mammalian	2.542	HMDB0000
chemical - endogenous mammalian	2.494	HMDB0000
chemical - endogenous mammalian	2.45	HMDB0094
chemical - endogenous mammalian	2.396	HMDB0000

chemical - endogenous mammalian	2.343	HMDB0061
chemical - endogenous mammalian	2.239	HMDB0004
chemical - endogenous mammalian	2.237	HMDB0004
chemical - endogenous mammalian	2.217	HMDB0000
chemical - endogenous mammalian	2.163	HMDB0000
chemical - endogenous mammalian	2.101	HMDB0060
chemical - endogenous mammalian	2.089	HMDB0006
chemical - endogenous mammalian	2.075	HMDB0000
chemical - endogenous mammalian	2.029	HMDB0001
chemical - endogenous mammalian	2.025	HMDB0062
chemical - endogenous mammalian	1.973	HMDB0029
chemical - endogenous mammalian	1.955	HMDB0033
chemical - endogenous mammalian	1.931	HMDB0001
chemical - endogenous mammalian	1.93	HMDB0002
chemical - endogenous mammalian	1.912	HMDB0000
chemical - endogenous mammalian	1.899	HMDB0001
chemical - endogenous mammalian	1.871	HMDB0012
chemical - endogenous mammalian	1.853	HMDB0006
chemical - endogenous mammalian	1.843	HMDB0000
chemical - endogenous mammalian	1.825	HMDB0011
chemical - endogenous mammalian	1.806	HMDB0000
chemical - endogenous mammalian	1.771	HMDB0003
chemical - endogenous mammalian	1.764	HMDB0001
chemical - endogenous mammalian	1.735	HMDB0006
chemical - endogenous mammalian	1.705	HMDB0003
chemical - endogenous mammalian	1.7	HMDB0000
chemical - endogenous mammalian	1.605	HMDB0000
chemical - endogenous mammalian	1.604	HMDB0061
chemical - endogenous mammalian	1.575	HMDB0059
chemical - endogenous mammalian	1.507	HMDB0061
chemical - endogenous mammalian	1.491	HMDB0013
chemical - endogenous mammalian	1.488	HMDB0000
chemical - endogenous mammalian	1.466	HMDB0013
chemical - endogenous mammalian	1.427	HMDB0062
chemical - endogenous mammalian	1.409	HMDB0094
chemical - endogenous mammalian	1.399	HMDB0061
chemical - endogenous mammalian	1.386	HMDB0010
chemical - endogenous mammalian	1.376	HMDB0002
chemical - endogenous mammalian	1.344	HMDB0062
chemical - endogenous mammalian	1.312	HMDB0012
chemical - endogenous mammalian	1.279	HMDB0002
chemical - endogenous mammalian	1.175	HMDB0002
chemical - endogenous mammalian	1.158	HMDB0011
chemical - endogenous mammalian	1.077	HMDB0000
chemical - endogenous mammalian	0.955	HMDB0013
chemical - endogenous mammalian	0.95	HMDB0000
chemical - endogenous mammalian	-0.559	HMDB0000

chemical - endogenous mammalian	-0.568	HMDB0006
chemical - endogenous mammalian	-0.58	HMDB0013
chemical - endogenous mammalian	-0.589	HMDB0005
chemical - endogenous mammalian	-0.606	HMDB0000
chemical - endogenous mammalian	-0.638	HMDB0000
chemical - endogenous mammalian	-0.65	HMDB0060
chemical - endogenous mammalian	-0.659	HMDB0007
chemical - endogenous mammalian	-0.671	HMDB0010
chemical - endogenous mammalian	-0.704	HMDB0011
chemical - endogenous mammalian	-0.719	HMDB0011
chemical - endogenous mammalian	-0.724	HMDB0028
chemical - endogenous mammalian	-0.73	HMDB0006
chemical - endogenous mammalian	-0.753	HMDB0029
chemical - endogenous mammalian	-0.774	HMDB0011
chemical - endogenous mammalian	-0.778	HMDB0000
chemical - endogenous mammalian	-0.786	HMDB0126
chemical - endogenous mammalian	-0.788	HMDB0000
chemical - endogenous mammalian	-0.793	HMDB0010
chemical - endogenous mammalian	-0.794	HMDB0062
chemical - endogenous mammalian	-0.821	HMDB0006
chemical - endogenous mammalian	-0.851	HMDB0002
chemical - endogenous mammalian	-0.853	HMDB0000
chemical - endogenous mammalian	-0.858	HMDB0004
chemical - endogenous mammalian	-0.86	HMDB0011
chemical - endogenous mammalian	-0.861	HMDB0061
chemical - endogenous mammalian	-0.863	HMDB0061
chemical - endogenous mammalian	-0.89	HMDB0009
chemical - endogenous mammalian	-0.902	HMDB0006
chemical - endogenous mammalian	-0.907	HMDB0007
chemical - endogenous mammalian	-0.911	HMDB0007
chemical - endogenous mammalian	-0.929	HMDB0008
chemical - endogenous mammalian	-0.934	HMDB0007
chemical - endogenous mammalian	-0.945	HMDB0004
chemical - endogenous mammalian	-0.946	HMDB0009
chemical - endogenous mammalian	-0.959	
chemical - endogenous mammalian	-1.042	
chemical - endogenous mammalian	-1.046	
chemical - endogenous mammalian	-1.06	
chemical - endogenous mammalian	-1.066	
chemical - endogenous mammalian	-1.077	
chemical - endogenous mammalian	-1.089	
chemical - endogenous mammalian	-1.089	
chemical - endogenous mammalian	-1.111	
chemical - endogenous mammalian	-1.124	
chemical - endogenous mammalian	-1.126	
chemical - endogenous mammalian	-1.141	
chemical - endogenous mammalian	-1.145	

chemical - endogenous mammalian	-1.151
chemical - endogenous mammalian	-1.167
chemical - endogenous mammalian	-1.195
chemical - endogenous mammalian	-1.226
chemical - endogenous mammalian	-1.227
chemical - endogenous mammalian	-1.257
chemical - endogenous mammalian	-1.268
chemical - endogenous mammalian	-1.269
chemical - endogenous mammalian	-1.28
chemical - endogenous mammalian	-1.285
chemical - endogenous mammalian	-1.311
chemical - endogenous mammalian	-1.314
chemical - endogenous mammalian	-1.319
chemical - endogenous mammalian	-1.32
chemical - endogenous mammalian	-1.327
chemical - endogenous mammalian	-1.335
chemical - endogenous mammalian	-1.372
chemical - endogenous mammalian	-1.381
chemical - endogenous mammalian	-1.383
chemical - endogenous mammalian	-1.384
chemical - endogenous mammalian	-1.398
chemical - endogenous mammalian	-1.409
chemical - endogenous mammalian	-1.435
chemical - endogenous mammalian	-1.488
chemical - endogenous mammalian	-1.496
chemical - endogenous mammalian	-1.508
chemical - endogenous mammalian	-1.515
chemical - endogenous mammalian	-1.519
chemical - endogenous mammalian	-1.541
chemical - endogenous mammalian	-1.578
chemical - endogenous mammalian	-1.601
chemical - endogenous mammalian	-1.602
chemical - endogenous mammalian	-1.605
chemical - endogenous mammalian	-1.615
chemical - endogenous mammalian	-1.622
chemical - endogenous mammalian	-1.64
chemical - endogenous mammalian	-1.652
chemical - endogenous mammalian	-1.691
chemical - endogenous mammalian	-1.697
chemical - endogenous mammalian	-1.703
chemical - endogenous mammalian	-1.712
chemical - endogenous mammalian	-1.74
chemical - endogenous mammalian	-1.751
chemical - endogenous mammalian	-1.751
chemical - endogenous mammalian	-1.756
chemical - endogenous mammalian	-1.759
chemical - endogenous mammalian	-1.803

chemical - endogenous mammalian	-1.809
chemical - endogenous mammalian	-1.823
chemical - endogenous mammalian	-1.827
chemical - endogenous mammalian	-1.875
chemical - endogenous mammalian	-1.891
chemical - endogenous mammalian	-1.894
chemical - endogenous mammalian	-1.91
chemical - endogenous mammalian	-2.032
chemical - endogenous mammalian	-2.099
chemical - endogenous mammalian	-2.144
chemical - endogenous mammalian	-2.18
chemical - endogenous mammalian	-2.195
chemical - endogenous mammalian	-2.251
chemical - endogenous mammalian	-2.322
chemical - endogenous mammalian	-2.394
chemical - endogenous mammalian	-2.421
chemical - endogenous mammalian	-2.533
chemical - endogenous mammalian	-2.653
chemical - endogenous mammalian	-2.712
chemical - endogenous mammalian	-3.561
chemical - endogenous mammalian	-4.097

Name	Type
L-urobilin	chemical - endogenous mammalian
progesterone	chemical - endogenous mammalian
benzoic acid	chemical - endogenous mammalian
mesobilirubinogen	chemical - endogenous mammalian
20-carboxy-leukotriene B4	chemical - endogenous mammalian
4alpha-formyl-5alpha-cholesta-8,24-dien-3beta-ol	chemical - endogenous mammalian
eicosa-11Z, 14Z-dienoic acid	chemical - endogenous mammalian
tetrahydrocortisol	chemical - endogenous mammalian
hyodeoxycholic acid	chemical - endogenous mammalian
18-oxocortisol	chemical - endogenous mammalian
methyl palmitate	chemical - endogenous mammalian
20:5(5Z,8Z,11Z,14Z,17Z)/24:0 phosphatidylethanolamine	chemical - endogenous mammalian
adrenic acid	chemical - endogenous mammalian
D-urobilin	chemical - endogenous mammalian
1-18:3(9Z,12Z,15Z) lysophosphatidylcholine	chemical - endogenous mammalian
isolithocholic acid	chemical - endogenous mammalian
(11E)-9,10,13-trihydroxyoctadec-11-enoic acid	chemical - endogenous mammalian
13,16-docosadienoic acid	chemical - endogenous mammalian
5-cis,8-cis-tetradecadienoic acid	chemical - endogenous mammalian
24:5(n-3) fatty acid	chemical - endogenous mammalian
goralatide	chemical - endogenous mammalian
5,6,7,8-tetrahydrobiopterin	chemical - endogenous mammalian
prostaglandin C2	chemical - endogenous mammalian
C24:1 sulfatide	chemical - endogenous mammalian
sinapyl alcohol	chemical - endogenous mammalian
5-hydroxytryptophol glucuronide	chemical - endogenous mammalian
pelargonic acid	chemical - endogenous mammalian
22:2(13Z,16Z)/14:1(9Z) phosphatidylcholine	chemical - endogenous mammalian
(Z)-5-dodecenoic acid	chemical - endogenous mammalian
tridecanedioic acid	chemical - endogenous mammalian
tripropionin	chemical - endogenous mammalian
4-hydroxyatomoxetine	chemical - endogenous mammalian
3,7,12-trihydroxycholestan-26-al	chemical - endogenous mammalian
prostaglandin D2	chemical - endogenous mammalian
heptadecanal	chemical - endogenous mammalian
nitrolinoleate	chemical - endogenous mammalian
alpha-estradiol	chemical - endogenous mammalian
omega3-arachidonic acid	chemical - endogenous mammalian
8,9-DHET	chemical - endogenous mammalian
estriol	chemical - endogenous mammalian
thymidine	chemical - endogenous mammalian
pregnenolone	chemical - endogenous mammalian
N-acetylproline	chemical - endogenous mammalian
pentadecanoic acid	chemical - endogenous mammalian

3-hydroxypentadecanoic acid	chemical - endogenous mammalian
(12Z)-9,10-dihydroxyoctadec-12-enoic acid	chemical - endogenous mammalian
(12R,13S)-(9Z)-linoleic acid epoxide	chemical - endogenous mammalian
24,25-dihydroxyvitamin D3	chemical - endogenous mammalian
12-oxolithocholic acid	chemical - endogenous mammalian
sterol	chemical - endogenous mammalian
beta-D-galactopyranosyl-(1->3)-N-acetyl-D-glucosamine	chemical - endogenous mammalian
thymine	chemical - endogenous mammalian
2-hydroxyphenethylamine	chemical - endogenous mammalian
12(S)-hydroxyeicosatrienoic acid	chemical - endogenous mammalian
gamma-glutamyltryptophan	chemical - endogenous mammalian
alanopine	chemical - endogenous mammalian
zymosterone	chemical - endogenous mammalian
deoxycholic acid 3-glucuronide	chemical - endogenous mammalian
acetoacetic acid	chemical - endogenous mammalian
icosapent	chemical - endogenous mammalian
3,7-dihydroxy-5-cholestenoic acid	chemical - endogenous mammalian
ubiquinone Q2	chemical - endogenous mammalian
3-methyladipic acid	chemical - endogenous mammalian
2-[3-carboxy-3-(methylammonio)propyl]-L-histidine	chemical - endogenous mammalian
tetrahydrodeoxycorticosterone	chemical - endogenous mammalian
VPGPR enterostatin	chemical - endogenous mammalian
nicotinic acid	chemical - endogenous mammalian
3-hydroxyhippuric acid	chemical - endogenous mammalian
D-serine	chemical - endogenous mammalian
L-threonine	chemical - endogenous mammalian
2'-deoxyadenosine	chemical - endogenous mammalian
succinylcarnitine	chemical - endogenous mammalian
isohexyl alcohol	chemical - endogenous mammalian
3-carboxy-4-methyl-5-propyl-2-furanpropanoic acid	chemical - endogenous mammalian
2-(14,15-epoxyeicosatrienoyl)glycerol	chemical - endogenous mammalian
taurine	chemical - endogenous mammalian
hydroxypropionylcarnitine	chemical - endogenous mammalian
S-(2-hydroxyethyl)glutathione	chemical - endogenous mammalian
1-oleoylglycerol	chemical - endogenous mammalian
2-18:1(9Z) lysophosphatidylcholine	chemical - endogenous mammalian
(R)-3-hydroxy-hexadecanoic acid	chemical - endogenous mammalian
N1,N12-diacetylspermine	chemical - endogenous mammalian
saccharopine	chemical - endogenous mammalian
palmitoleic acid	chemical - endogenous mammalian
1-oleoyl lysophosphatidylcholine	chemical - endogenous mammalian
N-acetylcadaverine	chemical - endogenous mammalian
2-linoleoylglycerol	chemical - endogenous mammalian
linoleic acid	chemical - endogenous mammalian
dm18:0 lysoplasmenylcholine	chemical - endogenous mammalian
glycylleucine	chemical - endogenous mammalian
S-adenosyl-3-methylthiopropylamine	chemical - endogenous mammalian

isoputreanine	chemical - endogenous mammalian
glutarylcarnitine	chemical - endogenous mammalian
tetrahydrocurcumin	chemical - endogenous mammalian
L-methionine	chemical - endogenous mammalian
coproporphyrin I	chemical - endogenous mammalian
2-hydroxy methyl olanzapine	chemical - endogenous mammalian
1-16:1(9Z)/2-15:0 diacylglycerol	chemical - endogenous mammalian
1-16:0 lysophosphatidylcholine	chemical - endogenous mammalian
1-palmitoylglycerol	chemical - endogenous mammalian
2-18:0 lysophosphatidylethanolamine	chemical - endogenous mammalian
phenylalanylglycine	chemical - endogenous mammalian
carboxyaminoimidazole ribotide	chemical - endogenous mammalian
Val-Gln	chemical - endogenous mammalian
1-20:0 lysophosphatidylethanolamine	chemical - endogenous mammalian
cholic acid	chemical - endogenous mammalian
eriodictyol	chemical - endogenous mammalian
palmitic acid	chemical - endogenous mammalian
L-alpha-lysophosphatidylcholine, stearoyl	chemical - endogenous mammalian
platelet activating factor-C16	chemical - endogenous mammalian
2-hexaprenyl-3-methyl-6-methoxy-1,4-benzoquinone	chemical - endogenous mammalian
gondoic acid	chemical - endogenous mammalian
oleic acid	chemical - endogenous mammalian
I-urobilin	chemical - endogenous mammalian
C18:1 dihydroceramide	chemical - endogenous mammalian
2-18:0 lysophosphatidylinositol	chemical - endogenous mammalian
N-desethyloxybutynin	chemical - endogenous mammalian
18:0/16:0 phosphatidylinositol	chemical - endogenous mammalian
cholesterol eicosapentadenoate	chemical - endogenous mammalian
1-palmitoyl-2-arachidonoyl-phosphatidylcholine	chemical - endogenous mammalian
1-15:0/2-22:2(13Z,16Z) diacylglycerol	chemical - endogenous mammalian
14:1(9Z)/24:1(15Z) phosphatidylethanolamine	chemical - endogenous mammalian
14:0/20:2(11Z,14Z) phosphatidylcholine	chemical - endogenous mammalian
D-erythro-C16-ceramide	chemical - endogenous mammalian
16:0/20:3(5Z,8Z,11Z) phosphatidylinositol-3-phosphate	chemical - endogenous mammalian

Expression	11Gy-d1	Name
3.498	HMDB0007868	14:0/15:0 phosphatidylcholine
3.167	HMDB0240259	L-urobilin
3.127	HMDB0001898	mesobilirubinogen
2.655	HMDB0000054	bilirubin
2.546	HMDB0005060	eicosa-11Z, 14Z-dienoic acid
2.519	HMDB0004158	D-urobilinogen
2.392	HMDB0004161	D-urobilin
2.366	HMDB0062389	4alpha-formyl-5alpha-cholesta-8,24-dien-3beta-ol
2.174	HMDB0006888	5beta-cyprinolsulfate
2.053	HMDB0029765	9-trans-pentadecenoic acid
1.991	HMDB0062552	goralatide
1.924	HMDB0011563	1-pentadecanoylglycerol
1.916	HMDB0036620	5,7-dimethoxyflavone
1.908	HMDB0006059	20-carboxy-leukotriene B4
1.83	HMDB0001830	progesterone
1.825	HMDB0060095	prostaglandin C2
1.804	HMDB0001335	epoprostenol
1.756	HMDB0001403	prostaglandin D2
1.742	HMDB0006323	24:5(n-3) fatty acid
1.719	HMDB0004989	3-tyramine
1.704	HMDB0059889	isohexyl alcohol
1.691	HMDB0041884	doxorubicinol
1.658	HMDB0062251	alanine
1.64	HMDB0006485	10-formyldihydrofolic acid
1.632	HMDB0010727	3-oxododecanoate
1.596	HMDB0142963	2-amino-6-hydroxyhexanoic acid
1.578	HMDB0013335	3-hydroxyhexadecadienoylcarnitine
1.526	HMDB0000339	2-methylbutyrylglycine
1.522	HMDB0000251	taurine
1.522	HMDB0002234	1-pyrroline-4-hydroxy-2-carboxylate
1.516	HMDB0003339	D-glutamic acid
1.498	HMDB0006556	L-4-hydroxyglutamate semialdehyde
1.487	HMDB0000148	L-glutamic acid
1.486	HMDB0000512	N-acetyl-L-phenylalanine
1.479	HMDB0000860	phenylpropionylglycine
1.459	HMDB0059616	all-trans-decaprenyl diphosphate
1.447	HMDB0000735	4-hydroxyphenylacetylglycine
1.431	HMDB0030058	ethyl propionate
1.42	HMDB0005096	N-arachidonylglycine
1.406	HMDB0009003	1-stearoyl-2-arachidonoyl-sn-glycero-3-phosphoet
1.401		
1.384		
1.378		
1.355		

1.277
1.196
1.174
1.171
1.159
1.128
1.123
1.119
1.075
1.069
1.056
1.054
1.043
1.041
1.019
1.018
0.991
0.99
0.976
0.897
0.858
0.601
0.59
-0.955
-0.963
-0.991
-1.064
-1.079
-1.099
-1.115
-1.199
-1.2
-1.204
-1.21
-1.211
-1.244
-1.276
-1.282
-1.284
-1.346
-1.349
-1.363
-1.377
-1.421
-1.457
-1.459
-1.467

-1.472
-1.484
-1.516
-1.517
-1.558
-1.567
-1.6
-1.626
-1.662
-1.667
-1.698
-1.853
-1.881
-1.894
-1.994
-2.061
-2.147
-2.18
-2.18
-2.202
-2.26
-2.263
-2.317
-2.433
-2.644
-2.664
-2.854
-3.03
-3.427
-3.68
-3.701
-3.809
-4.015
-4.88

Type	Expression	11Gy-d3
chemical - endogenous mammalian	3.684	HMDB0005060
chemical - endogenous mammalian	2.985	HMDB0009931
chemical - endogenous mammalian	2.73	HMDB0002226
chemical - endogenous mammalian	2.694	HMDB0007868
chemical - endogenous mammalian	2.349	HMDB0060501
chemical - endogenous mammalian	2.329	HMDB0010378
chemical - endogenous mammalian	1.97	HMDB0006888
chemical - endogenous mammalian	1.915	HMDB0003128
chemical - endogenous mammalian	1.68	HMDB0240259
chemical - endogenous mammalian	1.674	HMDB0011572
chemical - endogenous mammalian	1.594	HMDB0001830
chemical - endogenous mammalian	1.574	HMDB0013200
chemical - endogenous mammalian	1.498	HMDB0006059
chemical - endogenous mammalian	1.411	HMDB0011563
chemical - endogenous mammalian	1.404	HMDB0005084
chemical - endogenous mammalian	1.322	HMDB0007069
chemical - endogenous mammalian	1.173	HMDB0011487
chemical - endogenous mammalian	1.173	HMDB0060469
chemical - endogenous mammalian	0.925	HMDB0034296
chemical - endogenous mammalian	0.916	HMDB0000827
chemical - endogenous mammalian	0.91	HMDB0010387
chemical - endogenous mammalian	-0.684	HMDB0062297
chemical - endogenous mammalian	-0.911	HMDB0007121
chemical - endogenous mammalian	-0.914	HMDB0061694
chemical - endogenous mammalian	-0.934	HMDB0031039
chemical - endogenous mammalian	-0.966	HMDB0002982
chemical - endogenous mammalian	-0.981	HMDB0010331
chemical - endogenous mammalian	-1.028	HMDB0001551
chemical - endogenous mammalian	-1.229	HMDB0001898
chemical - endogenous mammalian	-1.238	HMDB0062195
chemical - endogenous mammalian	-1.238	HMDB0006210
chemical - endogenous mammalian	-1.238	HMDB0000878
chemical - endogenous mammalian	-1.238	HMDB0001220
chemical - endogenous mammalian	-1.391	HMDB0011149
chemical - endogenous mammalian	-1.391	HMDB0010390
chemical - endogenous mammalian	-1.487	HMDB0013622
chemical - endogenous mammalian	-1.603	HMDB0004989
chemical - endogenous mammalian	-1.604	HMDB0006319
chemical - endogenous mammalian	-1.906	HMDB0060095
chemical - endogenous mammalian	-2.542	HMDB0000054
		HMDB0006709
		HMDB0062552
		HMDB0002088
		HMDB0002311

HMDB0001976
HMDB0007827
HMDB0006335
HMDB0061859
HMDB0011148
HMDB0000277
HMDB0011511
HMDB0062290
HMDB0011578
HMDB0010408
HMDB0062690
HMDB0010382
HMDB0062541
HMDB0007921
HMDB0061657
HMDB0000220
HMDB0000826
HMDB0002250
HMDB0060766
HMDB0032857
HMDB0006224
HMDB0010392
HMDB0003141
HMDB0000761
HMDB0062389
HMDB0036620
HMDB0002504
HMDB0006228
HMDB0000556
HMDB0006323
HMDB0010407
HMDB0010381
HMDB0013122
HMDB0028933
HMDB0011506
HMDB0060512
HMDB0062680
HMDB0000222
HMDB0008898
HMDB0094684
HMDB0000866
HMDB0000308
HMDB0011155
HMDB0000518
HMDB0001542
HMDB0000167
HMDB0000305

HMDB0033747
HMDB0004883
HMDB0005393
HMDB0008782
HMDB0001140
HMDB0000630
HMDB0001051
HMDB0033858
HMDB0004893
HMDB0031526
HMDB0061714
HMDB0033778
HMDB0062251
HMDB0062525
HMDB0062555
HMDB0003411
HMDB0001128
HMDB0000661
HMDB0006548
HMDB0010539
HMDB0002007
HMDB0002649
HMDB0002203
HMDB0000122
HMDB0006050
HMDB0000158
HMDB0003577
HMDB0002832
HMDB0062634
HMDB0006116
HMDB0060959
HMDB0006406
HMDB0003355
HMDB0062203
HMDB0000673
HMDB0013129
HMDB0000123
HMDB0006867
HMDB0061112
HMDB0033458
HMDB0000824
HMDB0006762
HMDB0007081
HMDB0000201
HMDB0000757
HMDB0012469
HMDB0011512

HMDB0001896
HMDB0000477
HMDB0000929
HMDB0000205
HMDB0001931
HMDB0006485
HMDB0000806
HMDB0000643
HMDB0006940
HMDB0001161
HMDB0000640
HMDB0013124
HMDB0002014
HMDB0062263
HMDB0011538
HMDB0060684
HMDB0007561
HMDB0001513
HMDB0013130
HMDB0000860
HMDB0000227
HMDB0013127
HMDB0060987
HMDB0000782
HMDB0006101
HMDB0000339
HMDB0001388
HMDB0000832
HMDB0003654
HMDB0000413
HMDB0000744
HMDB0011756
HMDB0006944
HMDB0000394
HMDB0000959
HMDB0000955
HMDB0000930
HMDB0031057
HMDB0000897
HMDB0000847
HMDB0012884
HMDB0011513
HMDB0000424
HMDB0126018
HMDB0012328
HMDB0000482
HMDB0000186

HMDB0006547
HMDB0000784
HMDB0029217
HMDB0003180
HMDB0035240
HMDB0000715
HMDB0060026
HMDB0001547
HMDB0004710
HMDB0000022
HMDB0004225
HMDB0002199
HMDB0002172
HMDB0062406
HMDB0000159
HMDB0003339
HMDB0041927
HMDB0005789
HMDB0000988
HMDB0003334
HMDB0000792
HMDB0000888
HMDB0004083
HMDB0040463
HMDB0000763
HMDB0006236
HMDB0000947
HMDB0013126
HMDB0000623
HMDB0011562

Name
eicosa-11Z, 14Z-dienoic acid
16:0/20:4(5Z,8Z,11Z,14Z) phosphatidylinositol-3-phosphate
adrenic acid
14:0/15:0 phosphatidylcholine
phosphatidylethanolamine
mead acid
5beta-cyprinolsulfate
cortolone
L-urobilin
1-arachidonylglycerol
progesterone
5-hydroxytryptophol glucuronide
20-carboxy-leukotriene B4
1-pentadecanoylglycerol
N-acetylleukotriene e4
1-15:0/2-16:0 diacylglycerol
2-20:4(5Z,8Z,11Z,14Z) lysophosphatidylethanolamine
dehydrodolichol diphosphate
11-eicosenoic acid
stearic acid
1-18:3(6Z,9Z,12Z) lysophosphatidylcholine
prostaglandin D2
1-16:0/2-22:6(4Z,7Z,10Z,13Z,16Z,19Z) diacylglycerol
1-oleoyl-lysophosphatidylserine
heptadecanal
prostaglandin B1
beta-D-glucuronoside
hexadecanal
mesobilirubinogen
platelet activating factor-C16
heptadecanoylcarnitine
ergosterol
prostaglandin E2
1-alkyl-2-lyso-sn-glycero-3-phosphocholine
1-20:0 lysophosphatidylcholine
nonadeca-10(Z)-enoic acid
3-tyramine
alpha-linolenyl carnitine
prostaglandin C2
bilirubin
ubiquinone Q2
goralatide
oleoylethanolamide
8,9-DHET

cis-4,7,10,13,16-docosapentaenoic acid
1-24:1(15Z)/2-18:1(9Z) diacylglycerol
beta-tocopherol
methyl palmitate
1-alkyl-2-acetyl-sn-glycero-3-phosphocholine
sphingosine-1-phosphate
1-20:0 lysophosphatidylethanolamine
12(R)-hydroxyeicosatrienoic acid
1-arachidonoylglycerol
dm18:1(9Z) lysoplasmenylcholine
1,2-oleoylphosphatidylcholine
1-16:0 lysophosphatidylcholine
L-alpha-lysophosphatidylcholine, palmitoyl
14:1(9Z)/22:2(13Z,16Z) phosphatidylcholine
3-hydroxypentadecanoic acid
palmitic acid
pentadecanoic acid
lauroylcarnitine
4-hydroxyatomoxetine
tripropionin
estradiol-3-glucuronide
1-20:2(11Z,14Z) lysophosphatidylcholine
retinoyl glucuronide
lithocholic acid
4alpha-formyl-5alpha-cholesta-8,24-dien-3beta-ol
5,7-dimethoxyflavone
3-sulfodeoxycholic acid
1-alpha,24(R),25-trihydroxyvitamin D3
5beta-cholestane-3alpha,7alpha,12alpha,24r,25-pentol
24:5(n-3) fatty acid
dm16:0 lysoplasmenylcholine
1-15:0 lysophosphatidylcholine
dm18:0 lysoplasmenylcholine
Leu-Leu
1-oleoyl-lysophosphatidylethanolamine
sterol
3-oxocholic acid
L-palmitoylcarnitine
15:0/18:4(6Z,9Z,12Z,15Z) phosphatidylethanolamine
1-oleoylglycerol
N-acetyl-L-tyrosine
3beta-hydroxy-5-cholenoic acid
1-1z-alkenyl-2-acylglycerophosphate
chenodeoxycholic acid
N-acetyllactosamine
L-threonine
vitamin A

alanopine
trihexosylceramide (d18:1/24:1(15Z))
tristearin
24:0/24:0 phosphatidylcholine
octanal
cytosine
D-glyceraldehyde
crotonyl alcohol
ganglioside GA2 (d18:1/22:0)
2-methylbutanal
13,16-docosadienoic acid
elemicin
alanine
S-(2-hydroxyethyl)glutathione
hydroxyisovaleryl carnitine
D-proline
5-phosphoribosylamine
glutaric acid
ecgonine
20:5(5Z,8Z,11Z,14Z,17Z)/18:3(9Z,12Z,15Z)/20:5(5Z,8Z,11Z,14Z,17Z)[iso3] triacylglyce
tetracosahexaenoic acid
erythrose
myrmicacin
D-glucose
2-tyrosine
L-tyrosine
VPGPR enterostatin
nordefrin
D-carnitine
3-hydroxyhippuric acid
desmethyl olanzapine
ecgonine methyl ester
5-aminovaleric acid
leucine
linoleic acid
glutaconylcarnitine
glycine
S-3-methylbutanoyl-dihydrolipoamide
3-carboxy-4-methyl-5-propyl-2-furanpropanoic acid
N-carbamoylputrescine
propionyl-L-carnitine
17alpha,21-dihydroxypregnenolone
1-15:0/2-20:3(5Z,8Z,11Z) diacylglycerol
acetyl-L-carnitine
glycogen
(-)-salsoline
1-20:1(11Z) lysophosphatidylethanolamine

5-methoxytryptophol
7Z,10Z-hexadecadienoic acid
L-tryptophan
phenylpyruvic acid
2,7,8-trimethyl-2-(beta-carboxyethyl)-6-hydroxychroman
10-formyldihydrofolic acid
myristic acid
coproporphyrin I
9(S)-HPODE
gamma-butyrobetaine
glucosan
propenoylcarnitine
cis-5-tetradecenoylcarnitine
L-serine
2-linoleoylglycerol
2-propylglutaric acid
1-20:5(5Z,8Z,11Z,14Z,17Z)/2-15:0 diacylglycerol
dolichyl diphosphate
glutarylcarnitine
phenylpropionylglycine
mevalonic acid
hydroxybutyrylcarnitine
2-hydroxy methyl olanzapine
octadecane-1,18-dioic acid
enterolactone
2-methylbutyrylglycine
linolenic acid
capryloylglycine
3-(4-hydroxyphenyl)-1-propane
3-hydroxydodecanedioic acid
malic acid
acetylleucine
cellulose
3-hydroxytetradecanedioic acid
tiglylglycine
isoferulic acid
trans-cinnamic acid
2-hydroxyhexadecanoic acid
7-methylguanine
pelargonic acid
adrenochrome
1-20:2 (11Z,14Z) lysophosphatidylethanolamine
2-hydroxysebacic acid
eriodictyol
palmitoleic acid
octanoic acid
lactose

stearidonic acid
azelaic acid
tyrosine methyl ester
cortol
dihydroactinidiolide
kynurenic acid
N-vanilloylglycine
corticosterone
(11E)-9,10,13-trihydroxyoctadec-11-enoic acid
3-methoxytyramine
2-oxoarginine
phloretic acid
N1,N12-diacetylspermine
4-oxo-1-(3-pyridyl)-1-butanone
L-phenylalanine
D-glutamic acid
monomethylpropion
tetrahydrocurcumin
S-adenosyl-3-methylthiopropylamine
N,N'-dimethylarginine
sebacic acid
1-11-undecanedioic acid
4-(2-amino-3-hydroxyphenyl)-2,4-dioxobutanoic acid
ethyl sorbate
5-hydroxyindol-3-acetic acid
phenylacetaldehyde
undecanoic acid
butenylcarnitine
dodecanedioic acid
1-14:1(9Z) monoacylglycerol

Type	Expression	11Gy-d9
chemical - endogenous mammalian	5.808	HMDB0005060
chemical - endogenous mammalian	5.385	HMDB0006059
chemical - endogenous mammalian	5.309	HMDB0000054
chemical - endogenous mammalian	5.016	HMDB0010390
chemical - endogenous mammalian	4.69	HMDB0061704
chemical - endogenous mammalian	4.684	HMDB0002226
chemical - endogenous mammalian	4.656	HMDB0010381
chemical - endogenous mammalian	4.372	HMDB0011148
chemical - endogenous mammalian	4.335	HMDB0011563
chemical - endogenous mammalian	4.331	HMDB0062195
chemical - endogenous mammalian	4.274	HMDB0240259
chemical - endogenous mammalian	4.208	HMDB0010387
chemical - endogenous mammalian	4.102	HMDB0000430
chemical - endogenous mammalian	4.091	HMDB0000289
chemical - endogenous mammalian	3.975	HMDB0011511
chemical - endogenous mammalian	3.814	HMDB0005943
chemical - endogenous mammalian	3.769	HMDB0001403
chemical - endogenous mammalian	3.737	HMDB0013200
chemical - endogenous mammalian	3.578	HMDB0000306
chemical - endogenous mammalian	3.548	HMDB0011506
chemical - endogenous mammalian	3.443	HMDB0005096
chemical - endogenous mammalian	3.28	HMDB0005065
chemical - endogenous mammalian	3.263	HMDB0002314
chemical - endogenous mammalian	3.222	HMDB0010392
chemical - endogenous mammalian	3.137	HMDB0010378
chemical - endogenous mammalian	3.108	HMDB0006319
chemical - endogenous mammalian	3.101	HMDB0031039
chemical - endogenous mammalian	3.003	HMDB0000744
chemical - endogenous mammalian	2.992	HMDB0000222
chemical - endogenous mammalian	2.944	HMDB0001830
chemical - endogenous mammalian	2.934	HMDB0061661
chemical - endogenous mammalian	2.909	HMDB0007855
chemical - endogenous mammalian	2.906	HMDB0060095
chemical - endogenous mammalian	2.878	HMDB0004949
chemical - endogenous mammalian	2.863	HMDB0003034
chemical - endogenous mammalian	2.832	HMDB0013122
chemical - endogenous mammalian	2.822	HMDB0001898
chemical - endogenous mammalian	2.78	HMDB0001542
chemical - endogenous mammalian	2.768	HMDB0062552
chemical - endogenous mammalian	2.767	HMDB0000230
chemical - endogenous mammalian	2.724	HMDB0000112
chemical - endogenous mammalian	2.686	HMDB0013631
chemical - endogenous mammalian	2.68	HMDB0003339
chemical - endogenous mammalian	2.573	HMDB0000751

chemical - endogenous mammalian	2.499	HMDB0000866
chemical - endogenous mammalian	2.492	HMDB0034296
chemical - endogenous mammalian	2.469	HMDB0006323
chemical - endogenous mammalian	2.353	HMDB0000167
chemical - endogenous mammalian	2.247	HMDB0000043
chemical - endogenous mammalian	2.243	HMDB0001138
chemical - endogenous mammalian	2.24	HMDB0007854
chemical - endogenous mammalian	2.166	HMDB0000060
chemical - endogenous mammalian	2.159	HMDB0001080
chemical - endogenous mammalian	2.09	HMDB0003411
chemical - endogenous mammalian	2.059	HMDB0000959
chemical - endogenous mammalian	2.055	HMDB0001140
chemical - endogenous mammalian	2.011	HMDB0031526
chemical - endogenous mammalian	1.996	HMDB0006478
chemical - endogenous mammalian	1.941	HMDB0001318
chemical - endogenous mammalian	1.928	HMDB0002203
chemical - endogenous mammalian	1.892	HMDB0001488
chemical - endogenous mammalian	1.873	HMDB0010325
chemical - endogenous mammalian	1.78	HMDB0062525
chemical - endogenous mammalian	1.766	HMDB0033724
chemical - endogenous mammalian	1.75	HMDB0001093
chemical - endogenous mammalian	1.675	HMDB0000927
chemical - endogenous mammalian	1.602	HMDB0006116
chemical - endogenous mammalian	1.569	HMDB0013070
chemical - endogenous mammalian	1.54	HMDB0013651
chemical - endogenous mammalian	1.533	HMDB0000201
chemical - endogenous mammalian	1.531	HMDB0003577
chemical - endogenous mammalian	1.489	HMDB0013333
chemical - endogenous mammalian	1.445	HMDB0013824
chemical - endogenous mammalian	1.443	HMDB0000394
chemical - endogenous mammalian	1.441	HMDB0240253
chemical - endogenous mammalian	1.435	HMDB0012469
chemical - endogenous mammalian	1.4	HMDB0011756
chemical - endogenous mammalian	1.348	HMDB0029160
chemical - endogenous mammalian	1.308	HMDB0000253
chemical - endogenous mammalian	1.281	HMDB0000735
chemical - endogenous mammalian	1.258	HMDB0000122
chemical - endogenous mammalian	1.256	HMDB0000860
chemical - endogenous mammalian	1.239	HMDB0000207
chemical - endogenous mammalian	1.227	HMDB0011512
chemical - endogenous mammalian	1.215	HMDB0000673
chemical - endogenous mammalian	1.123	HMDB0011538
chemical - endogenous mammalian	1.113	HMDB0000733
chemical - endogenous mammalian	1.098	HMDB0060102
chemical - endogenous mammalian	0.972	HMDB0003334
chemical - endogenous mammalian	0.929	HMDB0004225
chemical - endogenous mammalian	0.8	HMDB0000159

chemical - endogenous mammalian	0.74	HMDB0011517
chemical - endogenous mammalian	-0.563	HMDB0002007
chemical - endogenous mammalian	-0.602	HMDB0062680
chemical - endogenous mammalian	-0.659	HMDB0062406
chemical - endogenous mammalian	-0.66	HMDB0062698
chemical - endogenous mammalian	-0.73	HMDB0035514
chemical - endogenous mammalian	-0.751	HMDB0006547
chemical - endogenous mammalian	-0.762	HMDB0006228
chemical - endogenous mammalian	-0.768	HMDB0006273
chemical - endogenous mammalian	-0.769	HMDB0060038
chemical - endogenous mammalian	-0.774	HMDB0060987
chemical - endogenous mammalian	-0.797	HMDB0000978
chemical - endogenous mammalian	-0.819	HMDB0000715
chemical - endogenous mammalian	-0.844	HMDB0000158
chemical - endogenous mammalian	-0.862	HMDB0000714
chemical - endogenous mammalian	-0.87	HMDB0006101
chemical - endogenous mammalian	-0.891	HMDB0001547
chemical - endogenous mammalian	-0.904	HMDB0041700
chemical - endogenous mammalian	-0.947	HMDB0001412
chemical - endogenous mammalian	-0.956	HMDB0000895
chemical - endogenous mammalian	-0.972	HMDB0013126
chemical - endogenous mammalian	-0.973	HMDB0000477
chemical - endogenous mammalian	-0.985	HMDB0000806
chemical - endogenous mammalian	-0.988	HMDB0013125
chemical - endogenous mammalian	-0.997	HMDB0009410
chemical - endogenous mammalian	-0.997	HMDB0061717
chemical - endogenous mammalian	-1.014	HMDB0000643
chemical - endogenous mammalian	-1.015	HMDB0032857
chemical - endogenous mammalian	-1.019	HMDB0060026
chemical - endogenous mammalian	-1.021	HMDB0007921
chemical - endogenous mammalian	-1.026	HMDB0061112
chemical - endogenous mammalian	-1.056	HMDB0013127
chemical - endogenous mammalian	-1.07	HMDB0000696
chemical - endogenous mammalian	-1.08	HMDB0013130
chemical - endogenous mammalian	-1.089	HMDB0011651
chemical - endogenous mammalian	-1.112	HMDB0004702
chemical - endogenous mammalian	-1.138	HMDB0001388
chemical - endogenous mammalian	-1.145	HMDB0000988
chemical - endogenous mammalian	-1.161	HMDB0008654
chemical - endogenous mammalian	-1.179	HMDB0000951
chemical - endogenous mammalian	-1.18	HMDB0005789
chemical - endogenous mammalian	-1.192	HMDB0007790
chemical - endogenous mammalian	-1.203	HMDB0012328
chemical - endogenous mammalian	-1.235	HMDB0061042
chemical - endogenous mammalian	-1.249	HMDB0000593
chemical - endogenous mammalian	-1.251	HMDB0009929
chemical - endogenous mammalian	-1.257	

chemical - endogenous mammalian	-1.262
chemical - endogenous mammalian	-1.272
chemical - endogenous mammalian	-1.278
chemical - endogenous mammalian	-1.293
chemical - endogenous mammalian	-1.301
chemical - endogenous mammalian	-1.317
chemical - endogenous mammalian	-1.352
chemical - endogenous mammalian	-1.37
chemical - endogenous mammalian	-1.377
chemical - endogenous mammalian	-1.416
chemical - endogenous mammalian	-1.418
chemical - endogenous mammalian	-1.428
chemical - endogenous mammalian	-1.431
chemical - endogenous mammalian	-1.445
chemical - endogenous mammalian	-1.452
chemical - endogenous mammalian	-1.453
chemical - endogenous mammalian	-1.475
chemical - endogenous mammalian	-1.487
chemical - endogenous mammalian	-1.509
chemical - endogenous mammalian	-1.561
chemical - endogenous mammalian	-1.572
chemical - endogenous mammalian	-1.573
chemical - endogenous mammalian	-1.582
chemical - endogenous mammalian	-1.585
chemical - endogenous mammalian	-1.586
chemical - endogenous mammalian	-1.64
chemical - endogenous mammalian	-1.686
chemical - endogenous mammalian	-1.707
chemical - endogenous mammalian	-1.716
chemical - endogenous mammalian	-1.721
chemical - endogenous mammalian	-1.727
chemical - endogenous mammalian	-1.735
chemical - endogenous mammalian	-1.749
chemical - endogenous mammalian	-1.774
chemical - endogenous mammalian	-1.819
chemical - endogenous mammalian	-1.82
chemical - endogenous mammalian	-1.82
chemical - endogenous mammalian	-1.821
chemical - endogenous mammalian	-1.885
chemical - endogenous mammalian	-1.886
chemical - endogenous mammalian	-1.917
chemical - endogenous mammalian	-1.92
chemical - endogenous mammalian	-1.926
chemical - endogenous mammalian	-1.942
chemical - endogenous mammalian	-1.987
chemical - endogenous mammalian	-2.002
chemical - endogenous mammalian	-2.014

chemical - endogenous mammalian	-2.036
chemical - endogenous mammalian	-2.067
chemical - endogenous mammalian	-2.097
chemical - endogenous mammalian	-2.102
chemical - endogenous mammalian	-2.126
chemical - endogenous mammalian	-2.138
chemical - endogenous mammalian	-2.173
chemical - endogenous mammalian	-2.174
chemical - endogenous mammalian	-2.176
chemical - endogenous mammalian	-2.241
chemical - endogenous mammalian	-2.279
chemical - endogenous mammalian	-2.435
chemical - endogenous mammalian	-2.445
chemical - endogenous mammalian	-2.534
chemical - endogenous mammalian	-2.545
chemical - endogenous mammalian	-2.551
chemical - endogenous mammalian	-2.7
chemical - endogenous mammalian	-2.703
chemical - endogenous mammalian	-2.714
chemical - endogenous mammalian	-2.782
chemical - endogenous mammalian	-2.844
chemical - endogenous mammalian	-2.916
chemical - endogenous mammalian	-2.995
chemical - endogenous mammalian	-3.027
chemical - endogenous mammalian	-3.226
chemical - endogenous mammalian	-3.242
chemical - endogenous mammalian	-3.652
chemical - endogenous mammalian	-4.345
chemical - endogenous mammalian	-4.495
chemical - endogenous mammalian	-4.709

Name
eicosa-11Z, 14Z-dienoic acid
20-carboxy-leukotriene B4
bilirubin
1-20:0 lysophosphatidylcholine
2-18:0 lysophosphatidylinositol
adrenic acid
1-15:0 lysophosphatidylcholine
1-alkyl-2-acetyl-sn-glycero-3-phosphocholine
1-pentadecanoylglycerol
platelet activating factor-C16
L-urobilin
1-18:3(6Z,9Z,12Z) lysophosphatidylcholine
24,25-dihydroxyvitamin D3
uric acid
1-20:0 lysophosphatidylethanolamine
pregnane
prostaglandin D2
5-hydroxytryptophol glucuronide
tyramine
1-oleoyl-lysophosphatidylethanolamine
N-arachidonylglycine
oleoylcarnitine
11,12-DHET
1-20:2(11Z,14Z) lysophosphatidylcholine
mead acid
alpha-linolenyl carnitine
heptadecanal
malic acid
L-palmitoylcarnitine
progesterone
9-hydroxystearic acid
monooleylphosphatidic acid
prostaglandin C2
D-erythro-C16-ceramide
prostaglandin D3
dm18:0 lysoplasmenylcholine
mesobilirubinogen
N-acetyllactosamine
goralatide
N-acetylneuraminic acid
GABA
(Z)-N-(1-oxo-9-octadecenyl)glycine
D-glutamic acid
L-xylulose

N-acetyl-L-tyrosine
11-eicosenoic acid
24:5(n-3) fatty acid
L-threonine
betaine
N-acetyl-L-glutamate
1-stearoyl-lysophosphatidic acid
acetoacetic acid
4-aminobutanal
D-proline
tiglylglycine
octanal
2-methylbutanal
isovaleraldehyde
isocaproaldehyde
myrmicacin
nicotinic acid
ethyl glucuronide
S-(2-hydroxyethyl)glutathione
10-undecenoic acid
zymosterone
valerylglycine
3-hydroxyhippuric acid
sinapyl alcohol
2-(14,15-epoxyeicosatrienoyl)glycerol
acetyl-L-carnitine
VPGPR enterostatin
3-hydroxy-9-hexadecenoylcarnitine
2-pentylfuran
3-hydroxytetradecanedioic acid
acetyltaurine
(-)-salsoline
acetylleucine
gamma-glutamyltryptophan
pregnenolone
4-hydroxyphenylacetylglycine
D-glucose
phenylpropionylglycine
oleic acid
1-20:1(11Z) lysophosphatidylethanolamine
linoleic acid
2-linoleoylglycerol
hyodeoxycholic acid
arachidonic acid
N,N'-dimethylarginine
2-oxoarginine
L-phenylalanine

1-arachidonoyl-2-hydroxy-sn-glycero-3-phosphoethanolamine
tetracosahexaenoic acid
3-oxocholic acid
4-oxo-1-(3-pyridyl)-1-butanone
saccharopine
2-oxindole-3-acetic acid
stearidonic acid
1-alpha,24(R),25-trihydroxyvitamin D3
carboxyaminoimidazole ribotide
cis-10-heptadecenoic acid
2-hydroxy methyl olanzapine
4-(2-aminophenyl)-2,4-dioxobutanoate
kynurenic acid
L-tyrosine
hippuric acid
enterolactone
corticosterone
6-hydroxy-enterolactone
dihydropteroate
acetylcholine
butenylcarnitine
7Z,10Z-hexadecadienoic acid
myristic acid
hydroxypropionylcarnitine
20:4(5Z,8Z,11Z,14Z)/24:1(15Z) phosphatidylethanolamine
succinylcarnitine
coproporphyrin I
tripropionin
N-vanilloylglycine
14:1(9Z)/22:2(13Z,16Z) phosphatidylcholine
3-carboxy-4-methyl-5-propyl-2-furanpropanoic acid
hydroxybutyrylcarnitine
L-methionine
glutarylcarnitine
11beta,20-dihydroxy-3-oxopregn-4-en-21-oic acid
(12R,13S)-(9Z)-linoleic acid epoxide
linolenic acid
S-adenosyl-3-methylthiopropylamine
22:4(7Z,10Z,13Z,16Z)/dm18:1(11Z) phosphatidylcholine
taurochenodeoxycholate
tetrahydrocurcumin
1-22:6(4Z,7Z,10Z,13Z,16Z,19Z)/2-24:1(15Z) diacylglycerol
palmitoleic acid
N-desethyloxybutynin
1,2-oleoylphosphatidylcholine
16:0/20:3(5Z,8Z,11Z) phosphatidylinositol-3-phosphate

Type	Expression
chemical - endogenous mammalian	3.937
chemical - endogenous mammalian	3.717
chemical - endogenous mammalian	3.468
chemical - endogenous mammalian	2.834
chemical - endogenous mammalian	2.813
chemical - endogenous mammalian	2.718
chemical - endogenous mammalian	2.71
chemical - endogenous mammalian	2.643
chemical - endogenous mammalian	2.576
chemical - endogenous mammalian	2.485
chemical - endogenous mammalian	2.361
chemical - endogenous mammalian	2.301
chemical - endogenous mammalian	2.227
chemical - endogenous mammalian	2.225
chemical - endogenous mammalian	2.198
chemical - endogenous mammalian	2.112
chemical - endogenous mammalian	2.026
chemical - endogenous mammalian	2.023
chemical - endogenous mammalian	1.997
chemical - endogenous mammalian	1.951
chemical - endogenous mammalian	1.938
chemical - endogenous mammalian	1.928
chemical - endogenous mammalian	1.902
chemical - endogenous mammalian	1.85
chemical - endogenous mammalian	1.838
chemical - endogenous mammalian	1.801
chemical - endogenous mammalian	1.764
chemical - endogenous mammalian	1.687
chemical - endogenous mammalian	1.67
chemical - endogenous mammalian	1.623
chemical - endogenous mammalian	1.563
chemical - endogenous mammalian	1.506
chemical - endogenous mammalian	1.49
chemical - endogenous mammalian	1.467
chemical - endogenous mammalian	1.465
chemical - endogenous mammalian	1.448
chemical - endogenous mammalian	1.443
chemical - endogenous mammalian	1.377
chemical - endogenous mammalian	1.351
chemical - endogenous mammalian	1.334
chemical - endogenous mammalian	1.33
chemical - endogenous mammalian	1.283
chemical - endogenous mammalian	1.162
chemical - endogenous mammalian	1.152

chemical - endogenous mammalian	1.083
chemical - endogenous mammalian	1.061
chemical - endogenous mammalian	1.031
chemical - endogenous mammalian	0.965
chemical - endogenous mammalian	0.949
chemical - endogenous mammalian	0.885
chemical - endogenous mammalian	0.883
chemical - endogenous mammalian	0.778
chemical - endogenous mammalian	0.728
chemical - endogenous mammalian	0.722
chemical - endogenous mammalian	0.698
chemical - endogenous mammalian	-0.645
chemical - endogenous mammalian	-0.664
chemical - endogenous mammalian	-0.664
chemical - endogenous mammalian	-0.691
chemical - endogenous mammalian	-0.709
chemical - endogenous mammalian	-0.815
chemical - endogenous mammalian	-0.821
chemical - endogenous mammalian	-0.863
chemical - endogenous mammalian	-0.867
chemical - endogenous mammalian	-0.942
chemical - endogenous mammalian	-0.951
chemical - endogenous mammalian	-0.968
chemical - endogenous mammalian	-1.014
chemical - endogenous mammalian	-1.025
chemical - endogenous mammalian	-1.048
chemical - endogenous mammalian	-1.066
chemical - endogenous mammalian	-1.09
chemical - endogenous mammalian	-1.115
chemical - endogenous mammalian	-1.126
chemical - endogenous mammalian	-1.129
chemical - endogenous mammalian	-1.139
chemical - endogenous mammalian	-1.225
chemical - endogenous mammalian	-1.234
chemical - endogenous mammalian	-1.279
chemical - endogenous mammalian	-1.284
chemical - endogenous mammalian	-1.29
chemical - endogenous mammalian	-1.297
chemical - endogenous mammalian	-1.298
chemical - endogenous mammalian	-1.299
chemical - endogenous mammalian	-1.325
chemical - endogenous mammalian	-1.334
chemical - endogenous mammalian	-1.343
chemical - endogenous mammalian	-1.349
chemical - endogenous mammalian	-1.421
chemical - endogenous mammalian	-1.484
chemical - endogenous mammalian	-1.513

chemical - endogenous mammalian	-1.519
chemical - endogenous mammalian	-1.529
chemical - endogenous mammalian	-1.538
chemical - endogenous mammalian	-1.579
chemical - endogenous mammalian	-1.606
chemical - endogenous mammalian	-1.627
chemical - endogenous mammalian	-1.645
chemical - endogenous mammalian	-1.675
chemical - endogenous mammalian	-1.68
chemical - endogenous mammalian	-1.687
chemical - endogenous mammalian	-1.689
chemical - endogenous mammalian	-1.69
chemical - endogenous mammalian	-1.69
chemical - endogenous mammalian	-1.7
chemical - endogenous mammalian	-1.701
chemical - endogenous mammalian	-1.707
chemical - endogenous mammalian	-1.736
chemical - endogenous mammalian	-1.739
chemical - endogenous mammalian	-1.739
chemical - endogenous mammalian	-1.743
chemical - endogenous mammalian	-1.754
chemical - endogenous mammalian	-1.773
chemical - endogenous mammalian	-1.799
chemical - endogenous mammalian	-1.816
chemical - endogenous mammalian	-1.828
chemical - endogenous mammalian	-1.844
chemical - endogenous mammalian	-1.858
chemical - endogenous mammalian	-1.885
chemical - endogenous mammalian	-1.938
chemical - endogenous mammalian	-1.967
chemical - endogenous mammalian	-1.985
chemical - endogenous mammalian	-2.031
chemical - endogenous mammalian	-2.092
chemical - endogenous mammalian	-2.175
chemical - endogenous mammalian	-2.178
chemical - endogenous mammalian	-2.181
chemical - endogenous mammalian	-2.181
chemical - endogenous mammalian	-2.195
chemical - endogenous mammalian	-2.218
chemical - endogenous mammalian	-2.28
chemical - endogenous mammalian	-2.402
chemical - endogenous mammalian	-2.646
chemical - endogenous mammalian	-2.662
chemical - endogenous mammalian	-2.972
chemical - endogenous mammalian	-3.091
chemical - endogenous mammalian	-4.761

Table S5

Radiation	Day post-radiation	Pathway	Z-score
9.5 Gy	1d	Release of fatty acid	-2.22
		Concentration of D-glucose	-1.98
		Quantity of carbohydrate	-1.97
		Release of lipid	-1.66
		Peroxidation of lipid	-1.52
		Synthesis of nucleotide	1.56
		Binding of DNA	1.8
		Influx of Ca2+	1.94
		Synthesis of cyclic AMP	2.11
	3d	Efflux of L-amino acid	-2.24
		Neurotransmission	-2.23
		Differentiation of macrophages	-2.22
		Growth of bacteria	-2.14
		Synaptic transmission	-2.03
		Efflux of L-alanine	-2
		Apoptosis of neuroblastoma cell lines	-1.98
		Accumulation of triacylglycerol	-1.98
		Excitation of brain cells	-1.97
		Excitation of cells	-1.78
		Apoptosis of vascular endothelial cells	-1.76
		Release of hormone	-1.71
		Growth of organism	-1.71
		Excitation of neurons	-1.56
		Stimulation of central nervous system cells	-1.51
		Migration of granulocytes	1.51
		Secretion of lipid	1.52
		Tyrosine phosphorylation of protein	1.53
		Fatty acid metabolism	1.54
		Cell movement of natural killer cells	1.56
		Cell movement of granulocytes	1.57
		Migration of mononuclear leukocytes	1.57
		Inflammation of absolute anatomical region	1.64
		Interaction of protein	1.66

		Influx of Ca2+	1.67
		Transport of monosaccharide	1.7
		Synthesis of lipid	1.76
		Synthesis of prostaglandin E2	1.78
		Flux of Ca2+	1.83
		Inflammation of organ	1.88
		NK cell migration	1.94
		Cell movement of leukocyte cell lines	1.95
		Neurodegeneration of brain cells	1.98
		Migration of endothelial cell lines	1.98
		Adhesion of immune cells	2.06
		Synthesis of cyclic AMP	2.09
		Adhesion of lymphocytes	2.22
		Uptake of L-alanine	2.45
		Uptake of L-amino acid	2.62
		Uptake of amino acids	2.77
	9d	Release of fatty acid	-2.43
		Lipolysis	-1.99
		Concentration of cholesterol ester	-1.98
		Generation of superoxide	-1.96
		Activation of macrophages	-1.95
		Release of lipid	-1.94
		Accumulation of triacylglycerol	-1.77
		Inflammatory response	-1.56
		Synthesis of polysaccharide	1.95
		Oxidation of monosaccharide	1.96
		Transport of D-glucose	1.98
		Efflux of phospholipid	1.98
		Export of phospholipid	1.98
		Uptake of L-amino acid	2
	1d	Activation of macrophages	-1.98
		Activation of blood cells	-1.76
		Concentration of cyclic AMP	1.78
		Cell viability of hepatoma cell lines	-2
		Excitation of neurons	-1.88

11Gy	3d	Accumulation of cells	-1.83
		Mobilization of Ca2+	-1.73
		Concentration of fatty acid	-1.68
		Stimulation of cells	-1.53
		Transport of molecule	1.54
		Apoptosis of hepatoma cell lines	1.62
		Biosynthesis of cyclic nucleotides	1.63
		Accumulation of cyclic AMP	1.64
		Cell death of hepatoma cell lines	1.67
		Influx of Ca2+	1.67
		Quantity of ceramide	1.68
		Apoptosis of smooth muscle cells	1.7
		Inflammation of organ	1.72
		Cell death of antigen presenting cells	1.87
		Uptake of L-alanine	1.89
		Flux of Ca2+	1.94
		Cell movement of leukocyte cell lines	1.95
		Biosynthesis of amide	1.96
		Synthesis of cyclic AMP	1.96
		Uptake of L-triiodothyronine	1.98
		Mineralization of cells	1.98
		Quantity of sphingolipid	1.99
		Uptake of L-proline	2
		Flux of inorganic cation	2.1
		Synthesis of reactive oxygen species	2.2
		Metabolism of reactive oxygen species	2.34
		Concentration of eicosanoid	-2.73
		Proliferation of CD4+ T-lymphocytes	-2.24
		Differentiation of macrophages	-2.22
		Conditioning	-2.21
		Apoptosis of pancreatic cancer cell lines	-2.21
		Necrosis of epithelial tissue	-2.19
		Secretion of molecule	-2.17
		Concentration of prostaglandin	-2.16
		Apoptosis of endothelial cells	-2.15

		Release of eicosanoid	-2.14
		Synthesis of prostaglandin E2	-2.13
		Synthesis of eicosanoid	-2.06
		Transport of H+	-2
		Concentration of anandamide	-1.98
		Response of chorda tympani	-1.96
		Release of hormone	-1.96
		Synthesis of purine nucleotide	-1.95
		Apoptosis of vascular endothelial cells	-1.93
		Concentration of fatty acid	-1.91
		Quantity of nitric oxide	-1.89
		Secretion of lipid	-1.84
		Hepatic steatosis	-1.77
	d9	Apoptosis of epithelial cells	-1.76
		Consumption of oxygen	-1.76
		Release of lipid	-1.75
		Concentration of Ca2+	-1.68
		Accumulation of lipid	-1.66
		Production of hydrogen peroxide	-1.66
		Incorporation of fatty acid	-1.64
		Excitation of cells	-1.64
		Accumulation of acylglycerol	-1.61
		Mobilization of Ca2+	-1.56
		Lipolysis	-1.54
		Release of fatty acid	-1.53
		Cell death of epithelial cells	-1.52
		Entrance of Ca2+	-1.52
		Cell death of cerebral cortex cells	1.53
		Neuronal cell death	1.6
		Cell death of central nervous system cells	1.62
		Steroidogenesis of hormone	1.66
		Maturation of cells	1.75
		Cell death of brain cells	1.77
		Concentration of cyclic AMP	1.81
		Synthesis of cyclic AMP	1.86
